# Unraveling genotype–phenotype relationships in hereditary hemochromatosis through integrated biobank data analysis

**DOI:** 10.1186/s12864-026-12746-3

**Published:** 2026-03-16

**Authors:** Miriam Nurm, Tarmo Annilo, Sebastian May-Wilson, Anu Reigo, Reedik Mägi, Urmo Võsa, Neeme Tõnisson, Estonian Biobank Research Team, Andres Metspalu, Lili Milani, Tõnu Esko, Mari Nelis, Georgi Hudjashov, Toomas Haller

**Affiliations:** 1 Estonian Genome Center, Institute of Genomics, University of Tartu, Tartu, Estonia; 2 Institute for Molecular Medicine Finland (FIMM), Helsinki Institute of Life Science (HiLIFE), University of Helsinki, Helsinki, Finland; 3 Genetics and Personalized Medicine Clinic, Tartu University Hospital, Tartu, Estonia

**Keywords:** Hereditary hemochromatosis, Phenome-wide association study, Genome-wide association study, Biobank, Electronic health records

## Abstract

**Background:**

Type I hereditary hemochromatosis (HH), caused by pathogenic *HFE* variants, is among the most common autosomal recessive disorders in Northern Europe. HH genotype–phenotype associations have been difficult to predict due to variable variant penetrance and expressivity. In this study, population-based biobank data were used to conduct a large-scale analysis of symptoms associated with different HH genotypes and their potential genetic modifiers.

**Methods:**

Linked genotypic and electronic health records from the Estonian Biobank (*n* = 211,994) and UK Biobank (*n* = 405,931) were used to investigate HH genotype–phenotype associations. Pathogenic variants of all HH types in the Estonian Biobank sample were identified. Clinical markers derived from laboratory measurements and diagnosis codes were compared between *HFE* pathogenic variant carriers and controls [chi-squared test, phenome-wide association study (PheWAS)]. Carrier subgroups were defined according to the presence of p.C282Y, p.H63D, p.S65C, and all pairwise combination genotypes. Kruskal–Wallis and Mann–Whitney *U* tests were used to identify ICD-10 codes and genotype groups with significantly different mean ages at first diagnosis. Genome-wide association studies (GWAS) based on ceruloplasmin and ferritin concentrations were conducted to identify potential genetic modifiers. Fine-mapping was used to identify putatively causal single nucleotide polymorphisms (credible sets) in the Estonian Biobank GWAS results. Fixed-effects meta-analyses of combined GWAS results from both biobanks were performed.

**Results:**

We conducted the largest PheWAS with data from *HFE* variant carriers to date and catalogued pathogenic HH variants in the Estonian population. p.S65C homozygotes and p.C282Y/p.S65C heterozygotes in the Estonian Biobank sample were diagnosed with urogenital conditions (ICD-10 codes N42 and N50.8) at significantly higher rates than were controls. On average, N42 was first diagnosed 11 years earlier in p.S65C compound heterozygotes and homozygotes than in controls. We also identified a novel GWAS hit, *CP* rs61733458, with a significant impact on the ceruloplasmin level and ties to iron metabolism–related diseases.

**Conclusions:**

Our results suggest that the p.S65C variant is more clinically relevant than previously thought and should be integrated more into HH testing guidelines. *CP* variant rs61733458 is a potential genetic modifier associated with iron metabolism–related diseases.

**Supplementary Information:**

The online version contains supplementary material available at 10.1186/s12864-026-12746-3.

## Background

Hereditary hemochromatosis (HH) is a common iron overload disorder characterized by notable phenotypic diversity and incomplete penetrance of HH-associated gene variants. The disease affects about 1 in 150–300 adults of Northern European descent [1]. Up to five different HH subtypes have been identified: three autosomal recessive subtypes involving the *HFE* (type I) [2], *HJV*/*HAMP* (type II) [3], and *TFR2* (type III) [4] genes and two autosomal dominant subtypes associated with the *SLC40A1* (type IV) [5] and *FTH1* (type V) [6] genes. HH type I, caused by pathogenic variants in the *HFE* gene on chromosome 6, is considered to be the most common subtype [7]. Three clinically relevant pathogenic *HFE* variants have been described: p.C282Y, p.H63D, and p.S65C. p.C282Y homozygotes have been found to account for about 95% of all HH cases and p.C282Y/H63D compound heterozygotes (CHs) have been found to account for about 4% of cases [8]; thus far, p.S65C penetrance estimates have been inconclusive [9].

HH has a diverse spectrum of symptoms that tend to be non-specific (e.g., lethargy and arthralgia [10]), resulting in frequent mis- or underdiagnosis of the disease. The primary target organs of excess iron accumulation are the liver, pancreas, and heart, causing liver disease, *diabetes mellitus*, and arrhythmias/heart failure, respectively. Additionally, adult-onset hypogonadism manifesting as decreased sex drive, erectile dysfunction or amenorrhea, and skin hyperpigmentation may be observed in patients with HH [11]. Initial HH symptoms reported most frequently by patients include extreme fatigue, joint pain, and loss of libido or impotence [12–14].

Alongside transferrin, ferritin is the most important clinical biomarker in HH diagnostics, reflecting the level of iron overload in the body [15]. The saturation level of the iron-binding protein transferrin may already be increased in the early stages of HH [16]. Ferritin acts as an iron repository and a protective factor against oxidative stress. In addition to iron metabolism disorders, it plays a role in inflammatory and autoimmune diseases [17]. Ceruloplasmin, a clinical biomarker not currently included in routine HH testing, plays a key role in copper and iron metabolism due to its ferroxidase activity and other anti- and pro-oxidant properties [18]. Low ceruloplasmin levels have been associated with various pathological conditions, such as aceruloplasminemia [19], neurodegenerative diseases [20], and liver disease [21].

As the early recognition and diagnosis of HH could prevent severe health complications and reduce the burden on healthcare resources, the improvement of HH phenotype and genotype screening is vital [22]. To establish a data-driven foundation for future HH genetic testing and diagnostic guidelines, we assessed the populational prevalence of HH-associated pathogenic variants and analyzed the clinical penetrance of the three most common *HFE* variants in the Estonian Biobank (EstBB) cohort. We further investigated the roles of potential genetic modifiers of the HH phenotype through genome-wide association studies (GWAS) using EstBB and UK Biobank (UKB) data.

## Materials and methods

### Data sources

The EstBB holds genotypic and phenotypic data (linked electronic health records (EHRs)) for more than 210,000 participants (about 20% of Estonia’s adult population). As participants provided broad written consent upon joining, the EstBB has the right to re-contact them and update their data through linkage with EHRs and national health registries [23]. Thus, retrograde (from 2004) and anterograde health records are available.

Genotype data were derived from direct genotyping using the Infinium Global Screening Array (v1.0 and 2.0; Illumina Inc., San Diego, CA, USA) and further imputed for a total of 211,994 individuals. Next-generation sequencing data were available for 4,776 individuals (whole-exome sequencing (WES), *n* = 2,356; whole-genome sequencing (WGS), *n* = 2,420). The genotype and next-generation sequencing data were used for variant analyses. Technical details on direct genotyping, genotype imputation, and annotation of EstBB data have been published previously [24].

**Fig. 1:**
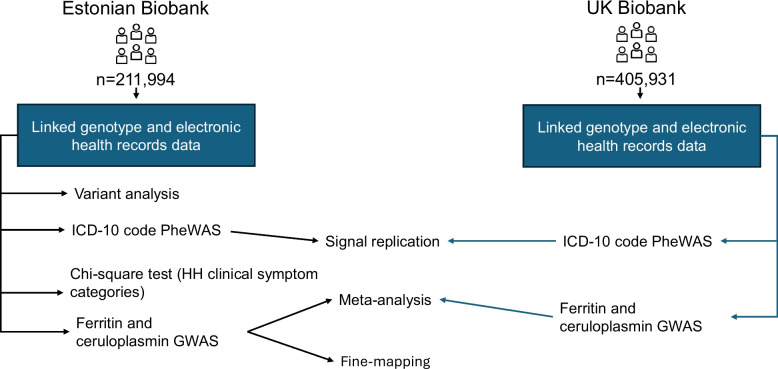
Overview of Estonian Biobank and UK Biobank data analysis. ICD-10, International Classification of Diseases, 10th Revision; PheWAS, phenome-wide association study; HH, hereditary hemochromatosis; GWAS, genome-wide association study

The UKB holds data on more than 500,000 individuals aged 37–73 years from across the United Kingdom, recruited between 2006 and 2010. The recruitment process, participants, and quality control procedures have been described previously [25]. All participants provided written informed consent upon joining the biobank. Genotyping of 49,950 participants in the UK Biobank Lung Exome Variant Evaluation (UK BiLEVE) study was conducted using the Applied Biosystems™ UK BiLEVE Axiom™ Array; 438,427 participants were genotyped using the UK Biobank Axiom™ Array (Affymetrix, Santa Clara, CA, USA). Additional details on genotyping, marker selection, and sample quality control have been published previously [26]. An overview of the study design is provided in Fig. 1.

### Statistical analyses

Specific parameters, software versions used, and more detailed descriptions of the analyses are provided in Additional File 1: Supplementary Material S1. Only EstBB participants designated as p.C282Y, p.H63D, and p.S65C carriers in both the directly genotyped and imputed datasets were included in the analyses. The chi-squared test was used to examine the occurrence of 23 pre-selected common HH symptom categories in these genotype groups. Fisher’s exact test was used for the retesting of categories in which > 20% of expected values were < 5 (the chi-squared test threshold requirement) (Additional File 2: Table S1). The original chi-squared test *p* value was considered to be legitimate when at least one group in the category showed significance [false discovery rate (FDR)-adjusted *p* < 0.05] against controls. All individuals diagnosed with iron metabolism disorders [International Classification of Diseases, 10th Revision (ICD-10) code E83.1] were excluded from the control group. The same control group was used for all symptom categories except in the category ‘Iron metabolism disorders E83.1’, for which the number of excluded controls was used as no individuals with E83.1 were present in the control group.

ICD-10 codes identified as statistically significant in the phenome-wide association studies (PheWAS) and subsequently aggregated based on the phecodeX hierarchy [27] were analyzed using the Kruskal–Wallis test to detect code aggregates differing significantly according to the age at first diagnosis. Only code aggregates representing more than 10 individuals in each group were included in this analysis. The Mann–Whitney *U* test was used to identify significant differences between genotype groups according to the age at first diagnosis. The control groups included in the Kruskal–Wallis and Mann–Whitney analyses were derived from the control group used in the chi-squared analysis. Bonferroni correction for multiple testing was used in the chi-squared analysis, and Benjamini–Hochberg (FDR-based) correction was used in all other analyses.

### PheWAS

The EstBB PheWAS were run in R (v4.1.3) using the glm() function, with the sex, year of birth, and first 10 principal components serving as covariates. Only ICD-10 codes recorded for at least five separate individuals were included in the PheWAS. The aggregated ICD-10 codes were ranked according to the proportion of affected individuals in each PheWAS group in which the individual ICD-10 code(s) showed significant associations. Before PheWAS analysis, first- and second-degree relatives were removed from the case and control groups, the latter comprising of the rest of the biobank participants. The PheWAS were carried out in two batches, testing first for the occurrence of the main three-character ICD-10 codes (e.g., E83) and then for the extended subcodes (e.g., E83.1). Specifics of the UKB PheWAS can be found in Additional File 1: Supplementary Material S1.

### GWAS

REGENIE [28] was used to perform all EstBB and UKB GWAS, with the age, age squared, sex, and first 10 genotype principal components serving as covariates. In the EstBB GWAS, rank inverse normal transformation was applied in REGENIE.

### Post-GWAS analyses

GWAS signals were annotated using the Functional Mapping and Annotation of Genome-Wide Association Studies (FUMA) platform (v1.6.1) [29]. The EstBB and UKB GWAS results were entered into a fixed-effects meta-analysis using METAL (v2020-05–05) [30]. Fine- mapping of EstBB GWAS variants was performed using SuSiE (v0.14.2) [31] and LDstore2 (v2.0) [32] with a pipeline specifically adapted for EstBB data (described elsewhere [33]).

## Results

### HH-related variants in the EstBB data

To estimate the genetic prevalence of HH and its subtypes in the EstBB sample, we used participants’ genotype data. Variants pooled from directly genotyped, imputed, and WES/WGS datasets were filtered for the identification of potentially pathogenic HH-associated variants in *HFE*, *TFR2*, *HJV*, *HAMP*, *SLC40A1*, and *FTH1* for all HH types, as described in the Online Mendelian Inheritance in Man catalog [2]. In addition to the three well-known *HFE* variants p.C282Y, p.H63D, and p.S65C, variants with combined annotation dependent deletion (CADD) scores ≥ 15 and minor allele frequencies (MAFs) < 0.037 (the MAF for p.C282Y, the most clinically penetrant *HFE* variant, in directly genotyped carriers) were added to the potential variant pool. Variants designated as benign and those with conflicting status and no pathogenic or likely pathogenic designation in the ClinVar database [34] were excluded. Variants from the WES/WGS dataset with quality score < 500 were also excluded.

The filtering of HH-related variants in the EstBB genotype data yielded 24 potentially pathogenic variants in the *HFE* (*n* = 6), *TFR2* (*n* = 11), *HJV* (*n* = 2), *SLC40A1* (*n* = 4), and *FTH1* (*n* = 1) genes; no pathogenic *HAMP* variant was detected in this sample (Additional File 3:Table S2). Three of these variants (*SLC40A1* c.121 T > A, *HFE* c.737 T > G, and *TFR2* c.95G > T) have not, to our knowledge, been reported previously. All variants were missense variants, except for one loss-of-function variant (*FTH1* p.Glu148*) resulting in a premature stop codon. Besides *HFE* p.C282Y, p.H63D, and p.S65C, the variants *HJV* p.C80R and p.G320V were designated as pathogenic, and *TFR2* p.R678P was designated as likely pathogenic in ClinVar; the others were classified as variants of uncertain significance or not recorded in ClinVar. No CH combination excluding p.C282Y, p.H63D, and p.S65C was detected.

We calculated allele frequencies for the p.C282Y, p.H63D, and p.S65C variants in carriers (designated as such in both the directly genotyped and imputed datasets) and compared them with publicly available MAF estimates from Estonia and neighboring countries. Estimated allele frequencies for p.C282Y, p.H63D, and p.S65C in the EstBB sample were 3.7%, 14.2%, and 0.9%, respectively (Additional File 3: Table S2).

### Clinical symptom categories in *HFE* variant carriers

We estimated the clinical penetrance of the three most prevalent and clinically relevant *HFE* variants (C282Y, p.H63D, and p.S65C) by utilizing EHR data to analyze the frequency of well-documented HH symptoms and comorbidities [35, 36]. A curated list of symptom categories for these conditions with corresponding ICD-10 codes was compiled.

The carriers were matched with best-fitting individuals from the remaining biobank participants in terms of age, sex, and body mass index at a 1:2 ratio to form a control group (*n* = 138,607). Individuals diagnosed with iron metabolism disorders (code E83.1) were excluded from the control group. The carriers were then divided into nine genotype groups: p.C282Y homozygotes (*n* = 290), p.H63D homozygotes (*n* = 4,159), p.S65C homozygotes (*n* = 32), p.C282Y/H63D CHs (*n* = 2,042), p.C282Y/p.S65C CHs (*n* = 123), p.S65C/H63D CHs (*n* = 166), p.C282Y simple heterozygotes (*n* = 12,112), p.H63D simple heterozygotes (*n* = 46,894), and p.S65C simple heterozygotes (*n* = 3,430).

A chi-squared test was conducted to examine the occurrence of each symptom category in the genotype and control groups (Table 1). All laboratory measurements were retrieved from EHRs and preprocessed as described in Additional File 1: Supplementary Material S1.

**Table 1:** Occurrence of HH-associated clinical symptoms in *HFE* p.C282Y, p.H63D, and p.S65C genotype groups and controls

**Clinical symptom categories**	**C282Y**				**H63D**			**S65C**			***p***-value
	**C282Y (*n* = 290)**	**S65C (*n* = 123)**	**H63D (*n* = 2042)**	**REF (*n* = 12,112)**	**H63D (*n* = 4159)**	**S65C (*n* = 166)**	**REF (*n* = 46,894)**	**S65C (*n* = 32)**	**REF (*n* = 3430)**	**controls (*n* = 138,607)**	
Females (%)	192 (66.2%)	77 (62.6%)	1316 (64.5%)	7945 (65.6%)	2742 (65.9%)	114 (68.7%)	30,761 (65.6%)	19 (59.4%)	2269 (66.2%)	90,941 (65.6%)	
Median age ± SD (range, yrs)	52 ± 16 (23–90)	51 ± 16 (23–89)	51 ± 17 (23–99)	51 ± 16 (20–103)	52 ± 17 (23–102)	51 ± 17 (24–97)	52 ± 17 (20–107)	50 ± 16 (29–96)	52 ± 16 (21–103)	52 ± 16 (20–106)	
Mean BMI ± SD (range)	27 ± 6 (18–56)	27 ± 6 (19–49)	27 ± 6 (16–68)	27 ± 6 (15–66)	27 ± 6 (15–83)	27 ± 7 (17–75)	27 ± 6 (14–77)	26 ± 5 (20–39)	27 ± 6 (15–67)	27 ± 6 (11–91)	
Iron metabolism disorders E83.1	37 (12.8%)	0 (0.0%)	12 (0.6%)	27 (0.2%)	20 (0.5%)	0 (0.0%)	60 (0.1%)	0 (0.0%)	5 (0.1%)	0 (0.0%)	< *1* × *10*^*–16,a*^
Copper metabolism disorders E83.0	3 (1.0%)	0 (0.0%)	3 (0.1%)	4 (0.0%)	2 (0.0%)	0 (0.0%)	13 (0.0%)	0 (0.0%)	0 (0.0%)	47 (0.0%)	*3.45* × *10*^*−16,a*^
Ferritin (F > 150, M > 400 ug/L)	31 (10.7%)	6 (4.9%)	97 (4.8%)	389 (3.2%)	161 (3.9%)	1 (0.6%)	1613 (3.4%)	1 (3.1%)	122 (3.6%)	4279 (3.1%)	2.65 × 10^–16,a^
Congestive heart failure I50*	25 (8.6%)	12 (9.8%)	220 (10.8%)	1240 (10.2%)	462 (11.1%)	18 (10.8%)	4798 (10.2%)	2 (6.2%)	357 (10.4%)	13,419 (9.7%)	0.002^b^
Abdominal pain R10.1, R10.3, R10.4	80 (27.6%)	43 (35.0%)	611 (29.9%)	3815 (31.5%)	1345 (32.3%)	52 (31.3%)	14,271 (30.4%)	14 (43.8%)	1103 (32.2%)	42,538 (30.7%)	0.022^b^
Dyslipidemia E78*	80 (27.6%)	38 (30.9%)	626 (30.7%)	3848 (31.8%)	1349 (32.4%)	48 (28.9%)	15,337 (32.7%)	10 (31.2%)	1139 (33.2%)	45,733 (33.0%)	0.034^b^
Cardiomyopathy I42*, I43.1, I43.8, I51.4	8 (2.8%)	5 (4.1%)	106 (5.2%)	680 (5.6%)	239 (5.7%)	9 (5.4%)	2410 (5.1%)	2 (6.2%)	161 (4.7%)	7559 (5.5%)	0.050
Other non-specific liver conditions R16.0, R17*, R93.2, R94.5, R79.0	3 (1.0%)	3 (2.4%)	18 (0.9%)	126 (1.0%)	32 (0.8%)	2 (1.2%)	442 (0.9%)	0 (0.0%)	36 (1.0%)	1146 (0.8%)	0.071
Arthritis M15*-M19*, M01*	111 (38.3%)	50 (40.7%)	749 (36.7%)	4588 (37.9%)	1550 (37.3%)	62 (37.3%)	17,749 (37.8%)	16 (50.0%)	1268 (37.0%)	51,464 (37.1%)	0.172
Other chronic liver disease K73*, K72.1	2 (0.7%)	2 (1.6%)	18 (0.9%)	120 (1.0%)	56 (1.3%)	3 (1.8%)	431 (0.9%)	0 (0.0%)	43 (1.3%)	1368 (1.0%)	0.177
Diabetes E11*-E14*	30 (10.3%)	6 (4.9%)	197 (9.6%)	1036 (8.6%)	349 (8.4%)	16 (9.6%)	4218 (9.0%)	1 (3.1%)	317 (9.2%)	12,116 (8.7%)	0.219
Liver disease K70.0, K71*-K76*, K77.8	18 (6.2%)	5 (4.1%)	149 (7.3%)	862 (7.1%)	287 (6.9%)	14 (8.4%)	3063 (6.5%)	0 (0.0%)	234 (6.8%)	9369 (6.8%)	0.221
Impotence and loss of sex drive N48.4, Z70.1, F52*	8 (2.8%)	7 (5.7%)	97 (4.8%)	523 (4.3%)	197 (4.7%)	5 (3.0%)	1997 (4.3%)	4 (12.5%)	150 (4.4%)	6062 (4.4%)	0.240
HbA1C ≥ 5.9%	24 (8.3%)	9 (7.3%)	217 (10.6%)	1227 (10.1%)	414 (10.0%)	21 (12.7%)	5061 (10.8%)	2 (6.2%)	374 (10.9%)	14,651 (10.6%)	0.254
Liver cirrhosis K74*	3 (1.0%)	0 (0.0%)	7 (0.3%)	68 (0.6%)	21 (0.5%)	0 (0.0%)	187 (0.4%)	0 (0.0%)	13 (0.4%)	627 (0.5%)	0.276
Liver enzyme elevation > 6mo	15 (5.2%)	2 (1.6%)	96 (4.7%)	579 (4.8%)	224 (5.4%)	11 (6.6%)	2212 (4.7%)	3 (9.4%)	170 (5.0%)	6722 (4.8%)	0.358
Hypertriglyceridemia E78.1	2 (0.7%)	0 (0.0%)	17 (0.8%)	86 (0.7%)	36 (0.9%)	0 (0.0%)	336 (0.7%)	0 (0.0%)	34 (1.0%)	939 (0.7%)	0.364
Brain perfusion disorders I65*- I66*, I67.2	3 (1.0%)	3 (2.4%)	44 (2.2%)	240 (2.0%)	83 (2.0%)	2 (1.2%)	1044 (2.2%)	2 (6.2%)	79 (2.3%)	2933 (2.1%)	0.406
Short stature (Z < −2)	4 (1.4%)	3 (2.4%)	39 (1.9%)	278 (2.3%)	99 (2.4%)	2 (1.2%)	1159 (2.5%)	1 (3.1%)	82 (2.4%)	3219 (2.3%)	0.572
Liver cancer (hepatocellular carcinoma, cholangiocarcinoma) C22*, C24*, D01.5, D13.4, D37.6	2 (0.7%)	1 (0.8%)	11 (0.5%)	75 (0.6%)	30 (0.7%)	1 (0.6%)	328 (0.7%)	1 (3.1%)	30 (0.9%)	908 (0.7%)	0.601
Ceruloplasmin (F < 0.16, M < 0.15 g/L)	1 (0.3%)	0 (0.0%)	3 (0.1%)	13 (0.1%)	6 (0.1%)	0 (0.0%)	41 (0.1%)	0 (0.0%)	1 (0.0%)	128 (0.1%)	*0.736*
Fatigue F06.6, F48.0, R53*	40 (13.8%)	15 (12.2%)	292 (14.3%)	1735 (14.3%)	575 (13.8%)	19 (11.4%)	6622 (14.1%)	5 (15.6%)	478 (13.9%)	19,904 (14.4%)	0.885
Joint pain M02*, M25.5	116 (40.0%)	51 (41.5%)	798 (39.1%)	4781 (39.5%)	1639 (39.4%)	71 (42.8%)	18,297 (39.0%)	12 (37.5%)	1335 (38.9%)	54,544 (39.4%)	0.951

Controls were matched to cases based on age, sex and BMI. *P* values were obtained using the chi-squared test; italicized values have been retested using Fisher's exact test

*HH* Hereditary hemochromatosis, *SD* Standard deviation, *BMI* Body mass index, *HbA1c* glycosylated hemoglobin

^*^The ICD-10 code and all of its subcodes were used

^a^The result was statistically significant after Bonferroni correction

^b^The result was nominally significant

Six of the 23 clinical symptom categories showed nominally significant differences between the carrier and control groups (*p* < 0.05, chi-squared test). After the application of multiple testing correction, differences in three categories—iron metabolism disorders, copper metabolism disorders, and ferritin levels above the reference—remained significant. Abdominal pain and dyslipidemia were found to be nominally significant but were no longer considered significant after multiple testing correction. Additionally, the value for congestive heart failure fell narrowly outside of the multiple testing threshold (0.0025 vs. 0.0022) and that for cardiomyopathy fell narrowly outside of the nominal significance threshold (0.0501 vs. 0.05).

### PheWAS highlights the broadness of the HH symptom spectrum and clinical relevance of the p.S65C variant

To identify potential genotype–phenotype associations for the HH genotype groups, PheWAS were conducted using ICD-10 codes. In addition to the nine genotype groups listed above, five other groups were included in the PheWAS analysis: all p.C282Y carriers (*n* = 14,567), all p.H63D carriers (*n* = 53,261), all p.S65C carriers (*n* = 3,751), all CHs/alternative homozygotes (AHs) of these three variants (*n* = 6,812), and all carriers of the three variants together (*n* = 69,398). Carriers were defined as all individuals carrying at least one copy of the variant (all AHs, CHs, and simple heterozygotes).

To better interpret the clinical significance of the diagnostic codes in our PheWAS results, all ICD-10 main codes and subcodes that remained significant after multiple testing correction were clustered using the phecodeX hierarchical classification system [27]. The purpose of this clustering was to better capture clinically relevant signals beyond the ICD-10 coding structure. Main codes were defined as the 3-character ICD-10 code consisting of a letter and 2 numbers whereas subcodes are extensions of the main codes where additional number(s) are separated by a decimal point. The phecodeX system combines related codes from different ICD-10 chapters into group diagnoses with similar clinical meaning; for example, the C43.4/D03.8/C43.0/D03 grouping represents “melanomas of skin”. The ICD-10 code aggregates belong to larger overarching categories in the phecodeX hierarchy, such as ‘Congenital’ and ‘Cardiovascular’. Code aggregates may contain single ICD-10 codes.

The 14 PheWAS yielded 217 unique ICD-10 codes (231 codes in total; 37 main codes and 180 subcodes) that remained significant after multiple testing correction (Fig. 2; Additional File 1: Supplementary Figs. S3–S28; Additional File 4: Table S3). The genotype group with the largest number of significant codes was p.H63D/p.S65C CH (*n* = 59), followed by p.C282Y AH (*n* = 56) and p.S65C AH (*n* = 49). Five genotype groups had no significant ICD-10 code: the simple heterozygote groups for the three variants (C282Y, p.H63D, and p.S65C), all p.H63D carriers, and all p.S65C carriers. Additionally, the p.C282Y/H63D CH group had no significant main codes, and the group composed of all three carriers had no significant subcodes.

**Fig. 2:**
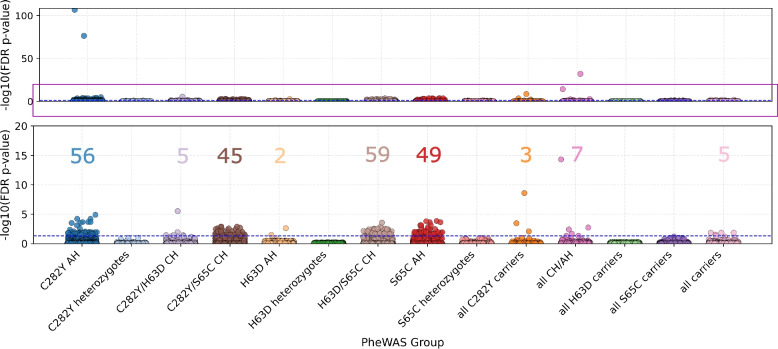
Distribution of significant ICD-10 codes among PheWAS groups. The number of significant ICD-10 codes detected in each PheWAS group is represented by a matching colored numeral. ICD-10, International Classification of Diseases, 10th Revision; PheWAS, phenome-wide association study; AH, alternative homozygote; CH, compound heterozygote

Sixty-four ICD-10 codes (belonging to mostly non-diagnostic S-Z categories) were excluded from phecodeX mapping due to missing matches (no ICD-10 code to phecode equivalence found), and three were manually corrected to the ICD-10 codes medically closest to phecodeX matches (G21.0, O05.0, and O05.8 to G21.1, O08, and O08, respectively). The remaining 152 ICD-10 codes were mapped into 157 aggregated code combinations (some codes were used more than once) with 139 unique aggregates based on phecodeX mapping. The phecodeX categories with the most ICD-10 code aggregates were Musculoskeletal (*n* = 16), Infections (*n* = 16), Congenital (*n* = 15), Neoplasms (*n* = 14), and Cardiovascular (*n* = 12).

**Fig. 3:**
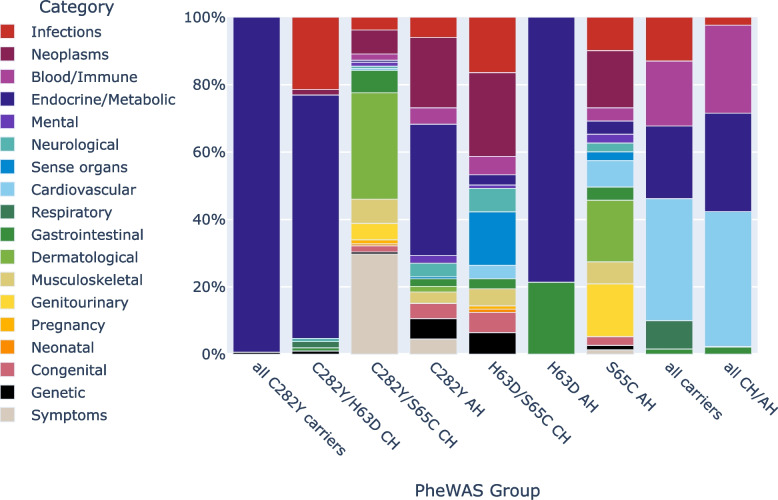
Most prevalent ICD-10 code aggregate categories in the *HFE* p.C282Y, p.H63D, and p.S65C PheWAS cohorts. The frequencies (percentages of affected individuals) of each significant ICD-10 code aggregate in its respective category have been summed, with the total represented as 100%. ICD-10, International Classification of Diseases, 10th Revision; PheWAS, phenome-wide association study; CH, compound heterozygote; AH, alternative homozygote

The C282Y/p.S65C CH, p.S65C AH, and p.H63D/p.S65C CH groups were associated with the largest numbers of phecodeX categories (Fig. 3). The p.C282Y and p.S65C AH groups were associated with remarkably diverse phecode categories, whereas the p.H63D homozygote group was associated significantly with ICD-10 code aggregates in only two categories: Endocrine/Metabolic (E83.1) and Gastrointestinal (K27/K27.3; peptic ulcer). Similar categories were prevalent in the groups of all CHs/AHs and all carriers (Infections, Blood/Immune, Endocrine/Metabolic, Cardiovascular, and Gastrointestinal), but the Respiratory category, consisting of the ICD-10 code J18 (unspecified pneumonia), was distinctly more prevalent in the group of all carriers (8.4% vs. 0.2%). Codes in the Dermatological category were most prevalent in the p.S65C AH and p.C282Y/p.S65C CH groups. The Infections category was associated with all PheWAS groups except for all p.C282Y carriers and p.H63D AHs.

The ICD-10 code aggregates affecting the largest percentages of individuals in the genotype groups consisted of 25 unique items affecting 3.5–42.2% of individuals (only codes affecting > 3.33% of individuals were taken into account; Table 2). The code aggregate affecting the largest percentage of individuals in any genotype group was L23/L23.5 (contact dermatitis and allergies related to other diseases/symptoms; 42.2%). Other prevalent codes included N42 (disorders of the prostate; 38.5% of p.S65C homozygote males), I10 and I11 (hypertension without and with heart disease; 35.6% and 26.9%, respectively), and D18 (hemangioma and lymphangioma; 23.3%). D50 (iron deficiency anemia), which was among the most frequent code aggregates (affecting 20.4% of all carriers), was the only ICD-10 code with a negative beta coefficient, indicating reduced occurrence compared with that in the rest of the biobank cohort.

**Table 2:** ICD-10 code aggregates appearing most frequently in any PheWAS genotype group

**ICD-10 code aggregate**	**C282Y**				**H63D**		**S65C**								
	**C282Y**	**H63D**	**S65C**	**all carriers**	**H63D**	**S65C**	**S65C**	**all CH/AH**	**all carriers**	**genotype group count**	**Average %**	**Highest %**	**category**	**Corresponding phecode**	**phecode description**
L23/L23.5	0	0	2	0	0	0	0	0	0	1	24.6	42.2	Dermatological	DE_668.3	Contact dermatitis/Allergies related to other diseases/symptoms
L23/L23.5	0	0	2	0	0	0	0	0	0	1	24.6	42.2	Symptoms	SS_840.8	Contact dermatitis/Allergies related to other diseases/symptoms
N42	0	0	0	0	0	0	1	0	0	1	38.5*	38.5*	Genitourinary	GU_602	Disorders of prostate
I10	0	0	0	0	0	0	0	1	0	1	35.7	35.7	Cardiovascular	CV_401.1	Essential hypertension
I11	0	0	0	0	0	0	0	1	0	1	26.9	26.9	Cardiovascular	CV_401.2	Hypertensive heart disease
D18	0	0	0	0	0	0	1	0	0	1	23.3	23.3	Neoplasms	CA_139.6	Hemangioma and lymphangioma
D50(↓)	0	0	0	0	0	0	0	1	1	2	19.7	20.4	Blood/Immune	BI_160.1	Iron deficiency anemia
D50(↓)	0	0	0	0	0	0	0	1	1	2	19.7	20.4	Blood/Immune	BI_164.1	Iron deficiency anemia
D50(↓)	0	0	0	0	0	0	0	1	1	2	19.7	20.4	Endocrine/Metabolic	EM_247.88	Iron deficiency anemia
L81	0	0	0	0	0	0	1	0	0	1	20.0	20.0	Dermatological	DE_674	Disorders of pigmentation
N34/N34.1	0	0	0	0	0	0	2	1	0	2	16.7	20.0	Genitourinary	GU_592.2	Urethritis
J18	0	0	0	0	0	0	0	0	1	1	17.5	17.5	Respiratory	RE_468	Pneumonia
N50.8	0	0	1	0	0	0	0	0	0	1	17.4*	17.4*	Genitourinary	GU_608	Other disorders of male genital organs
L81.4	0	0	0	0	0	0	1	0	0	1	16.7	16.7	Dermatological	DE_674.2	Hyperpigmentation
I83	0	0	0	0	0	0	0	0	1	1	14.2	14.2	Cardiovascular	CV_444.11	Varicose veins of lower extremities
E83	1	0	0	1	0	0	0	1	0	3	5.0	13.0	Endocrine/Metabolic	EM_247	Disorders of mineral metabolism and mineral deficiencies
E83.1	1	1	0	1	1	0	0	1	1	6	2.5	12.3	Endocrine/Metabolic	EM_247.7	Disorders of iron metabolism
B01	0	0	0	0	0	0	0	0	1	1	11.9	11.9	Infections	ID_052.31	Varicella [chickenpox]
B01/B97.4/A85/A85.2	0	0	0	0	0	0	2	0	1	2	5.5	11.9	Infections	ID_089.2	Viral infections
E73.9	0	0	0	0	0	0	1	0	0	1	10.0	10.0	Endocrine/Metabolic	EM_252.51	Disorders of intestinal carbohydrate absorption
H65.1	0	0	0	0	0	1	0	0	0	1	9.1	9.1	Sense organs	SO_391.1	Otitis media
D03.8/C43.4/C43.0/D03	1	0	0	0	0	2	1	0	0	3	2.2	6.7	Neoplasms	CA_103.1	Melanomas of skin
C50.6/D03.8/C43.4/C16.6/C43.0/D03	1	0	1	0	0	2	1	0	0	4	1.7	6.7	Neoplasms	CA_130	Cancer (solid tumor, excluding BCC)
L98.4	0	0	0	0	0	0	1	0	0	1	6.7	6.7	Dermatological	DE_686.2	Non-pressure chronic ulcer
K86.1	0	0	1	0	0	0	0	0	0	1	6.0	6.0	Gastrointestinal	GI_554.12	Chronic pancreatitis
S92.0	0	0	1	0	0	0	0	0	0	1	3.5	3.5	Muscloskeletal	MS_745.4	Fracture of foot and toe

0, not significant in the group; 1, ICD-10 code aggregate present in either main or subcodes; 2, ICD-10 code aggregate present in both main and subcodes

*ICD-10* International Classification of Diseases, 10th Revision, *PheWAS* Phenome-wide association study, *CH* Compound heterozygote, *AH* Alternative homozygote, *BCC* Basal cell carcinoma

^*^Calculated for male carriers only, ↓ negative coefficient in the PheWAS

Three ICD-10 codes related to the urogenital tract were significant for p.S65C homozygotes: N01 (rapidly progressive nephritic syndrome), N42, and N34 (urethritis). For p.S65C homozygotes, the odds of N42 diagnosis were nearly 15 times higher than for the rest of the biobank participants and the odds of N34 diagnosis were nearly 8 times higher. Additionally, the odds of N50.8 (other disorders of male genital organs) diagnosis were about seven times higher for p.C282Y/p.S65C CHs than for the rest of the biobank participants.

Disorders of mineral metabolism (ICD-10 code E83) were significant in three genotype groups: p.C282Y homozygotes (13.0% of individuals diagnosed), all p.C282Y carriers (0.74%), and all CHs/AHs (1.5%). The code E83.1 was significant in six cohorts: p.C282Y homozygotes (12.3%), all p.C282Y carriers (0.42%), p.H63D homozygotes (0.44%), p.C282Y/H63D CHs (0.59%), all CHs/AHs (1.15%), and all carriers (0.93%). Neither E83 nor E83.1 reached significance in the p.S65C AH and CH groups.

The UKB PheWAS conducted with the same 14 genotype groups yielded 202 significant ICD-10 codes (61 main codes and 141 subcodes) after FDR-based correction (Additional File 1: Supplementary Figs. S29–S56). Besides E83 and D50, which are linked intrinsically to HH, I83 (varicose veins of lower extremities) was significant in the EstBB and UKB groups of all carriers and I10 was significant in both groups of all CHs/AHs. Additionally, J15 (bacterial pneumonia) was significant in the UKB group of ‘all carriers’, comparable to the significance of J18 in the EstBB group of ‘all carriers’. As in the EstBB analysis, this pneumonia-related ICD-10 code was significant only in the ‘all carriers’ group and not in the ‘all CHs/AHs’ group.

Notably, N29 and N29.8 (both for other disorders of kidney and ureter in diseases classified elsewhere) were significant in the p.S65C homozygote group, replicating the significance for ICD-10 codes related to the urogenital tract among p.S65C homozygotes in the EstBB analysis. As these codes were recorded for only one p.S65C homozygote in the UKB cohort, caution should be exercised when interpreting this result, but it does support the observed link between p.S65C homozygosity and symptoms of the urogenital tract.

**Fig. 4:**
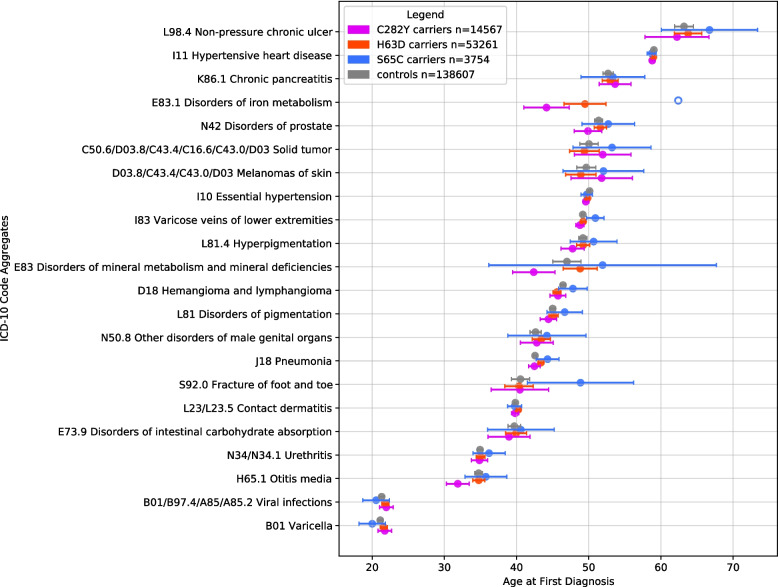
Mean ages at first diagnosis of most-frequent ICD-10 code aggregates in variant carriers and controls. The mean ages are represented as circles with 95% confidence intervals (whiskers). Groups with fewer than 10 individuals are represented as unfilled circles with no confidence interval. ICD-10 code aggregates are ranked in descending order of mean age at first diagnosis across all carriers. ICD-10, International Classification of Diseases, 10th Revision

**Fig. 5:**
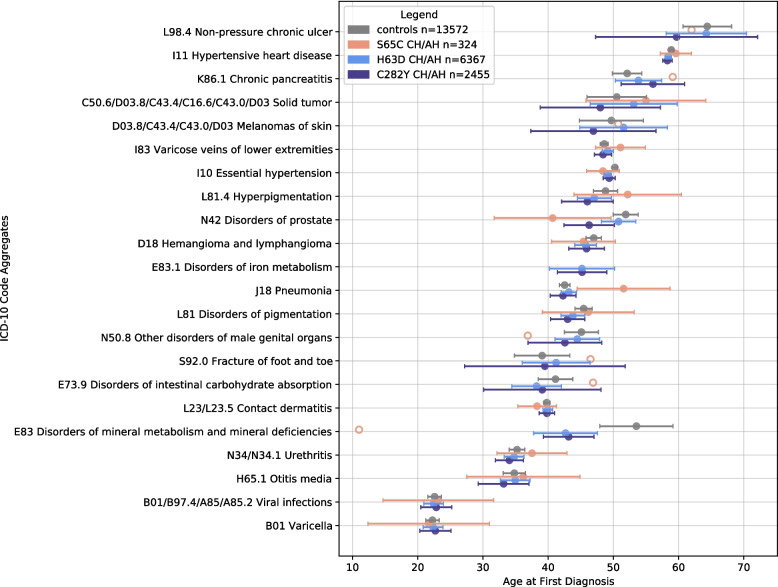
Mean ages at first diagnosis of most-frequent ICD-10 code aggregates in CHs/AHs and controls. The mean ages are represented as circles with 95% confidence intervals (whiskers). Groups with fewer than 10 individuals are represented as unfilled circles with no confidence interval. ICD-10 code aggregates are ranked in descending order of mean age at first diagnosis across all carriers. ICD-10, International Classification of Diseases, 10th Revision; CH, compound heterozygote; AH, alternative homozygote

The Kruskal–Wallis test was applied to the 25 most frequent unique ICD-10 code aggregates to detect codes that were both significantly more frequent (as determined by the PheWAS) and associated with significant differences in the age at first diagnosis among the study groups—controls vs. all p.C282Y, p.H63D, and p.S65C carriers (Fig. 4; Additional File 1: Supplementary Fig. S1) and controls vs. CHs/AHs for each variant (Fig. 5; Additional File 1: Supplementary Fig. S2). The Mann–Whitney *U* test was subsequently used to detect specific groups with differences in the age at first diagnosis using the codes identified as significant in the Kruskal–Wallis analysis. The control group for both CH/AH analyses (*n* = 13,572) was limited to controls matched to CH/AH variant carriers.

The Kruskal–Wallis test for p.C282Y, p.H63D, and p.S65C carriers in general yielded significant results for J18 Pneumonia (FDR-adjusted *p* = 0.0282). D18 Hemangioma and lymphoma (*p* = 0.046), I10 Essential hypertension (*p* = 0.018), I83 Varicose veins of lower extremities (*p* = 0.014), and H65.1 Otitis media (*p* = 0.009) showed nominal significance, but did not remain significant after multiple testing correction (Additional File 5: Table S4).

The ages at first diagnosis of N42 Disorders of prostate (FDR-adjusted *p* = 0.0498) and I10 Essential hypertension (FDR-adjusted *p* = 0.0498) differed significantly between the CH/AH group for each variant and controls before and after multiple testing correction. The Mann–Whitney *U* test revealed significant differences in the age at first N42 diagnosis between p.S65C CHs/AHs (41 years) and controls (52 years), p.C282Y CHs/AHs (46 years) and controls, and p.H63D CHs/AHs (51 years) and p.S65C CHs/AHs (Additional File 5: Table S4).

### EstBB GWAS yield novel ceruloplasmin and ferritin loci

To identify potential genetic modifiers of the HH phenotype, two GWAS were performed using quantitative trait measurements: ceruloplasmin (*n* = 4,964) and ferritin (*n* = 49,909) levels. Seven unique genomic risk loci and 21 lead single nucleotide polymorphisms (SNPs), as defined by FUMA, were identified in the GWAS based on the ferritin level. Two risk loci with four lead SNPs were identified in the GWAS based on ceruloplasmin measurements (Table 3, Additional File 1: Supplementary Material S1).

**Table 3:** Lead SNPs identified by FUMA in the EstBB GWAS

Trait	Chr	SNP	Position (GRCh37)	Nearest gene	Location relative to the nearest gene	Non-effect allele	Effect allele	Effect allele frequency	CADD	Beta	*P*-value	GWAS catalog	Comments
ceruloplasmin	3	rs10935742	148,900,861	CP	intronic	A	C	0.072	4.9	0.24	3.934E-11	yes	
ceruloplasmin	3	rs61733458	148,916,215	CP	exonic	A	G	0.031	26.8	−0.47	1.814E-13	no	
ceruloplasmin	3	rs34004251	148,929,951	CP	intronic	T	A	0.172	1.9	0.14	1.352E-09	yes	
ceruloplasmin	13	rs190025003	53,268,526	SUGT1	UTR3 region	A	T	0.031	1.1	−0.62	3.764E-11	no	in complete LD (*r*^2^ = 1) with *ATP7B* rs76151636
ferritin	1	rs1894692	169,467,654	AL021068.1	intergenic	G	A	0.011	0.7	−0.18	1.833E-19	yes	*r*^2^ > 0.9 with *F5* rs6025
ferritin	2	rs6434347	190,414,293	SLC40A1	intergenic	G	C	0.116	7.6	−0.05	1.326E-10	yes	
ferritin	12	rs4471501	51,425,352	SLC11A2	intergenic	T	C	0.251	10.8	−0.04	1.454E-08	yes	
ferritin	14	rs996347	34,410,892	EGLN3	intergenic	T	C	0.348	1.6	0.03	4.522E-09	yes	
ferritin	15	rs4775744	45,385,496	DUOX2	UTR3 region	A	T	0.076	1.4	−0.07	4.966E-15	no	
ferritin	15	rs190583525	96,497,945	RP11-4G2.1	intergenic	G	C	0.002	0.2	−0.56	3.16E-08	no	
ferritin	17	rs75912918	55,694,141	MSI2	intronic	G	A	0.011	18.3	−0.14	6.242E-09	no	
ferritin	17	rs184867000	55,922,229	MRPS23	intronic	C	G	0.001	13.4	−0.30	5.805E-27	no	
ferritin	17	rs62081796	56,098,211	SRSF1	intergenic	T	C	0.052	6.6	−0.07	5.365E-11	no	
ferritin	17	rs8178331	56,326,759	LPO	intronic	G	A	0.057	9.4	−0.07	1.881E-12	no	RDB 2b
ferritin	17	rs199598395	56,436,130	BZRAP1-AS1:RNF43*	exonic	C	T	0.002	24.7	−0.41	2.866E-53	yes	
ferritin	17	rs2257205	56,448,297	BZRAP1-AS1:RNF43*	exonic	C	T	0.116	24.5	0.04	8.463E-09	yes	
ferritin	17	rs3837804	56,476,781	BZRAP1-AS1:RNF43*	ncRNA_intronic	AGAGTCTTAGAGACTTGC	A	0.181	13.4	−0.05	3.429E-17	yes	
ferritin	17	rs73323127	57,362,386	SNRPGP17	intergenic	C	T	0.041	0.3	−0.11	3.436E-19	no	
ferritin	17	rs35205300	57,398,065	YPEL2	intergenic	G	A	0.033	0.1	−0.07	1.248E-11	no	
ferritin	17	rs1451504	57,411,445	YPEL2	intergenic	C	T	0.354	1.2	0.04	6.072E-13	yes	
ferritin	17	rs192569291	57,779,710	PTRH2	intronic	A	G	0.008	5.3	−0.36	1.279E-44	yes	
ferritin	17	rs4622571	57,884,397	VMP1	intronic	T	A	0.287	0.6	−0.04	2.498E-10	yes	
ferritin	17	rs189906970	58,281,729	USP32	intronic	G	A	0.036	2.6	−0.07	4.164E-10	no	
ferritin	17	rs117474222	58,784,290	BCAS3	intronic	T	G	0.026	7.7	−0.12	4.638E-12	no	
ferritin	17	rs141585217	59,199,184	BCAS3	intronic	C	T	0.024	0.2	−0.16	2.898E-18	no	

*SNP* Single nucleotide polymorphism, *FUMA* Functional Mapping and Annotation of Genome-Wide Association Studies, *EstBB* Estonian Biobank, *GWAS* genome-wide association study, *Chr* Chromosome, *CADD* Combined annotation dependent deletion, *UTR* Untranslated region, *LD* Linkage disequilibrium, *RDB* Regulome DB

^*^Transcripts with missense variants belonging to *RNF43*

**Fig. 6:**
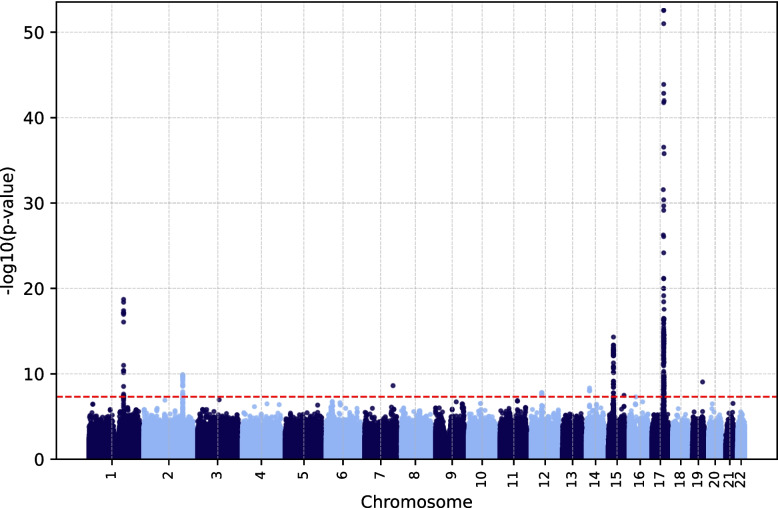
Manhattan plot of ferritin level–based EstBB GWAS results (*n* = 49,909). The red dashed line denotes the significance threshold (5 × 10^–8^). EstBB, Estonian Biobank; GWAS, genome-wide association study

The ferritin level–based study demonstrated the most polygenic architecture, as one identified locus each was on chromosomes 1, 2, 12, 14, and 17 and two identified loci were on chromosome 15 (Fig. 6). Ten of the 21 lead SNPs identified in the ferritin level–based GWAS have not been reported in the National Human Genome Research Institute–European Bioinformatics Institute GWAS catalog [37] (where Bell et al. [38] has been the largest contributor of ferritin GWAS associations to date); neither has any other significant SNP from their respective linkage disequilibrium (LD) blocks. ferritin Only three lead SNPs had positive beta coefficients reflecting increased ferritin levels.

**Fig. 7:**
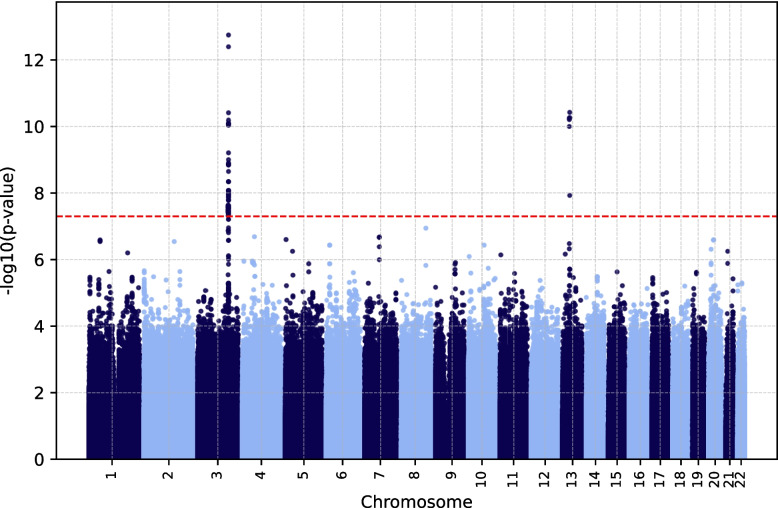
Manhattan plot of ceruloplasmin level–based EstBB GWAS results (*n* = 4,964). The red dashed line denotes the significance threshold (5 × 10^–8^). EstBB, Estonian Biobank; GWAS, genome-wide association study

The two novel genomic risk loci identified in the ceruloplasmin level–based GWAS were on chromosomes 3 and 13, with three lead SNPs and one lead SNP in the *CP* and *SUGT1* genes, respectively (Fig. 7). Three of the lead SNPs were intronic and one variant (rs61733458 in *CP*) was exonic, with a CADD score of 26.8.

### Fine-mapping of the EstBB GWAS results yields putatively causal SNP sets

Fine-mapping of the EstBB GWAS results was performed to identify sets of putatively causal SNPs (credible sets) and to estimate the causality of individual variants in these sets by calculating their posterior inclusion probabilities (PIPs; Additional File 6: Table S5). Fine-mapping of the ceruloplasmin level–based GWAS results indicated that the two loci on chromosomes 3 (6,756 variants tested) and 13 (5,367 variants tested) each had at least one 95% credible set. Two 95% credible sets were found at the chromosome 3 locus. In the set with the highest log10 Bayes factor (6.3), the *CP* variant rs61733458 was the SNP with the highest PIP (0.66). The credible set on chromosome 13 had a log10 Bayes factor > 5, and no individual variant stood out; the PIPs for all four variants were close to 0.25.

Fine-mapping of the ferritin level–based GWAS results revealed that seven loci [one each on chromosomes 1 (7,692 variants tested), 12 (4,742 variants tested), 14 (7,360 variants tested), and 15 (4,855 variants tested) and three on chromosome 17 (4,863, 3,155, and 3,301 variants tested, respectively)] had at least one 95% credible set. Two 95% credible sets were found at locus 17:56,779,710–58,779,710. An additional locus on chromosome 2 was tested, and no 95% credible set was detected. The variants with highest PIPs in the sets on chromosomes 1, 12, 14, and 15 matched the top lead SNPs identified at the genomic risk loci by FUMA. The variant with the highest PIP in the set at locus 17:55,436,130–57,436,130 also matched the top lead SNP identified on chromosome 17 by FUMA. Except for the second set at locus 17:56,779,710–58,779,710, all credible sets on chromosome 17 had very high log10 Bayes factor values (> 35) and strong negative effect sizes (–0.38 to –0.41).

### Meta-analyses of EstBB and UKB GWAS results

UKB ceruloplasmin and ferritin level–based GWAS results were entered into meta-analyses with the EstBB GWAS results to identify the most robust results across both cohorts (Additional File 7: Table S6). The concordance (consistent effect direction) of all variants was 29.8% (5,336,327/17,909,596) in the meta-analysis of the ceruloplasmin level–based results and 30.1% (8,731,966/29,059,175) in the meta-analysis of the ferritin level–based results. The meta-analysis of the ceruloplasmin level–based results yielded 897,307 variants with nominal significance (*p* < 0.05) and 564 variants with significance of *p* < 5 × 10^–8^. The meta-analysis of the ferritin level–based results yielded 1,534,748 nominally significant variants and 659 significant variants.

**Fig. 8:**
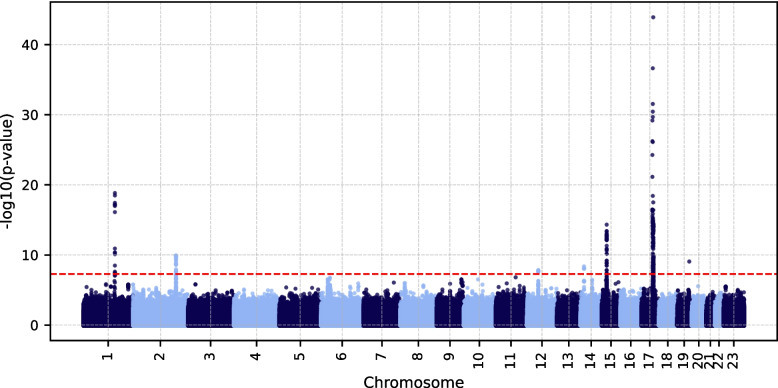
Manhattan plot of ferritin level–based EstBB and UKB meta-analysis results. Only concordant SNPs are displayed. The red dashed line denotes the significance threshold (5 × 10^–8^). EstBB, Estonian Biobank; UKB, UK Biobank; SNP, single nucleotide polymorphism

No concrete peaks were detected in the meta-analysis of the ceruloplasmin level–based results (Additional File 1: Supplementary Fig. S59). Six genomic risk loci were detected in the ferritin meta-analysis by FUMA: one each on chromosomes 1, 2, 12, 14, 15, and 17 (Fig. 8). Except for that on chromosome 15, the lead SNPs at all of these loci matched those identified by FUMA in the ferritin level–based EstBB GWAS. Three of these SNPs (*SLC19A2*/*F5* rs1894692, *SLC11A2* rs4471501, and *DUOX2* rs4775744) are not associated with iron metabolism in the GWAS catalog, nor is any other significant SNP in their respective LD blocks. The *SLC19A2*/*F5* variant rs1894692 (*r*^2^ > 0.9 with factor V Leiden variant rs6025) has previously documented associations with coagulation disorders (thrombosis) and cardiovascular disease in the GWAS catalog, suggesting that its impact on the ferritin level is likely tied more to inflammation than to iron overload.

## Discussion

This study was performed to investigate the expressivity of HH-associated variants and to identify potential genetic modifiers of the HH phenotype. We applied a combined genotype–phenotype approach to better understand the effects of pathogenic and potentially modifying HH-related genetic variants. Additionally, we examined the clinical penetrance of carrier and combination genotypes for the three most prevalent pathogenic *HFE* variants (p.C282Y, p.H63D, and p.S65C) using data from EHRs.

To our knowledge, we are the first to have performed a PheWAS for HH using data from two large population-based cohorts including p.C282Y, p.H63D, and p.S65C carriers and all combination genotypes. Our study includes the largest number of p.S65C carriers examined to date. Additionally, our study compares the efficacy of a pre-filtered approach of analyzing HH genotype–phenotype associations through common HH phenotypes as defined in literature and a hypothesis-free approach (PheWAS).

We found that the allele frequencies of *HFE* variants p.C282Y, p.H63D, and p.S65C in the EstBB cohort are similar to those in the FinnGen cohort [39] and in Latvia [40]. These frequencies revise the 2001 estimates for Estonia [41] slightly upward. The variant analysis of HH-associated genes indicated that all HH types not associated with *HFE* are likely to be very rare in Estonia, as is the case elsewhere [42]. We also found 3 novel potentially pathogenic HH variants (*SLC40A1* c.121 T > A, *HFE* c.737 T > G, and *TFR2* c.95G > T); unfortunately, the number of carriers was too low to draw meaningful conclusions about their phenotypic expressions.

Besides features with direct links to the pathophysiology of HH (high ferritin levels, iron and copper metabolism disorder diagnoses), no other clinical symptom category remained significant in the chi-squared analysis after multiple testing correction in this study. This finding may be attributable to the nonspecific nature of HH symptoms or the considerable variation in symptom category definitions among EHR-based HH studies, especially those relying on ICD-10 codes. This potential discrepancy highlights the need to apply hypothesis-free analytical methods (e.g., PheWAS) in the study of diseases with diverse symptom spectra such as HH.

High ferritin levels may be caused by conditions other than HH, such as inflammation and cancer [17]. Ideally, ferritin measurements would be accompanied by transferrin saturation measurements to determine their HH specificity. However, transferrin saturation is rarely recorded. The low percentages of E83.1 diagnoses obtained in this study may also be due to the median age of about 50 years in all groups, meaning that about half of individuals would not yet ordinarily display HH symptoms. If we assume that the younger half of the cohort will show the same pattern when older and double the rate of E83.1 diagnosis in the p.C282Y homozygote group (to 26.0%), our finding becomes comparable to the reported proportions of HH diagnoses in cohorts of participants older than 40 years [35, 36]. Following the same logic, the HH diagnosis rate remains below 2% for all other genotype groups in our sample.

One of the top significant ICD-10 code aggregates in our PheWAS was Neoplasms. This finding aligns with previous reports on HH and malignancies in various organ systems [43, 44]. The two most frequently recorded conditions in this category were hemangiomas and lymphangiomas (ICD-10 D18), and melanomas of the skin (ICD-10 D03.8/C43.4/C43.0/D03). The high frequency of contact dermatitis (ICD-10 L23/L23.5; affecting 42.2% of p.C282Y/p.S65C CHs) could be attributed in part to the misdiagnosis or co-occurrence of HH-related hyperpigmentation, which is recognized as the most common dermatological symptom of HH [45]. ICD-10 code aggregates representing pigmentation disorders (ICD-10 L81), and specifically hyperpigmentation (ICD-10 L81.4), were also significant among p.S65C homozygotes.

Generally, ferritin overload is considered to be an important step in the pathogenesis of HH. However, recent findings suggest that it does not account for all comorbidities associated with HH; for instance, p.C282Y homozygotes have been shown to be at greater risk of diabetes [46] and infection [47] even in the absence of iron overload, prompting questions about the biological effects of HH genotypes outside of the scope of the disease or preceding its onset. Similarly, we observed very low frequencies of HH diagnosis and ferritin elevation among p.S65C carriers in our sample, despite their high rate of skin hyperpigmentation. These low frequencies may instead reflect a lack of ferritin measurement in general, as these individuals are more likely to present with atypical HH and testing for this variant is not routinely performed.

The proportions of category prevalence in the EstBB PheWAS were largely identical for the groups ‘all variant carriers’ and ‘all CHs/AHs’, except for the addition of the Respiratory category for the ‘all variant carriers’ group. This pattern was replicated in UKB data, indicating that the signal is driven by the addition of simple heterozygotes to the sample. This signal is composed of the single ICD-10 code J18, which was diagnosed in 17.5% of all EstBB variant carriers. J18 was also identified as the only significant ICD-10 code aggregate in the comparisons of carriers of the three variants with controls using the Kruskal–Wallis test. Thus, even p.C282Y, p.H63D, and p.S65C simple heterozygotes could be more susceptible to the development of pneumonia, but there is little evidence to support this theory thus far [47].

Impotence and loss of libido are known symptoms of HH, with some studies placing either of these symptoms among those most commonly observed in HH patients [13, 14]. Although the four urogenital tract–related ICD-10 code aggregates in the Congenital category do not represent frequently occurring conditions (they affected 0.38–3.33% of individuals in the genotype groups), three of the most common code aggregates from the PheWAS belong to the Genitourinary category. Nearly 40% of male p.S65C homozygotes of all ages in the EstBB sample were diagnosed with disorders of the prostate (N42), and about 18% of male p.C282Y/p.S65C CHs were diagnosed with other disorders of the male genital organs (N50.8). A follow-up study revealed an association between p.C282Y homozygosity in males and prostate cancer [48], suggesting that p.C282Y promotes inflammation in the prostate; p.S65C carriers were not included in that study.

In addition to occurring more frequently in p.S65C homozygotes, the age at first N42 diagnosis differed significantly from that of controls in the p.S65C CH/AH (on average about 11 years earlier) and p.C282Y CH/AH (about 6 years earlier) groups. Although this category did not reach significance in chi-squared test, the prevalence of ICD-10 codes in the ‘Impotence and loss of sex drive’ category was nearly three-fold greater among p.S65C homozygotes than among controls (12.5% vs. 4.4%). Considering that sexual dysfunction may be one of the earliest HH manifestations and that nearly 50% of men with HH reported experiencing loss of libido prior to being diagnosed [49], our results indicate that there may be an unmet need for HH screening, including genetic testing for p.S65C, for middle-aged men presenting to sexual health services. Therefore, we would like to encourage future studies to evaluate the effectiveness of such an approach.

To our knowledge, results from only one previous ceruloplasmin level–based GWAS have been published [50]. That study was conducted with data from patients who had undergone coronary angioplasty and yielded one significant hit (rs13072552) in the *CP* gene [48]. Both independent GWAS hits on chromosome 13 in our study are in complete LD (*r*^2^ = 1) with rs76151636 in the *ATP7B* gene. This SNP has an allele (c.3207C > A) that is the most prevalent variant causing Wilson’s disease, a copper overload disorder characterized by low ceruloplasmin levels [51], in the European (and Estonian [52]) populations.

The exonic *CP* variant rs61733458 p.T551I has previously been found in patients with HH and the light iron phenotype [53], those with non-alcoholic fatty liver disease [54, 55], and those with Parkinson’s disease [56, 57]. The estimated effect size of rs61733458 (–0.47 g/L) was found to be nearly comparable to that of rs76151636, a SNP with a well-established impact on ceruloplasmin levels. This evidence, along with its identification as a top hit in our ceruloplasmin level–based GWAS, suggests that p.T551I plays a role in the development of iron metabolism–associated diseases by modulating the ceruloplasmin level and thereby inducing hyperferritinemia.

In our ferritin level–based EstBB GWAS, two missense variants with CADD score > 24 were identified in the *RNF34* gene on chromosome 17. The rare missense variant rs199598395 was reported in a study of iron deficiency anemia in Finnish individuals [58] and is recorded as occurring only in the Finnish population in GnomAD v4.1.0 [59]. It was similarly rare in the EstBB cohort and had a relatively large negative effect size. The second variant, rs2257205, is much more common and had a small positive effect size in the EstBB analysis. *RNF43* has been associated with serrated polyposis syndrome [60], indicating that its link to the ferritin level may be through hemorrhaging polyps. However, further studies are needed to determine the true effect of the gene, as there is little evidence so far.

The meta-analysis of the ceruloplasmin level–based GWAS results yielded largely inconclusive results, likely due to the insufficient size of the UKB sample. The low count of ceruloplasmin measurements in the UKB data is most likely attributable to the fact that the UKB laboratory measurement data derive solely from general practitioner (GP) surgeries (available for roughly 45% of the entire UKB cohort), whereas GPs do not routinely order ceruloplasmin measurement. This factor may also be partly responsible for the number of discordant peaks between the EstBB and UKB ferritin level–based GWAS results. Despite the issues outlined above, rs76151636 was significant (*p* = 5.7 × 10^–11^) and rs61733458 was nominally significant (*p* = 0.005) in the ceruloplasmin meta-analysis.

The low count of ceruloplasmin measurements in the UKB GWAS sample and the limited number of p.S65C homozygotes are the main limitations of this study. Using the combined power of two population-based biobanks, we analyzed data from > 15,800 p.S65C carriers. To our knowledge, this is the largest cohort of p.S65C carriers included in an analysis to date. As p.S65C homozygotes are rare, we recommend the analysis of pooled data from p.S65C homozygotes in several populations worldwide to validate our results pertaining specifically to this group. Additionally, future studies focusing on the functional characterization of the *CP* variant rs61733458—a lead hit with a significant effect size in the EstBB GWAS—would be valuable to elucidate the in vivo effects of this variant and clarify its clinical significance.

The purpose of performing the ferritin and ceruloplasmin GWAS in our study was to investigate the possibility of additional genetic factors in addition to known HH-associated variants that may influence the expression and penetrance of HH or other iron metabolism-linked disorders. Ideally, this study lays the groundwork for conducting future pilot studies of deep phenotyping for carriers of variants such as *CP* p.T555I or *RNF43* p.G336D. If found to be clinically relevant in addition to computational predictions of pathogenicity, we believe that these variants have the potential to become new diagnostic markers for predicting the expression of specific HH symptoms in the future.

Currently, testing in clinical settings in Estonia covers predominantly the p.C282Y and p.H63D variants. The updated 2025 guidelines published by Haemochromatosis UK [61] suggest that a wide variety of symptoms should be considered to be evocative of HH, and that p.S65C genotypes should be included in genetic testing for HH. Based on our results, obtained with linked genotype–phenotype biobank data, we consider these guidelines to be a step in the right direction and suggest that similar changes be made to current HH diagnosis guidelines in Estonia.

## Conclusions

Overall, our work showed significant differences in phenotypic expressivity and predominant symptom categories among the p.C282Y, p.H63D, and p.S65C genotypes. We assessed the spectra of pathogenic HH variants of all types in the Estonian population and found three novel variants. Our PheWAS revealed a previously unreported association between p.S65C homozygosity/compound heterozygosity in males and symptoms of the urogenital tract. Additionally, p.S65C carriers were found to have high rates of skin pigmentation disorders and, specifically, hyperpigmentation. We also identified a previously unreported *CP* variant (rs61733458); strong evidence in the literature links this variant to iron metabolism–related diseases and significantly decreased ceruloplasmin concentrations, suggesting that it influences the development of these phenotypes. In our view, the link between p.S65C homozygosity and urogenital tract disorders warrants further exploration, and HH diagnosis guidelines should be reviewed with consideration of the inclusion of the p.S65C variant in genetic testing for atypical HH cases.

## Supplementary Information

Supplementary Material 1.

Supplementary Material 2.

Supplementary Material 3.

Supplementary Material 4.

Supplementary Material 5.

Supplementary Material 6.

Supplementary Material 7.

## Data Availability

The genotypic and phenotypic data from the Estonian Biobank are available under restricted access (application process described in detail at https://genomics.ut.ee/en/content/estonian-biobank#dataaccess) and can be obtained with the permission of the Estonian Committee on Bioethics and Human Research. The UK Biobank data used in this study are available under restricted access, which can be requested through a standard protocol (https://www.ukbiobank.ac.uk/enable-your-research/apply-for-access) via the UK Biobank Access Management System.

## Abbreviations

AH: Alternative homozygote
CADD: Combined annotation dependent deletion
CH: Compound heterozygote
EHRs: Electronic health records
EstBB: Estonian Biobank
FDR: False discovery rate
FUMA: Functional Mapping and Annotation of Genome-Wide Association Studies
GP: General practitioner
GWAS: Genome-wide association study
HH: Hereditary hemochromatosis
ICD-10: International Classification of Diseases, 10th Revision
LD: Linkage disequilibrium
MAF: Minor allele frequency
PheWAS: Phenome-wide association study
PIP: Posterior inclusion probability
SNP: Single nucleotide polymorphism
UKB: UK Biobank
UK BiLEVE: UK Biobank Lung Exome Variant Evaluation
WES: Whole-exome sequencing
WGS: Whole genome sequencing
