# Supplementary material for: Unraveling genotype–phenotype relationships in hereditary hemochromatosis through integrated biobank data analysis
Source: BMC Genomics. 2026 Mar 16;27:405. doi: 10.1186/s12864-026-12746-3 (PMC13104239; doi:10.1186/s12864-026-12746-3)

Supplementary Material S1

Table of Contents

[Additional Information 1](#_Toc202883195)

[Supplementary Figs. S1 and S2. Boxplots of ages at first diagnosis for the most frequent ICD-10 code aggregates. 4](#_Toc202883196)

[Supplementary Figs. S3–S28. Manhattan plots of EstBB PheWAS results. 8](#_Toc202883197)

[Supplementary Figs. S29–S56. Manhattan plots of UKB PheWAS results. 22](#_Toc202883198)

[Supplementary Figs. S57–S59. Manhattan plots of UKB GWAS meta-analysis results. 36](#_Toc202883199)

# Additional Information

**Preprocessing of laboratory measurements from electronic health records**

All laboratory measurements were retrieved from linked electronic health records [Estonian Biobank (EstBB) data freeze 2024v01] containing data from major hospital and general practitioner practices in Estonia. The measurements were subjected to quality control, which consisted of the removal of duplicate records, blank measurement values, records with incorrect units, and logical observation identifiers names and codes (LOINC).

**Alanine aminotransferase and aspartate aminotransferase levels**

All measurements labeled as alanine aminotransferase (ALT) or aspartate aminotransferase (AST; *n* = 1,951,355) were retrieved. After quality control, 985,697 entries remained. ALT/AST values ≥ 35 U/L for females and ≥50 U/L for males were defined as above the reference. Elevated liver enzyme activity for >6 months was defined by at least two ALT/AST measurements above the reference dated at least 6 months apart.

**Ceruloplasmin level**

All measurements labeled as ceruloplasmin (*n* = 9,314) were retrieved. Sixty-six entries were converted from mg/L to standard g/L units. The processed ceruloplasmin dataset contained 6,184 entries corresponding to 5,139 unique individuals. Ceruloplasmin values < 0.16 g/L for females and <0.15 g/L for males were considered to be below the reference.

**Ferritin**

All measurements labeled as ferritin (*n* = 120,584) were retrieved. After quality control, 118,526 entries corresponding to 51,164 unique individuals remained. Ferritin levels ≥ 150 g/L for females and ≥400 g/L for males were considered to be above the reference.

**Glycosylated hemoglobin**

All measurements labeled as glycosylated hemoglobin (HbA1c; *n* = 489,405) were retrieved. A maximum HbA1c value of 25% was set to exclude potentially falsely labeled entries. HbA1c values ≥ 5.9% were defined as above the reference.

**REGENIE genome-wide association studies**

**Input file generation**

For quantitative traits, average values across all measurements available for each individual were entered into phenotype columns for REGENIE [28] analysis. The top 10 genotype principal components for each individual in the EstBB cohort were extracted from a previously computed file. Only participants with data on age, sex, EstBB identifiers, and genotype principal components were included. One individual from each monozygotic twin pair was removed at random. EstBB and UK Biobank (UKB) genome-wide association studies (GWAS) were run on human genome reference build GRCh37 (hg19).

**Ceruloplasmin level–based GWAS**

The quantitative ceruloplasmin level–based GWAS was conducted with data from 4,964 EstBB participants. Two genomic risk loci were identified: 3:148700328-148943264 and 13:52223552-54089493. Four lead single nucleotide polymorphisms (SNPs) and 133 candidate SNPs were identified across 13 mapped genes.

**Ferritin level–based GWAS**

The quantitative ferritin level–based GWAS was conducted with data from 49,909 EstBB participants. Seven genomic risk loci were identified: 1:168509761-169521553, 2:190362758-190441066, 12:51328269-51534886, 14:34408083-34434633, 15:45365790-45415487, 15:96497945-96497945, and 17:55694141-59201645. Twenty-one lead SNPs and 1,069 candidate SNPs were identified across 82 mapped genes.

**Matching cases and controls**

Case–control matching was performed based on sex, age, and BMI. We used a custom C++–based optimization script to select three controls (two in the case of the chi-squared test) for each case. The algorithm required an exact match on sex and minimized differences in age and BMI by leveraging information from the full pool of eligible controls. As a result, typically about 94-95% of age matches were exact, the mean age difference between cases and controls differed by less than 0.1 years, and the mean BMI differed less than 0.05. Before matching, 299 individuals with iron-metabolism disorders were removed from the pool of potential controls.

**Post-GWAS analyses**

GWAS signals were annotated using the Functional Mapping and Annotation of Genome-Wide Association Studies platform (v1.6.1) [29]. Default values were used for the lead SNP *p-*value threshold (<5 × 10^-8^), *r* ^2^ threshold for independently significant SNPs (≥0.6), and maximum distance of linkage disequilibrium blocks for merging into single genomic loci (≤250 kb). The 1000G Phase3 EUR reference panel was used to compute *r*^2^ values and minor allele frequencies (MAFs).

Fixed-effects meta-analyses of the EstBB and UKB GWAS results were performed using METAL (version 2020-05-05) [30]. Summary statistics files were first harmonized by aligning alleles and effect directions between datasets using a custom pipeline implemented in Unix shell and Python scripts. METAL was configured to use the effect size and standard error scheme, with markers identified by SNP IDs and genomic positions. The TRACKPOSITIONS ON option was employed to prioritize matching variants by genomic coordinates when SNP IDs differed. Phenotype data from the UKB GWAS were pre-processed using the procedure detailed above for the EstBB GWAS.

The fine-mapping of EstBB GWAS variants was performed using SuSIE (v0.14.2) [31] and LDstore2 (v2.0) [32] with a pipeline specifically adapted for EstBB data (v0.2) [33]. Default pipeline values were used for the candidate SNP *p*-value threshold (<5 × 10^-8^), MAF (>0.01), information score (>0.4), genomic window size (1,000,000 bp) and maximum number of causal SNPs (*n* = 10). The pipeline included only loci with ≥50 variants after filtering. Only pure credible sets (*r*^2^ > 0.5) were reported.

**Software versions and functions**

Numpy (v1.19.2), matplotlib (v3.3.2), and pandas (v1.1.3) were used in Python 3.8.5 throughout the analyses. The chi-squared test with adjusted residuals was performed in Python with chi2_contingency from scipy (v1.5.2). Fisher’s exact test, the Kruskal–Wallis test, and the Mann–Whitney *U* test were performed using fisher_exact, Kruskal, and mannwhitneyu, respectively, from scipy. multipletests and smm from statsmodels (v0.12.0) were used for multiple testing. The r-qdap/2.4.3, r-phewas/1.0, r-lattice/0.20-44, and r-dplyr/1.0.10 modules were used to run the custom in-house PheWAS script in R 4.1.3. REGENIE was used to perform the GWASs with the EstBB (v3.4) and UKB (v3.2.9) data. Matplotlib and seaborn (v0.11.0) were used to generate figures. A custom C++ script was used to find best global fits between cases and controls matched by age, sex, and body mass index.

# Supplementary Figs. S1 and S2. Boxplots of ages at first diagnosis for the most frequent ICD-10 code aggregates.

**Supplementary Fig. S1.** Boxplots of ages at first diagnosis for the most frequent ICD-10 code aggregates for all C282Y, H63D, and S65C carriers included in the PheWAS. ICD-10, International Classification of Diseases,10^th^ Revision; PheWAS, phenome-wide association study.


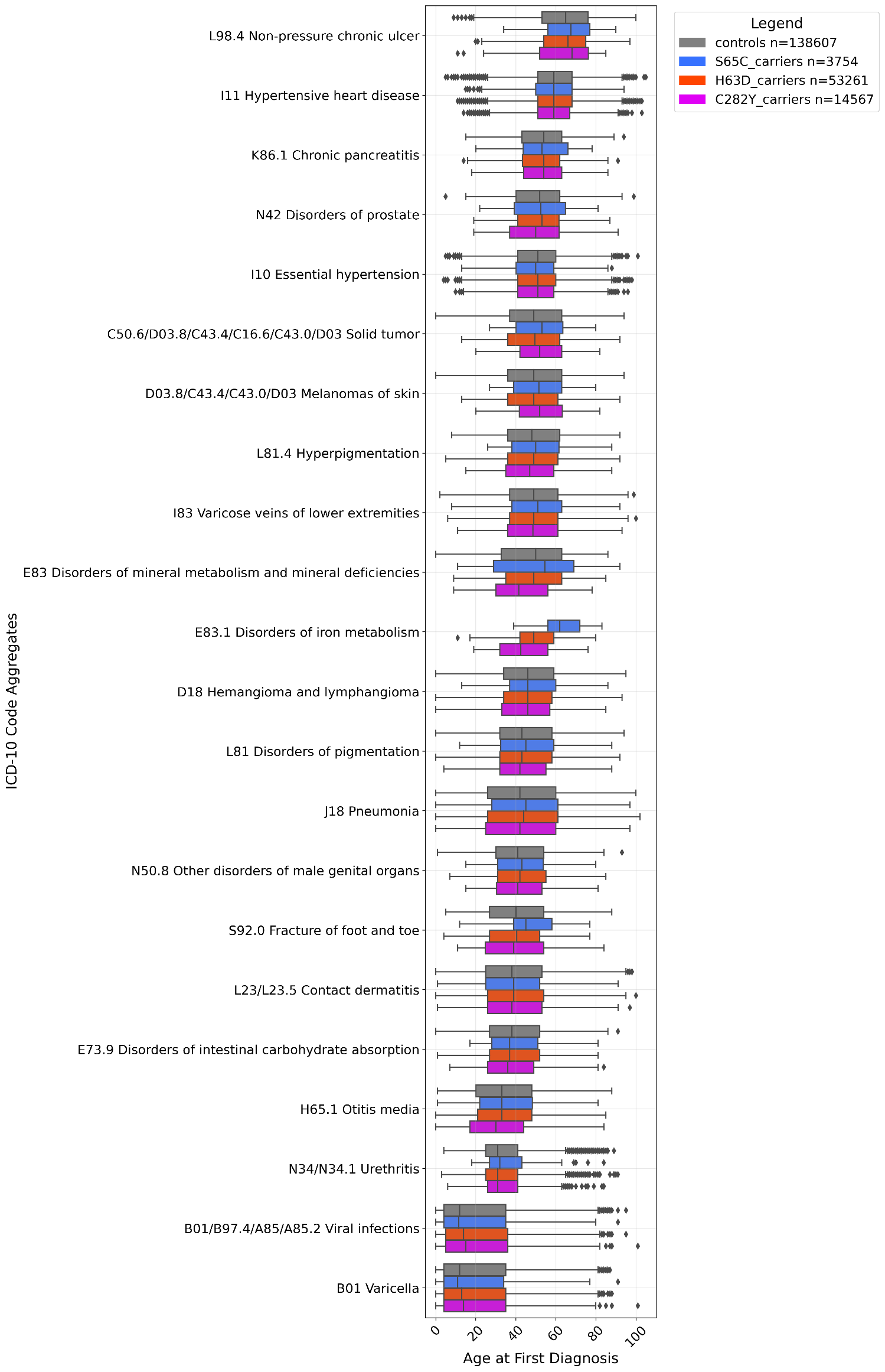


**Supplementary Fig. S2.** Boxplots of ages at first diagnosis for the most frequent ICD-10 code aggregates for C282Y, H63D, and S65C CHs/AHs included in the PheWAS. ICD-10, International Classification of Diseases, 10^th^ Revision; CH, compound heterozygote; AH, alternative homozygote; PheWAS, phenome-wide association study.


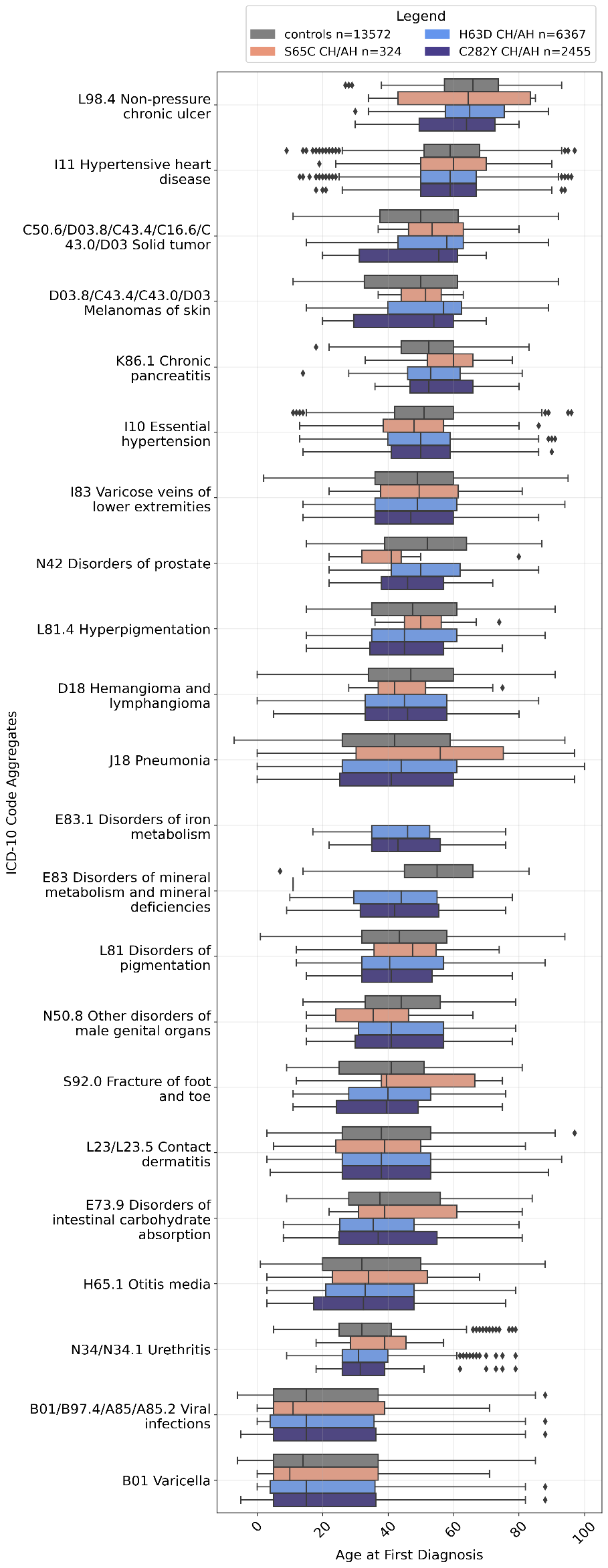


# Supplementary Figs. S3–S28. Manhattan plots of EstBB PheWAS results.

**Supplementary Figs. S3–S28.** Manhattan plots of EstBB PheWAS results. The red lines demarcate the threshold of FDR-adjusted *p* < 0.05. Upward-facing markers represent positive effect sizes and downward-facing markers represent negative effect sizes. EstBB, Estonian Biobank; PheWAS, phenome-wide association study; FDR, false discovery rate; CH, compound heterozygote; AH, alternative homozygote.


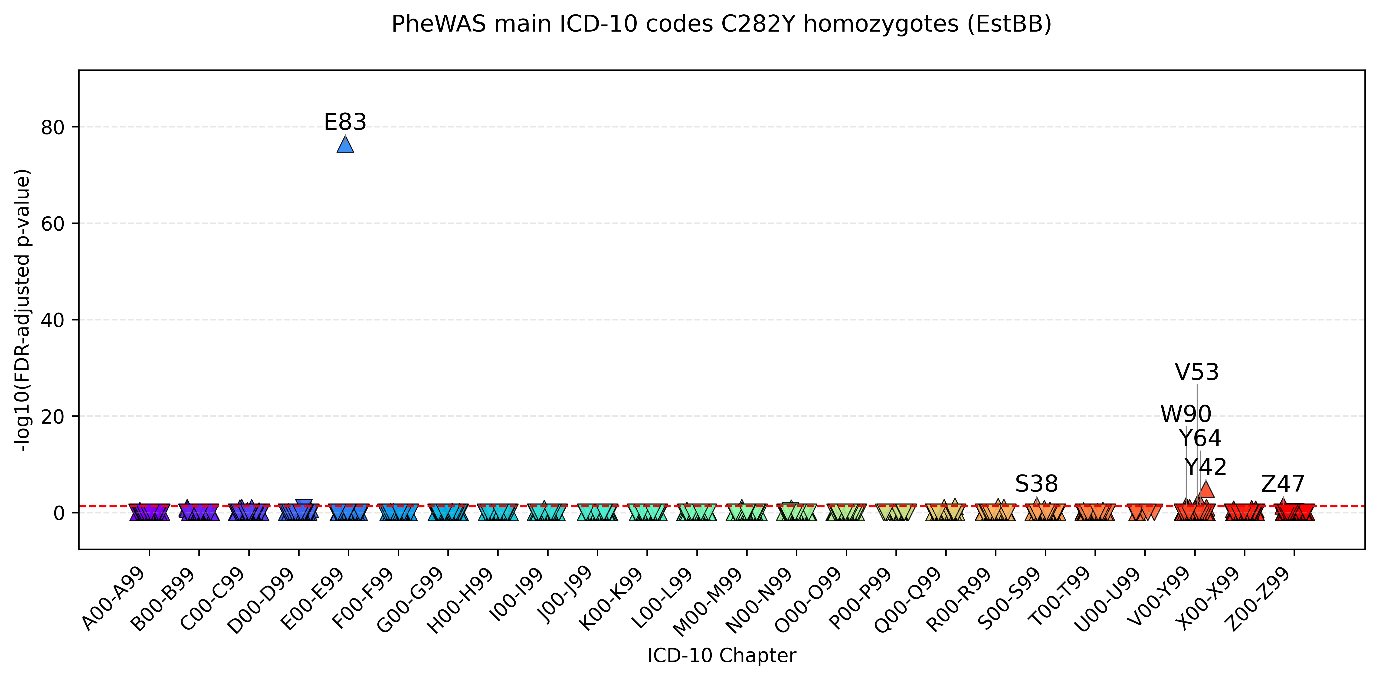


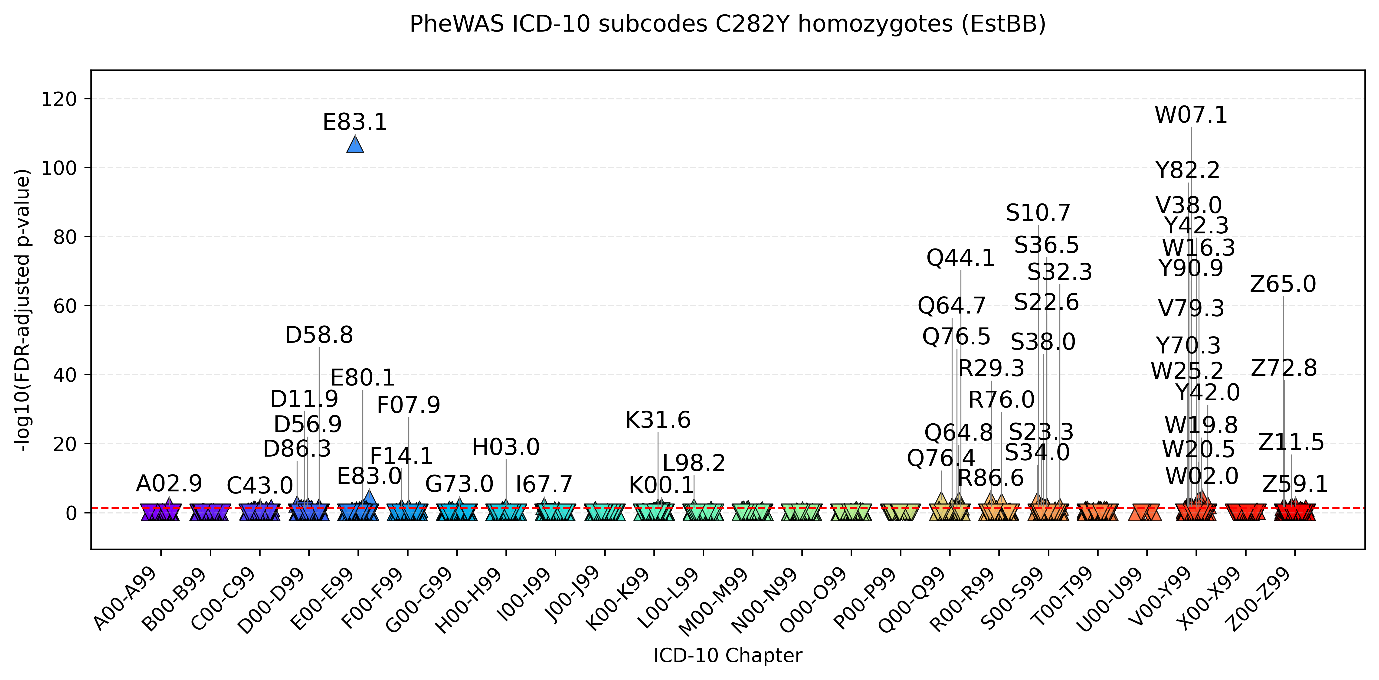


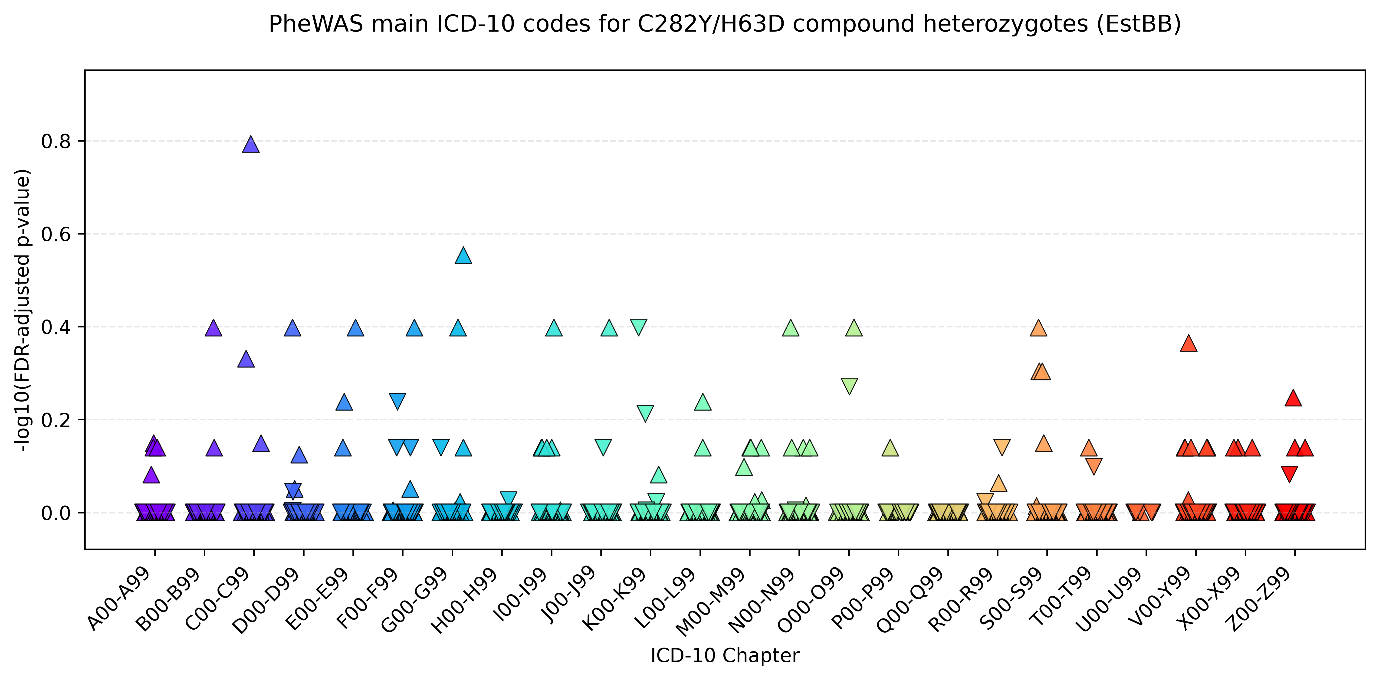


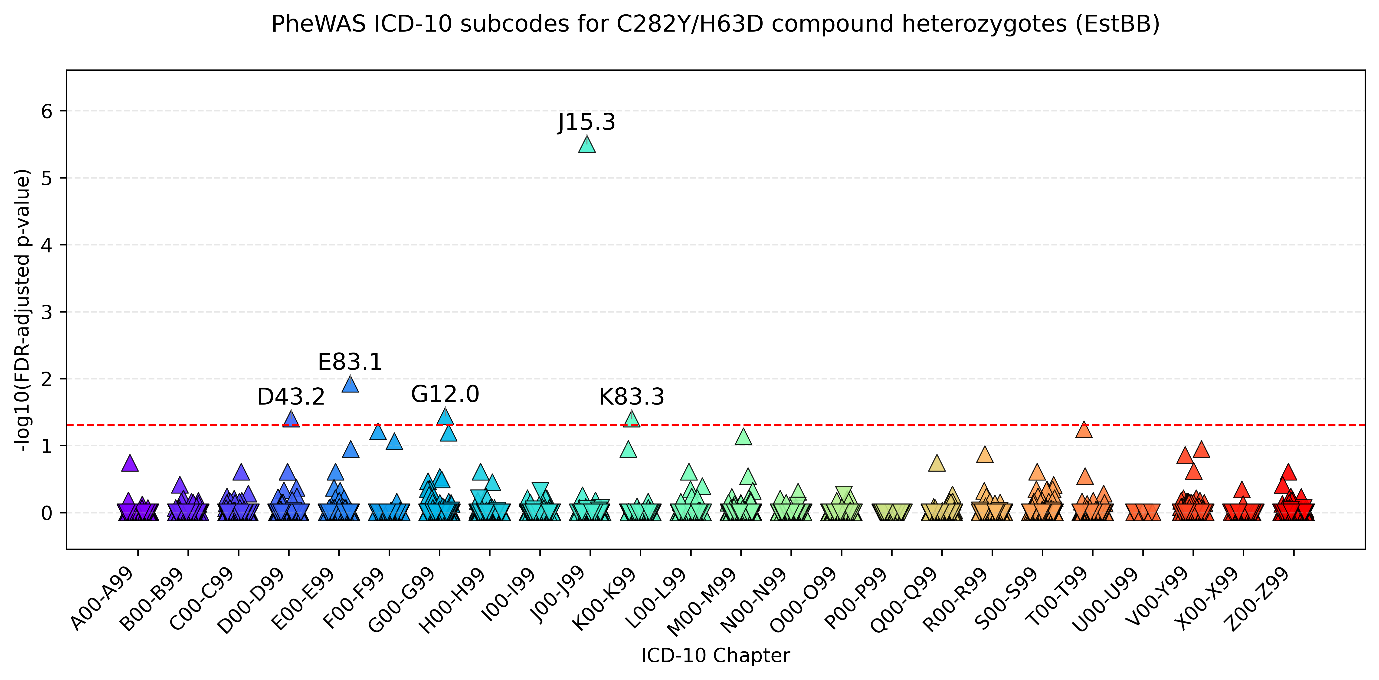


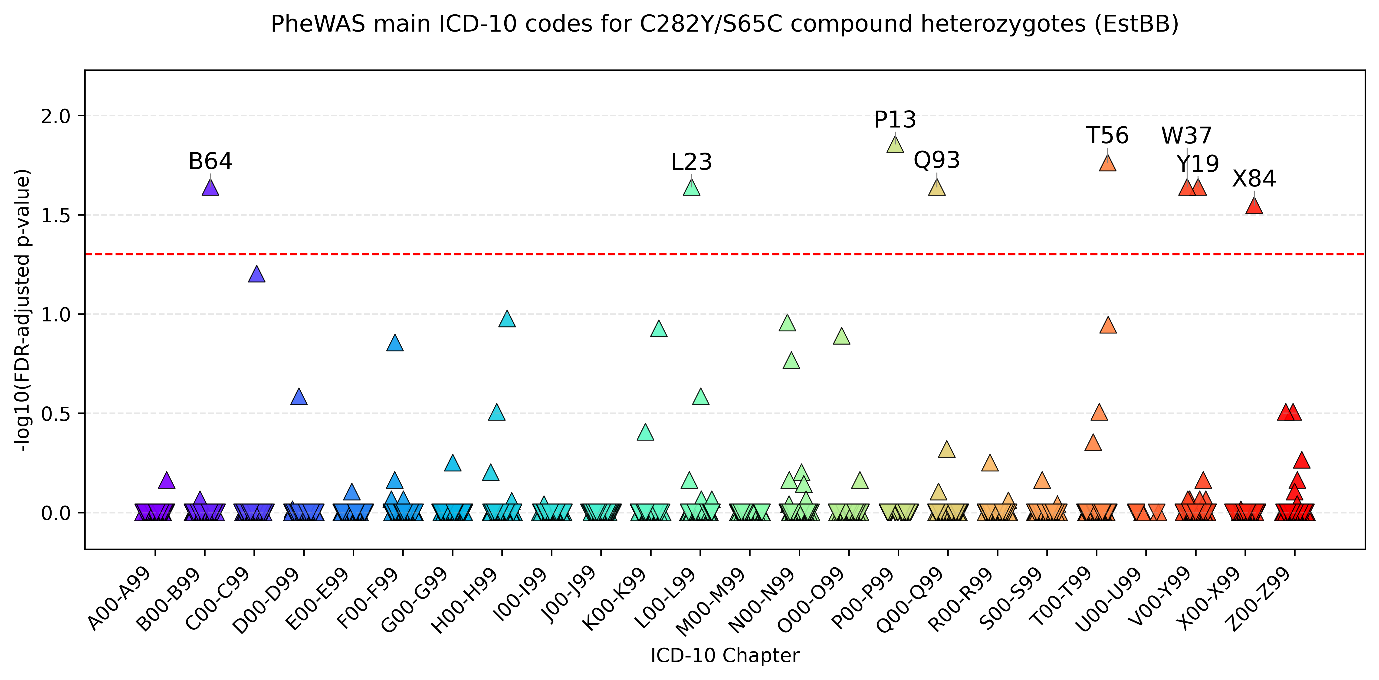


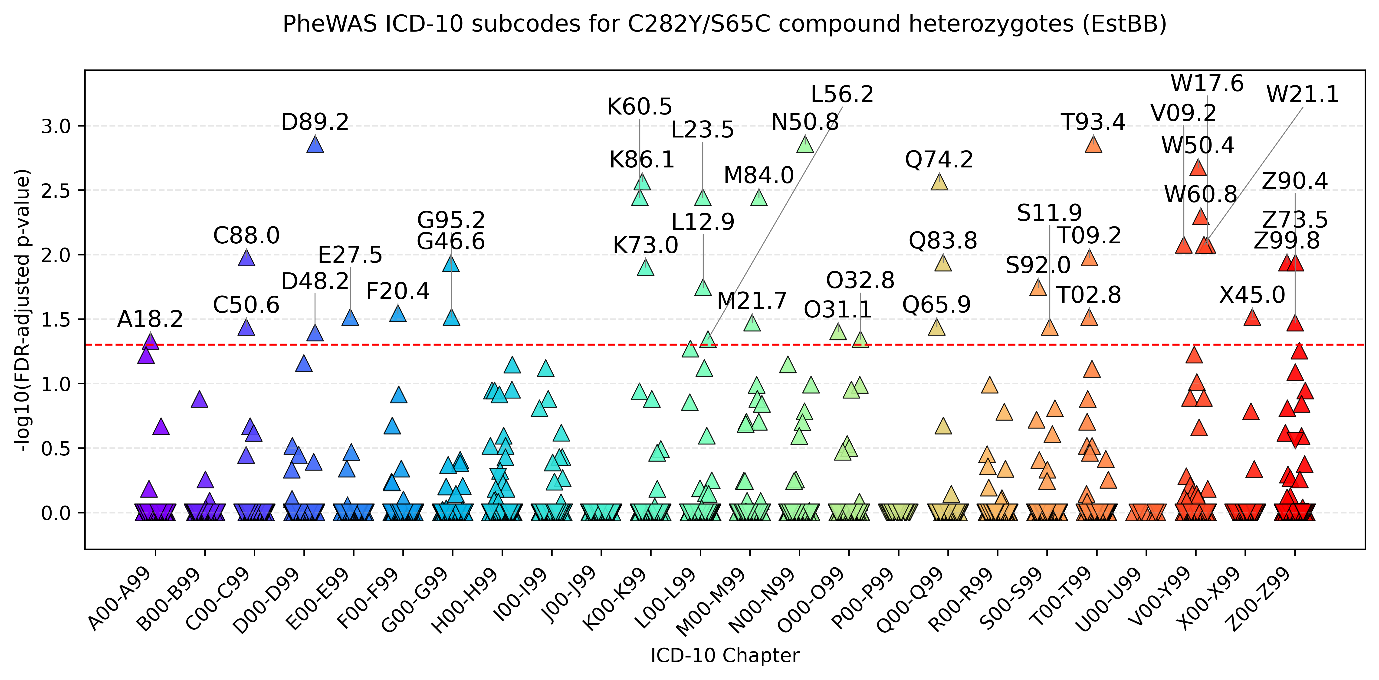


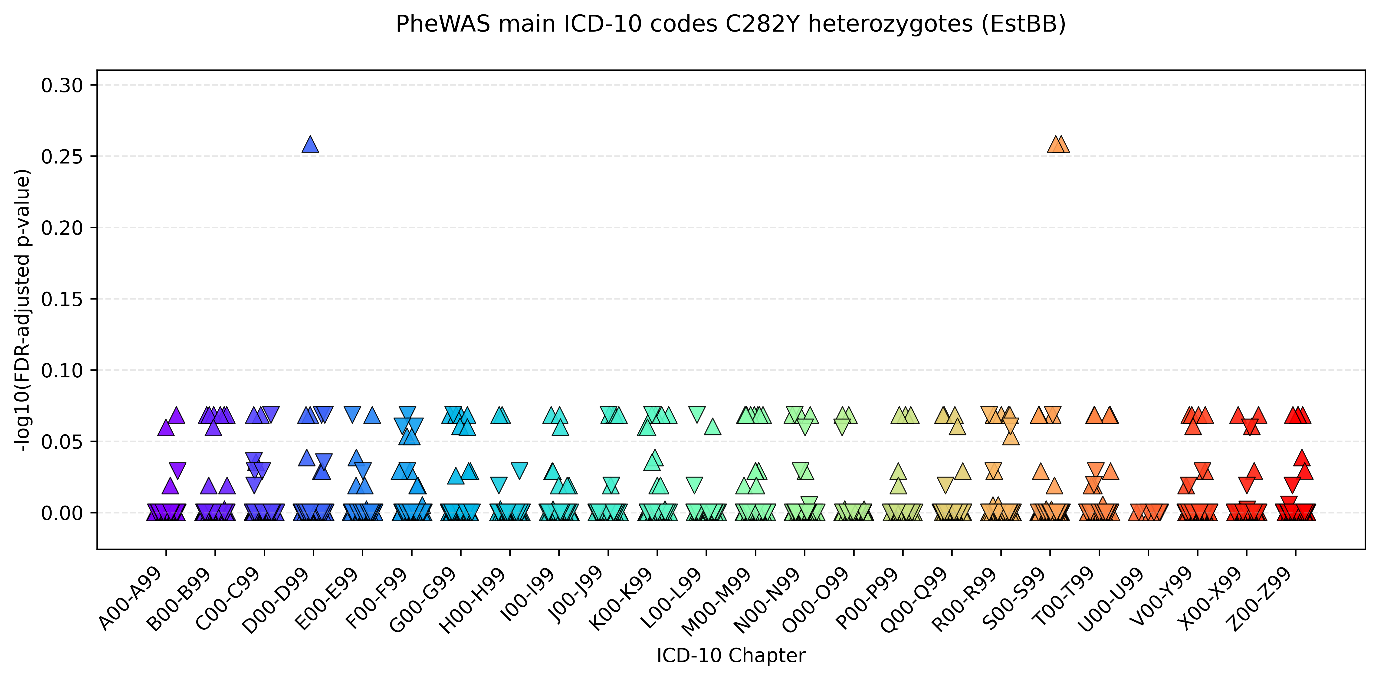


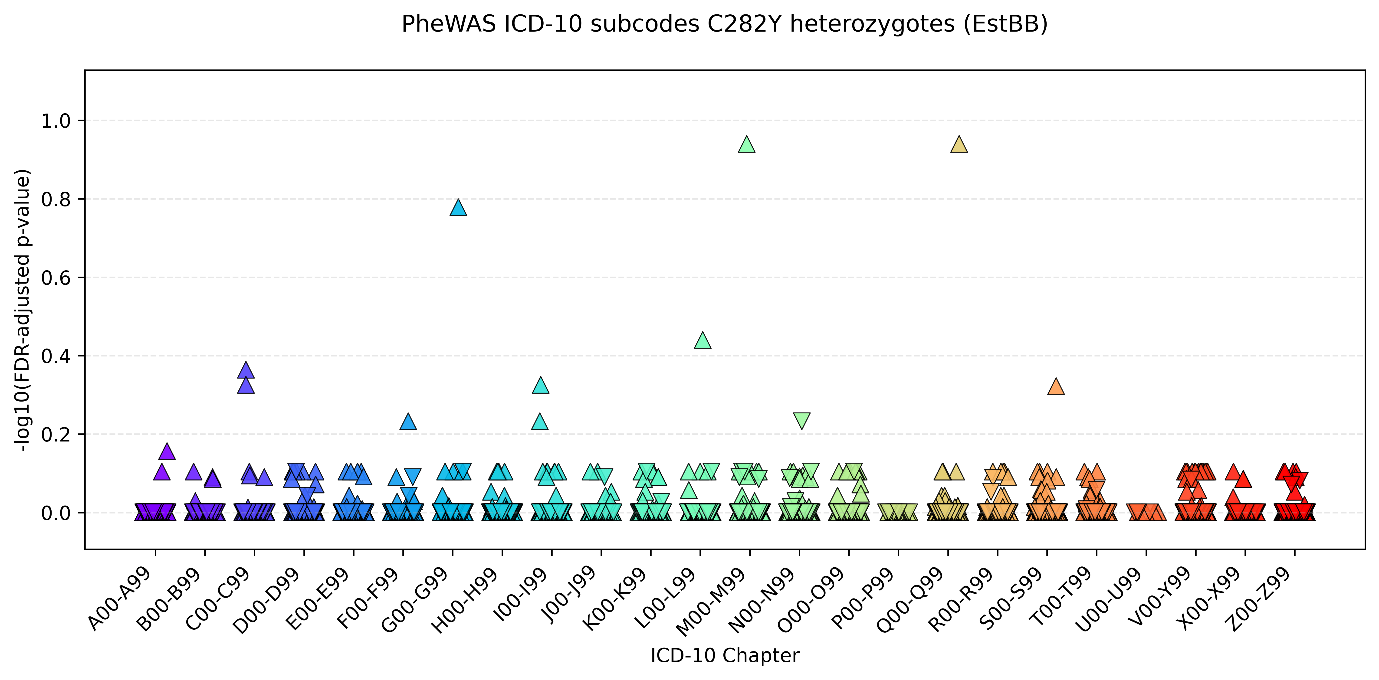


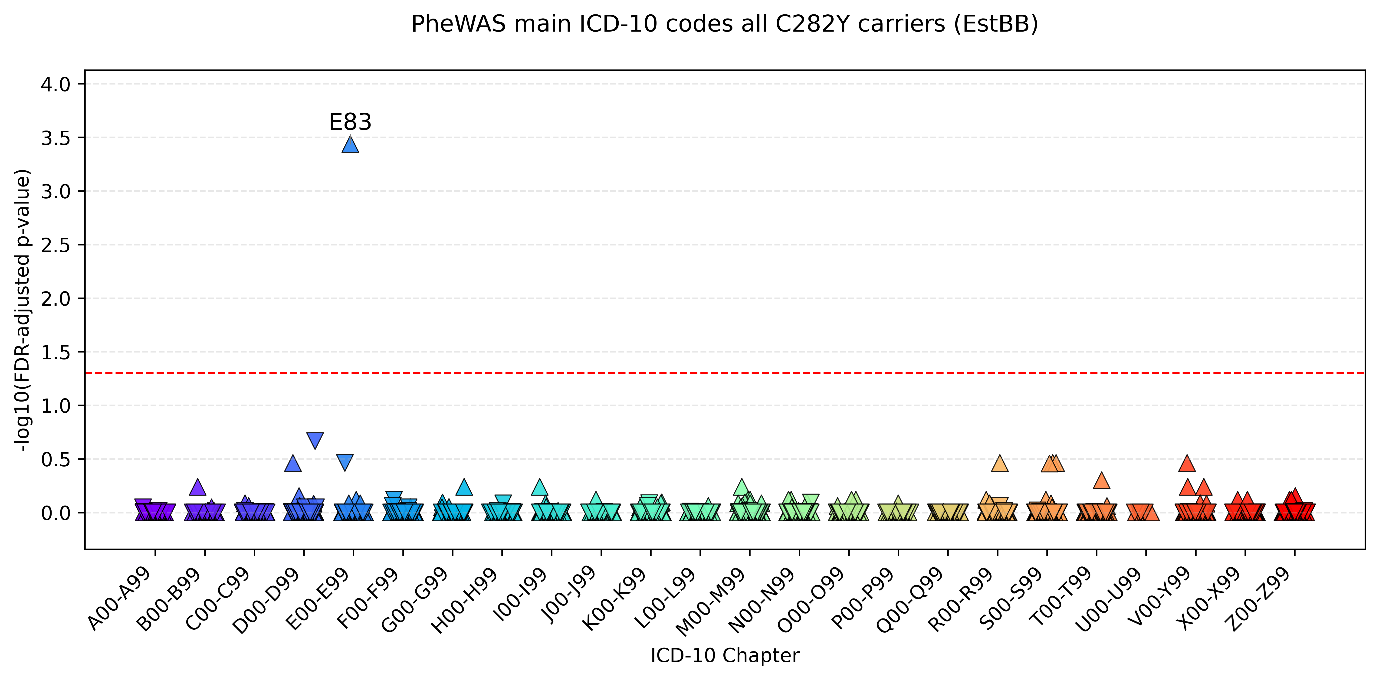

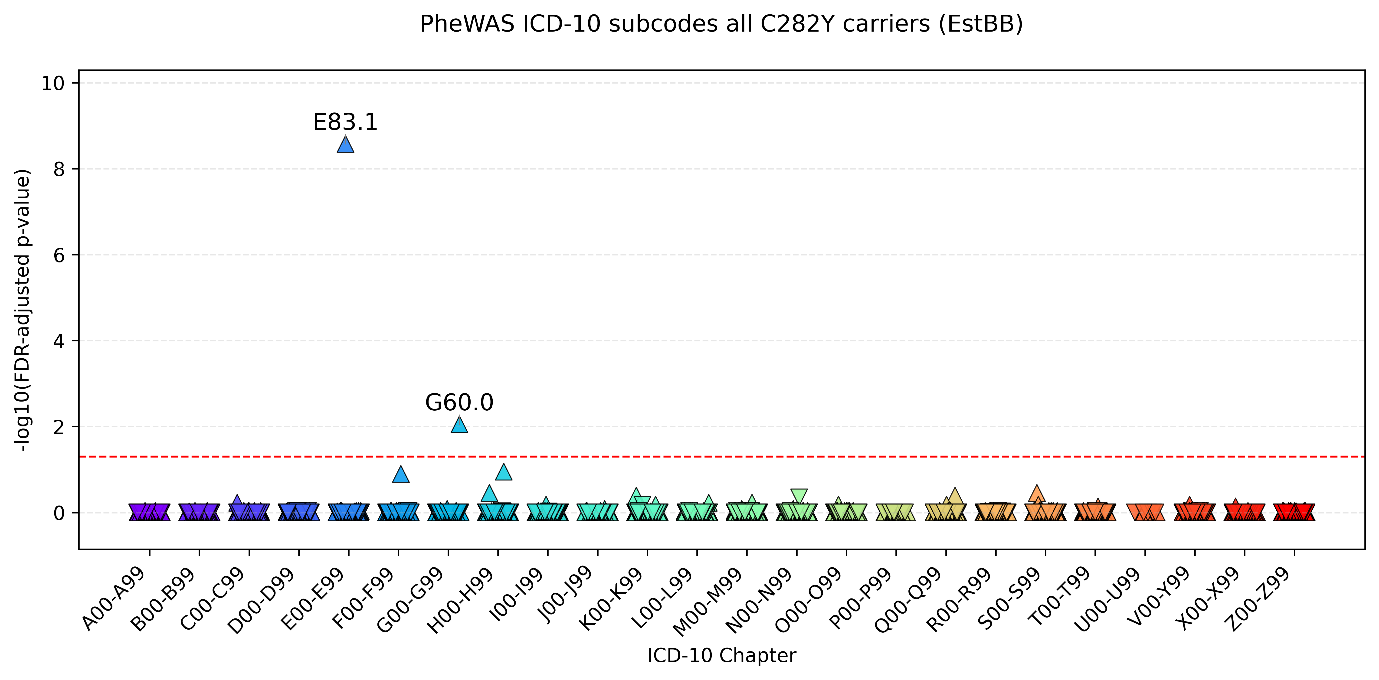


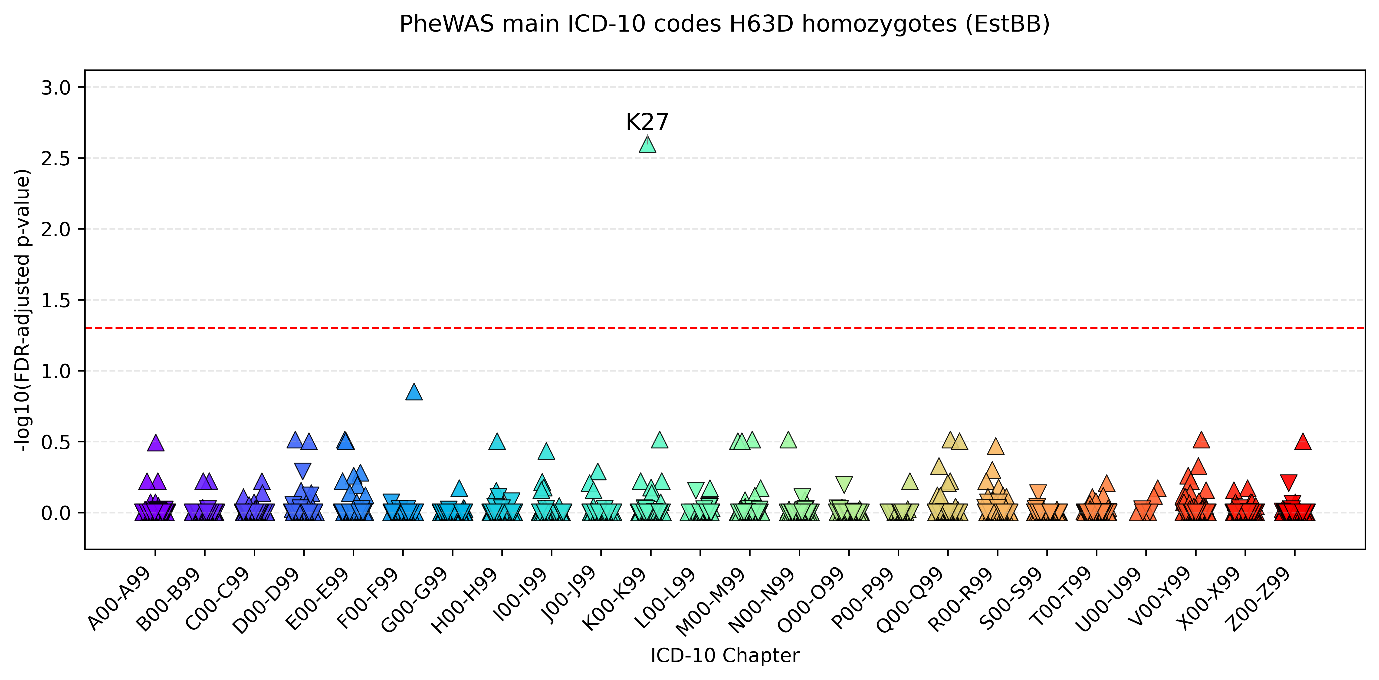


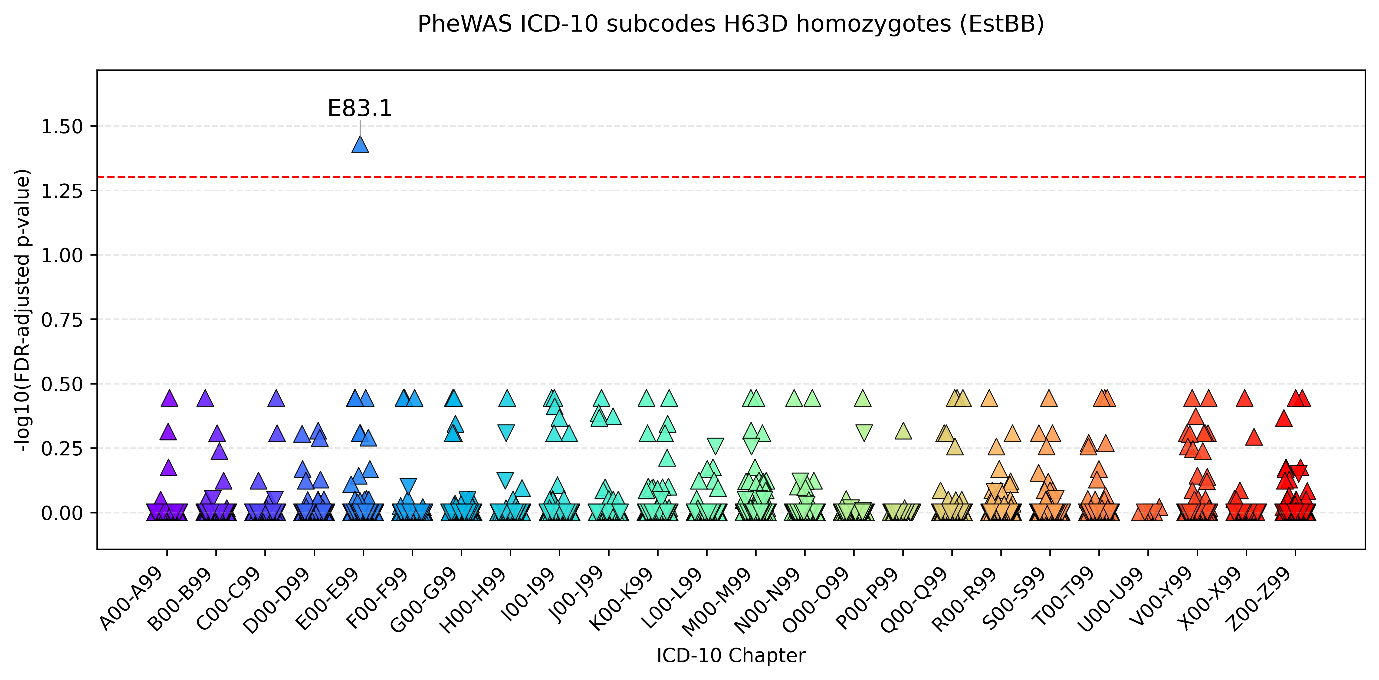


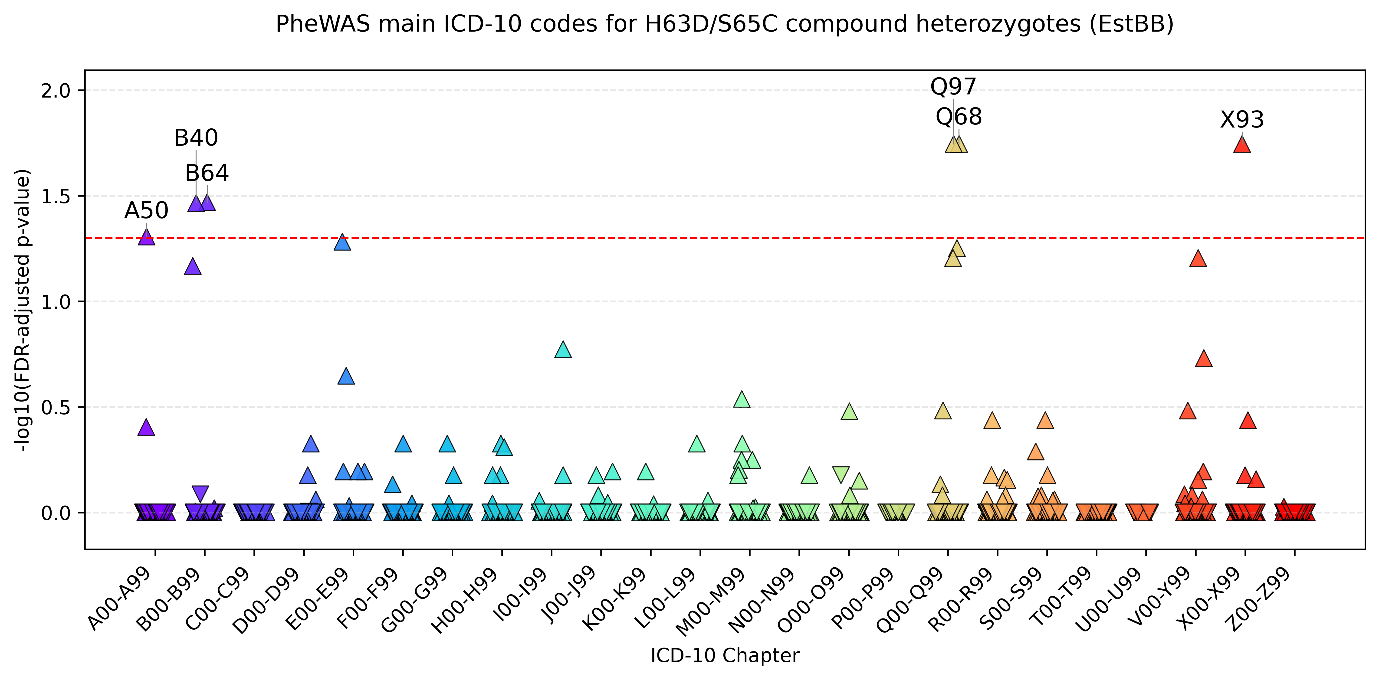


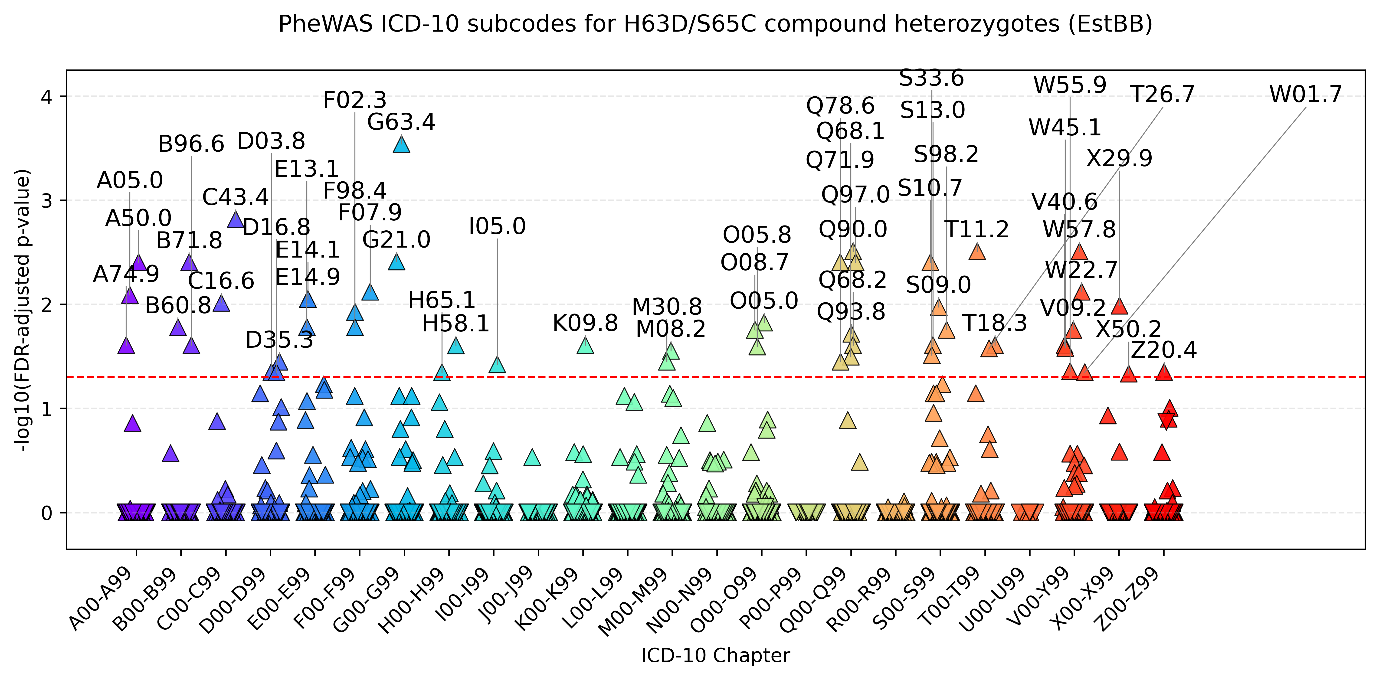


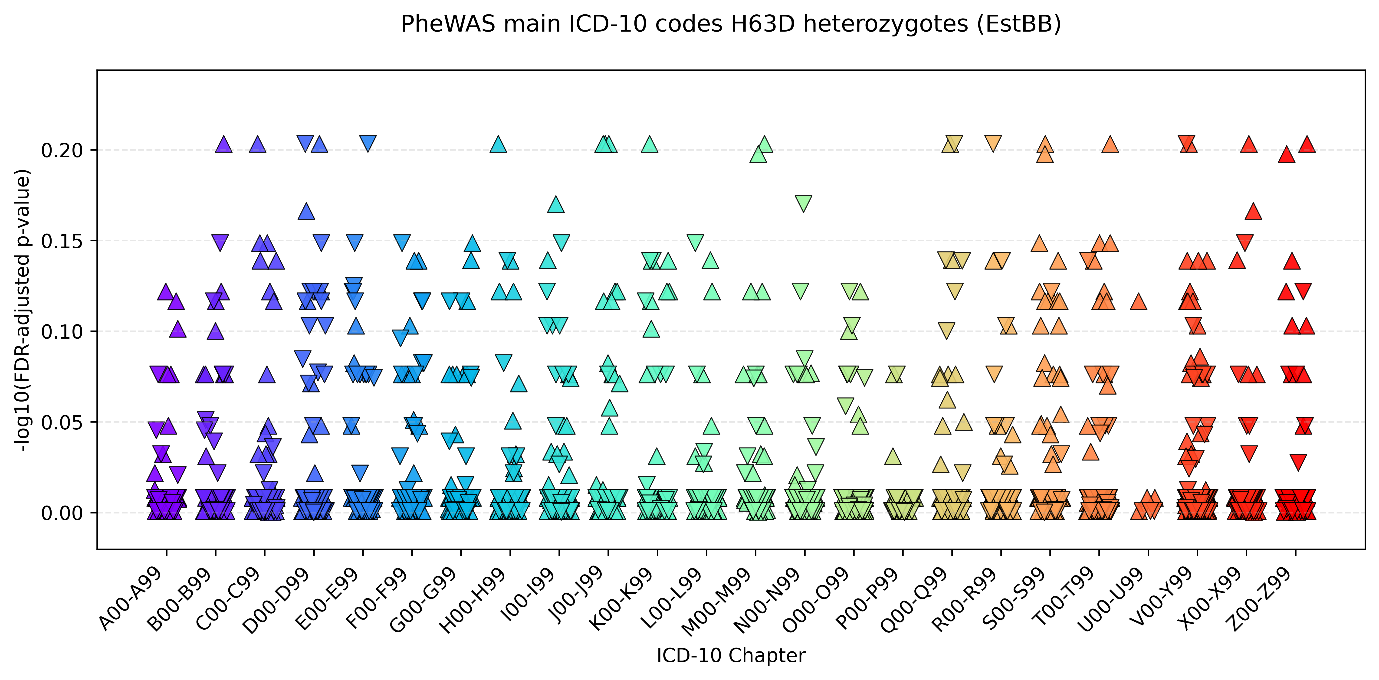


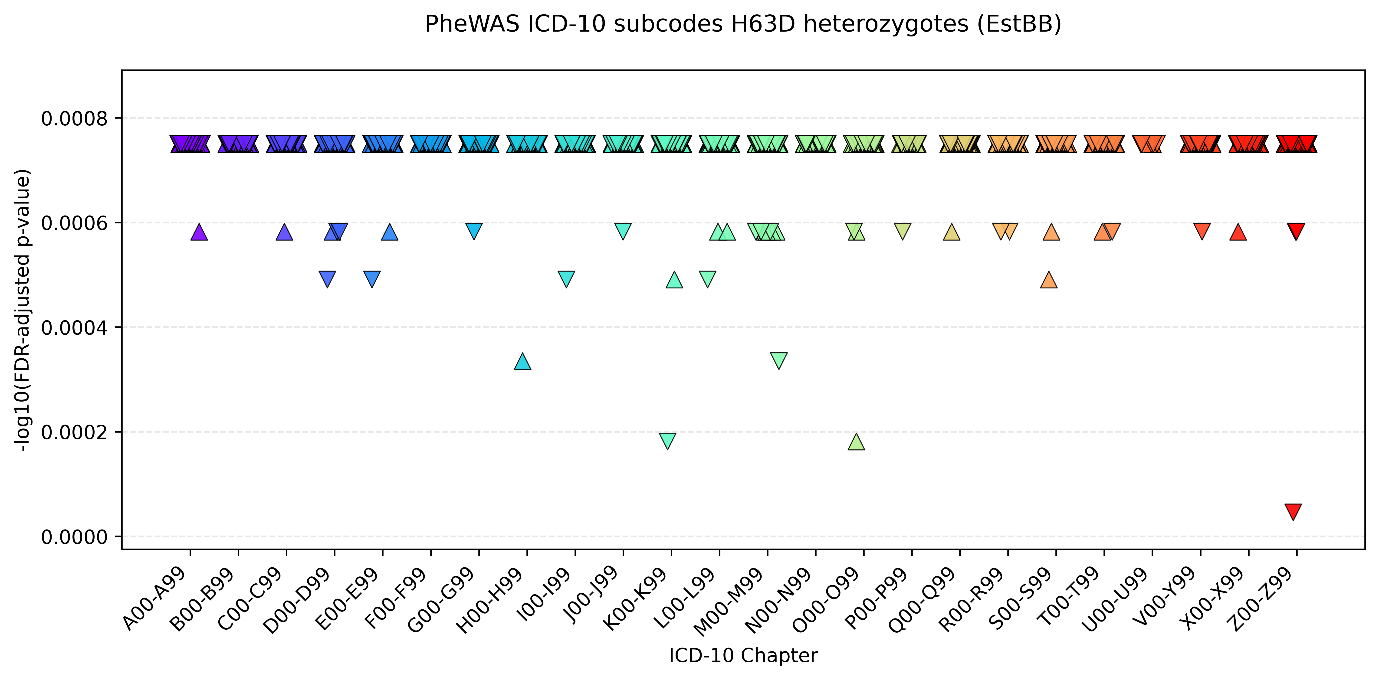


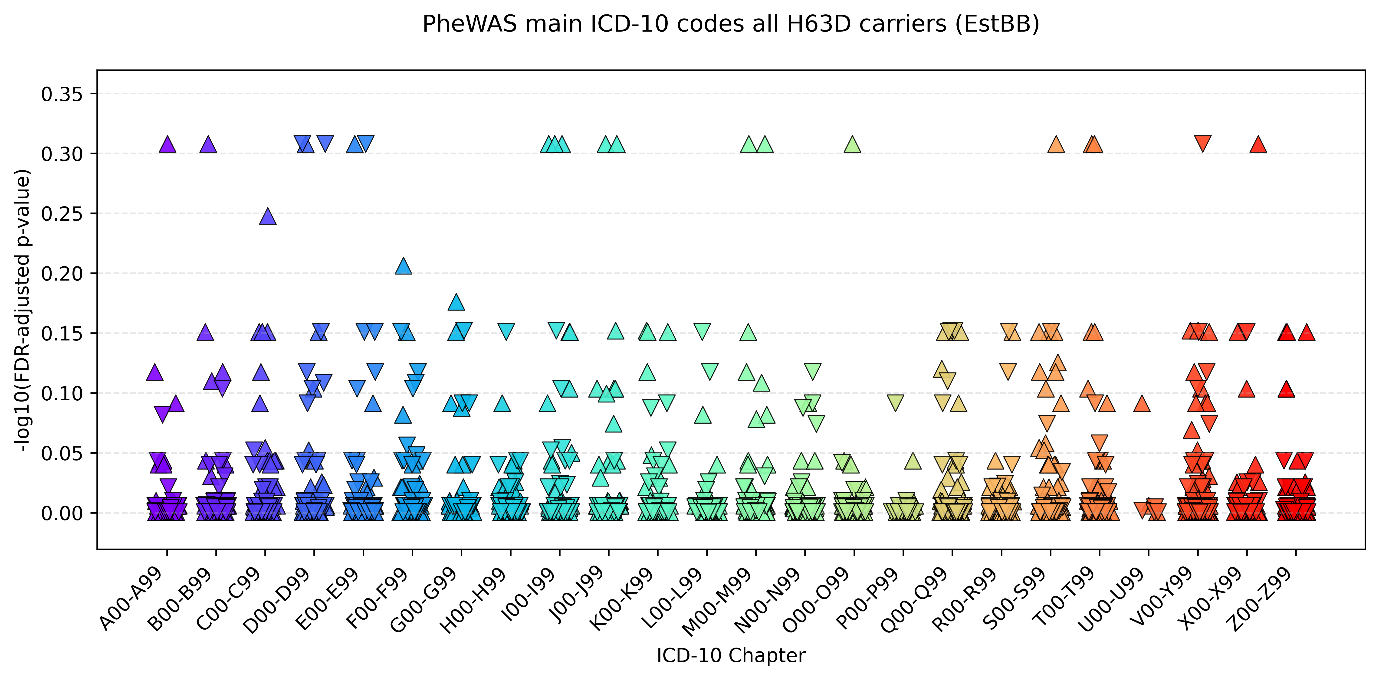


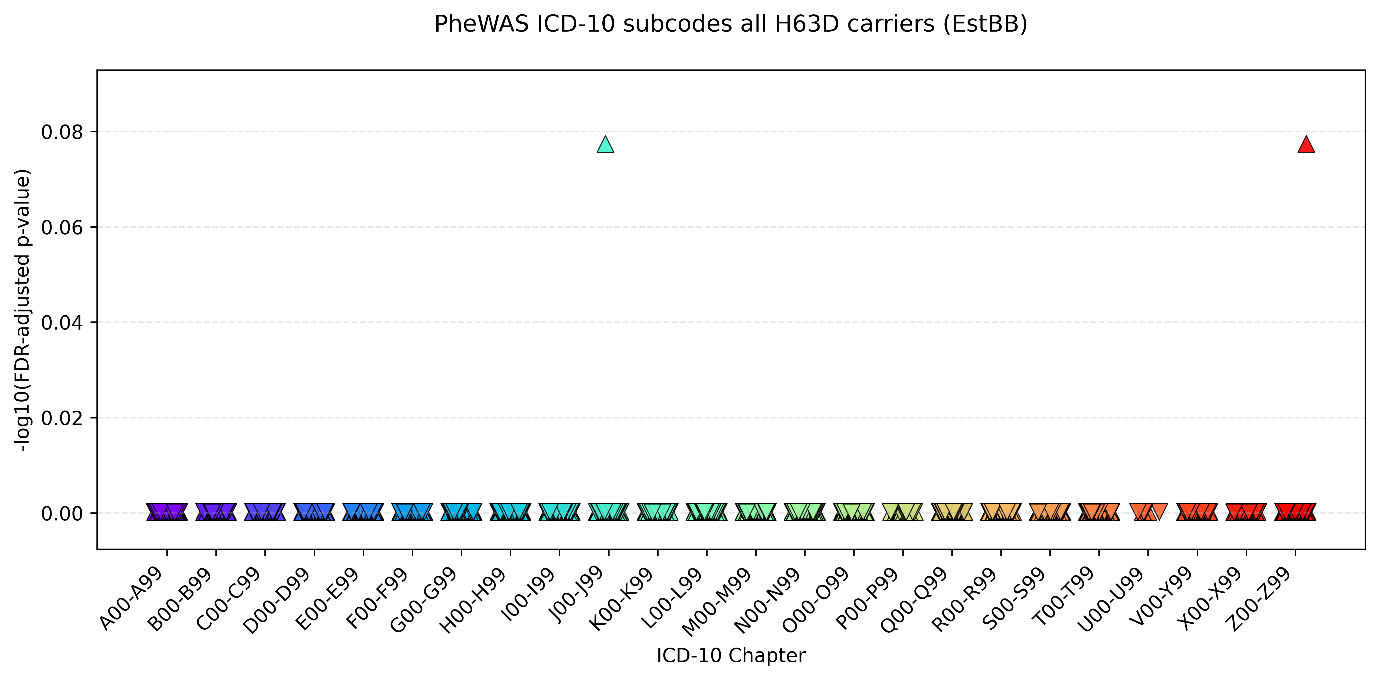


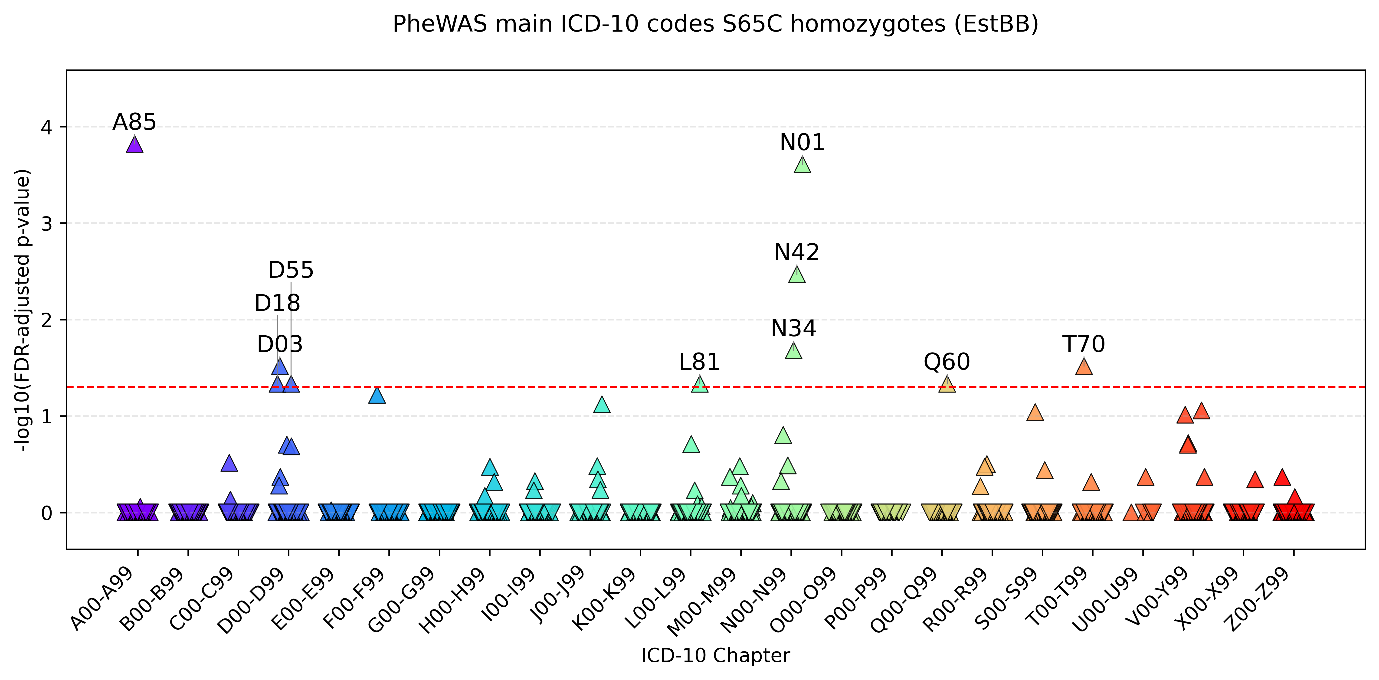


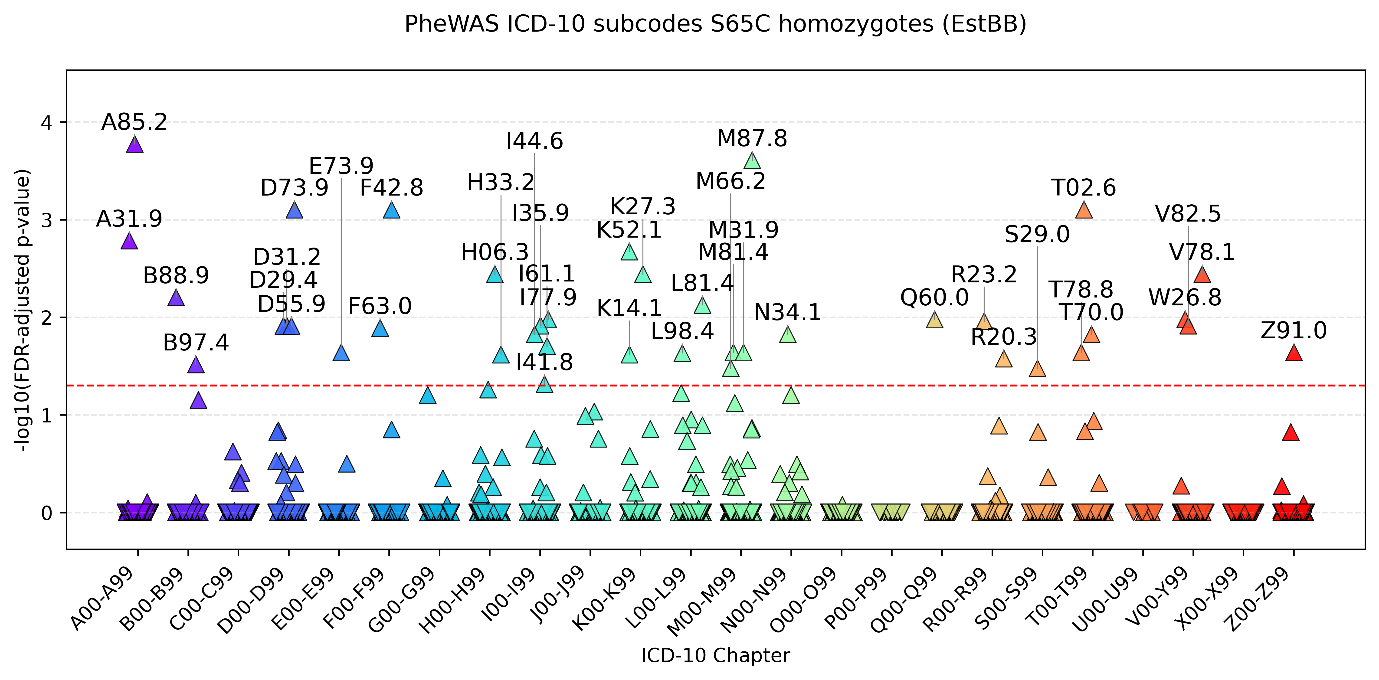


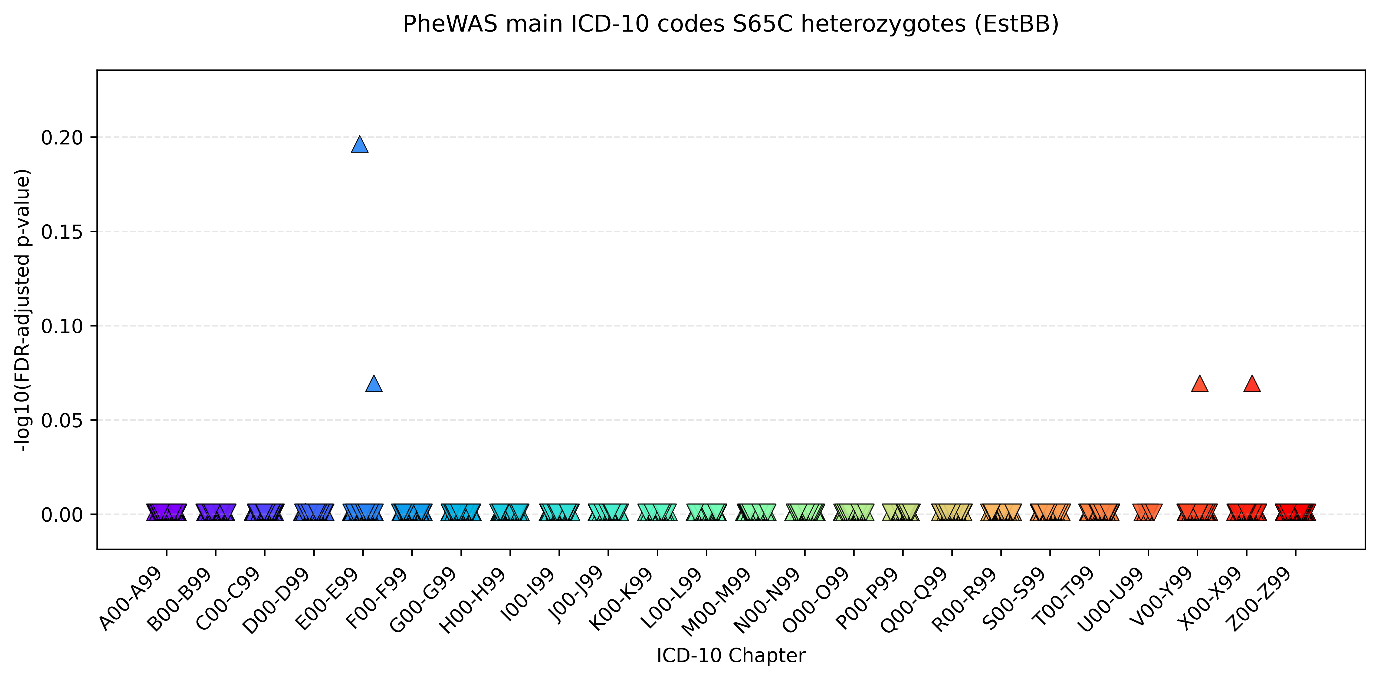


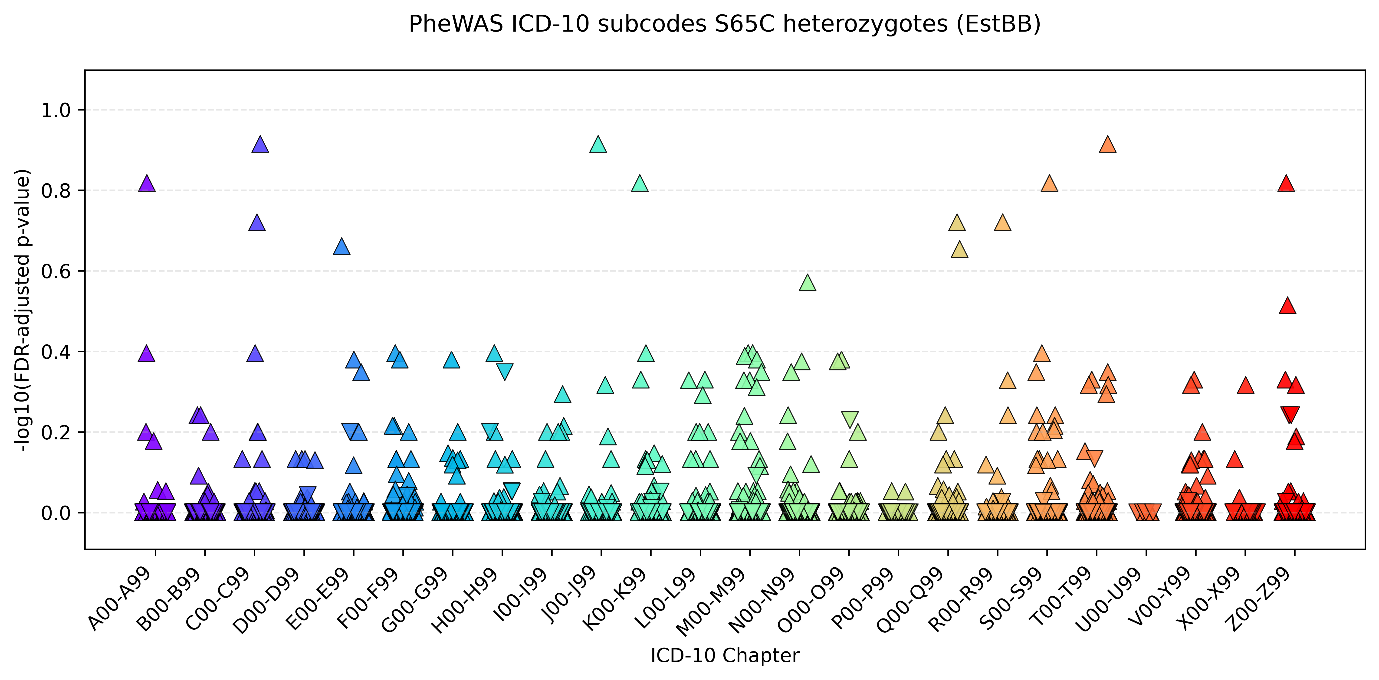


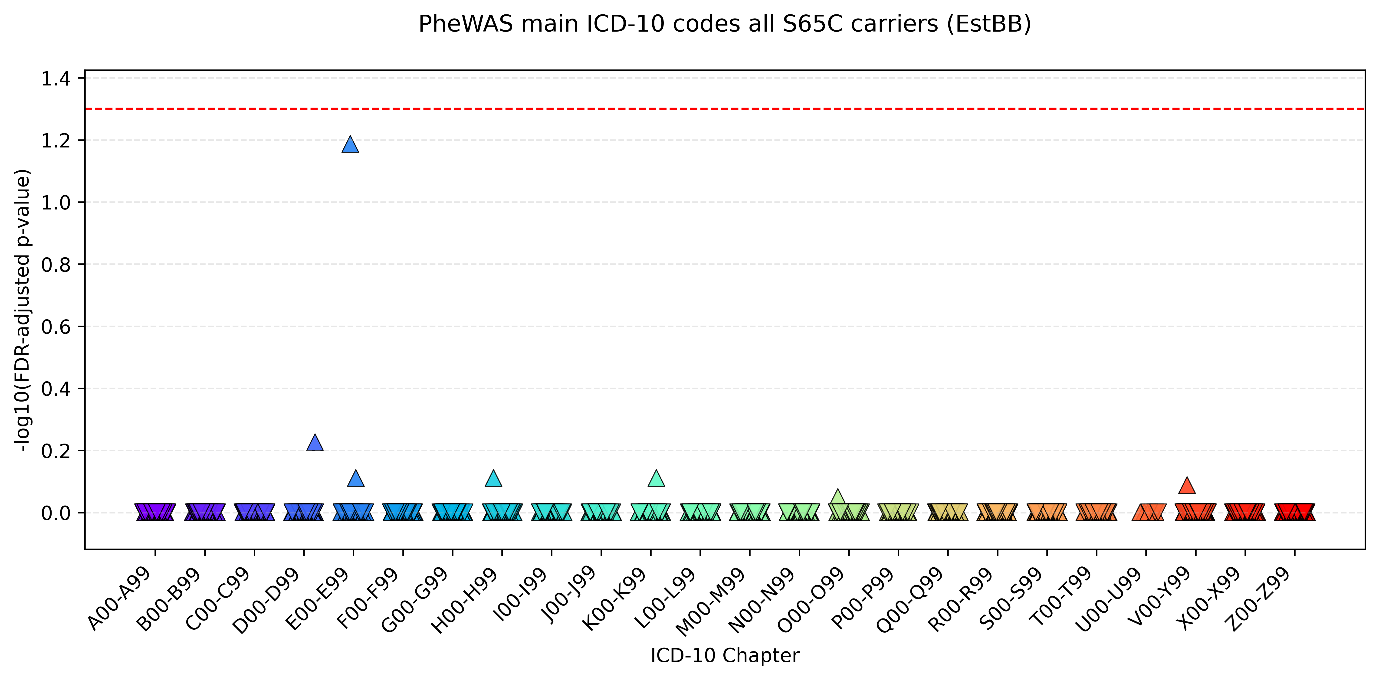


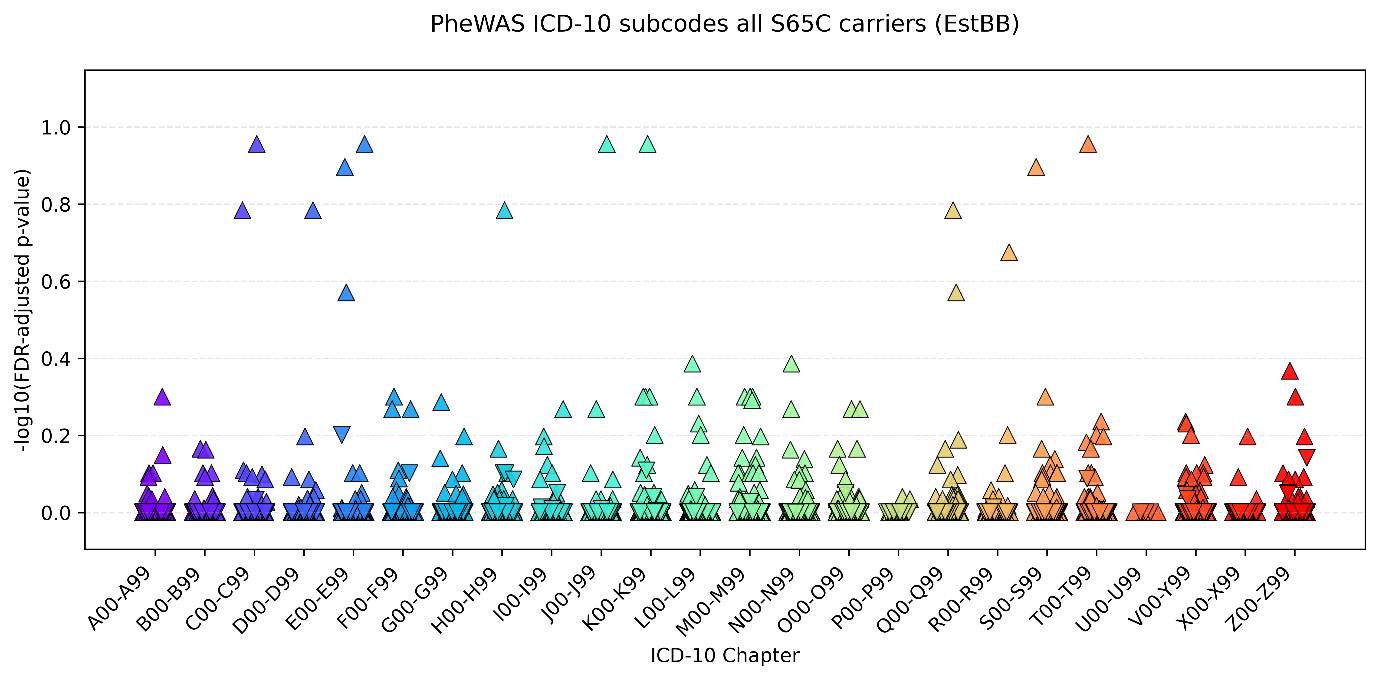


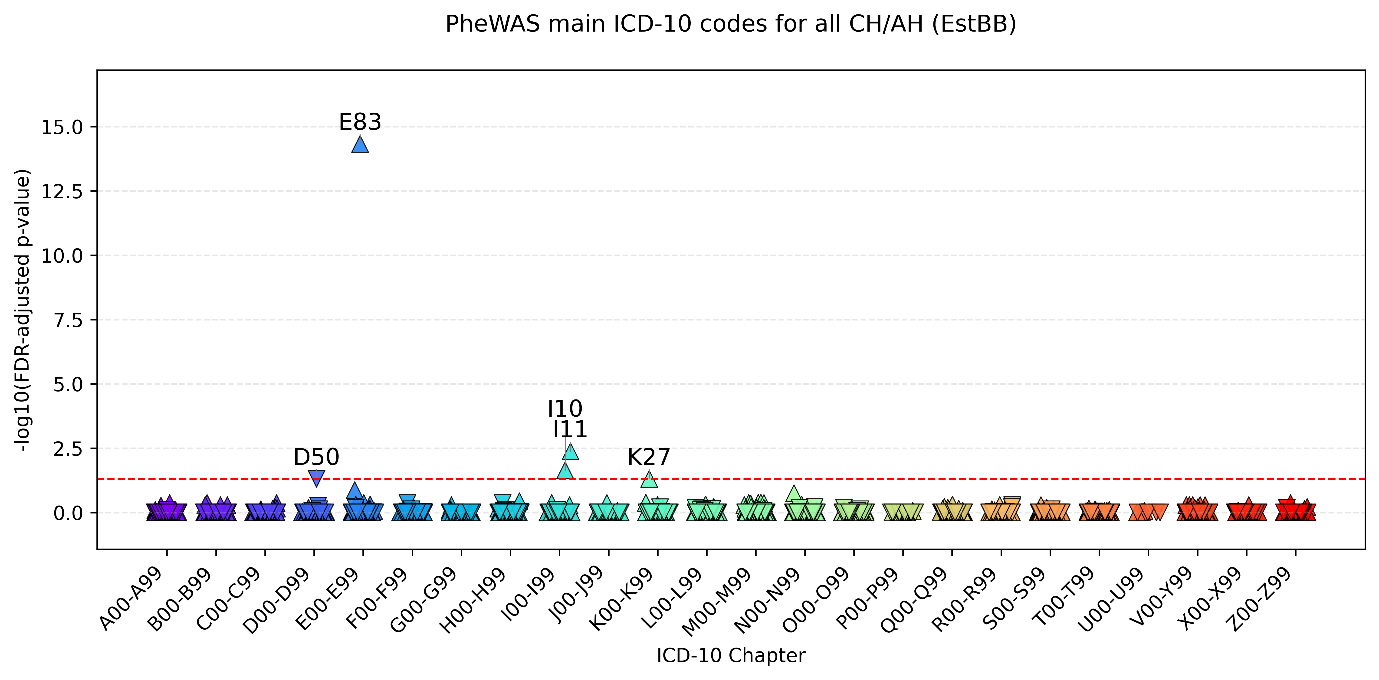


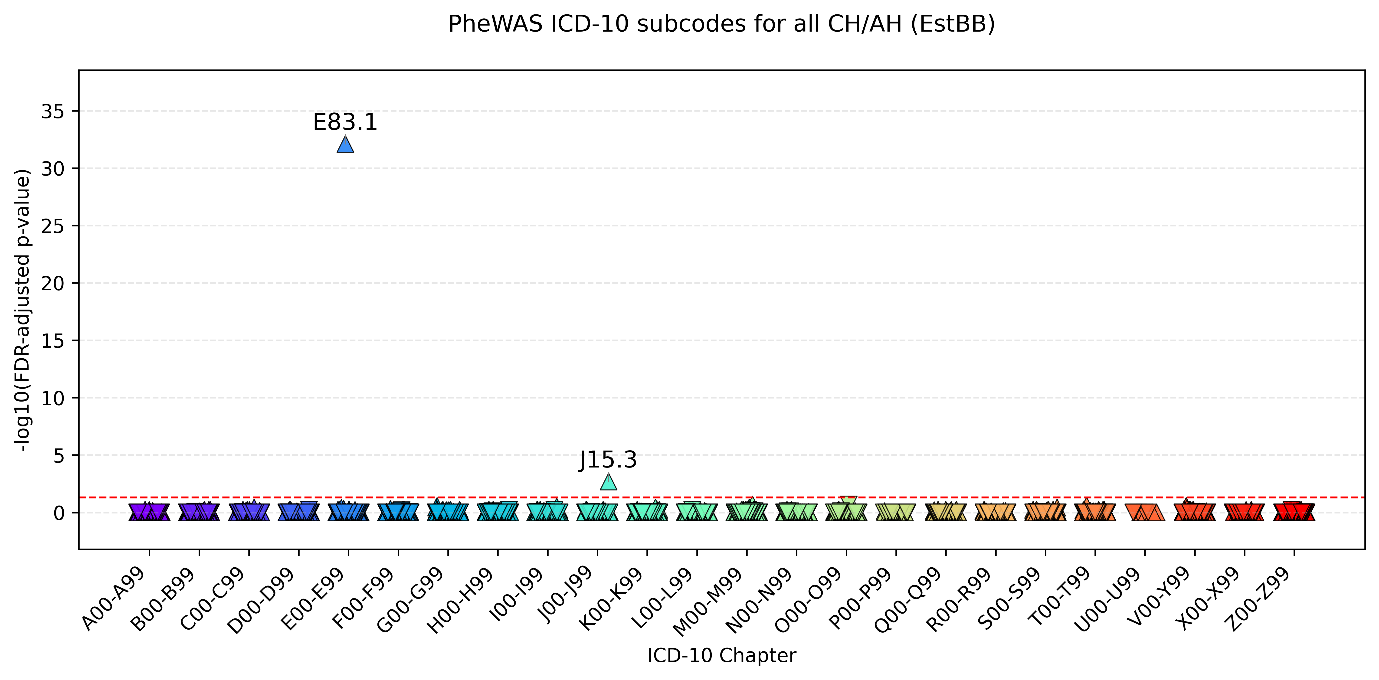


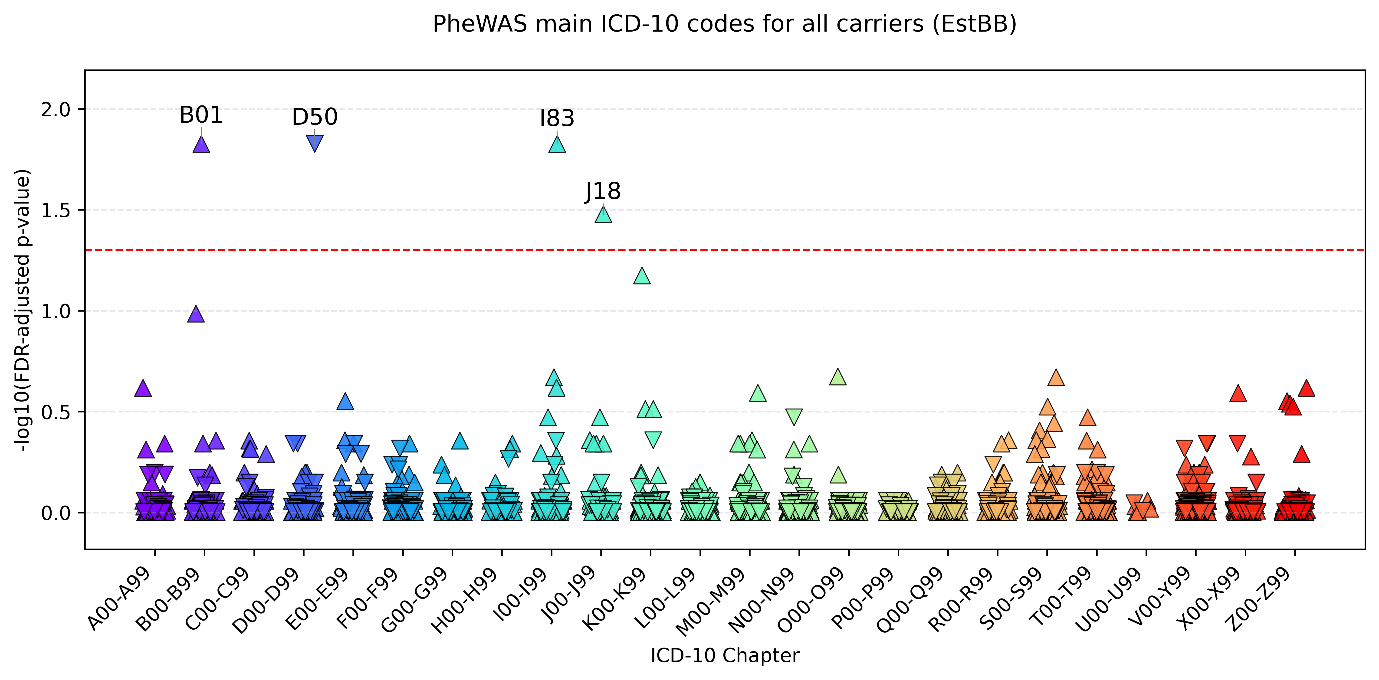

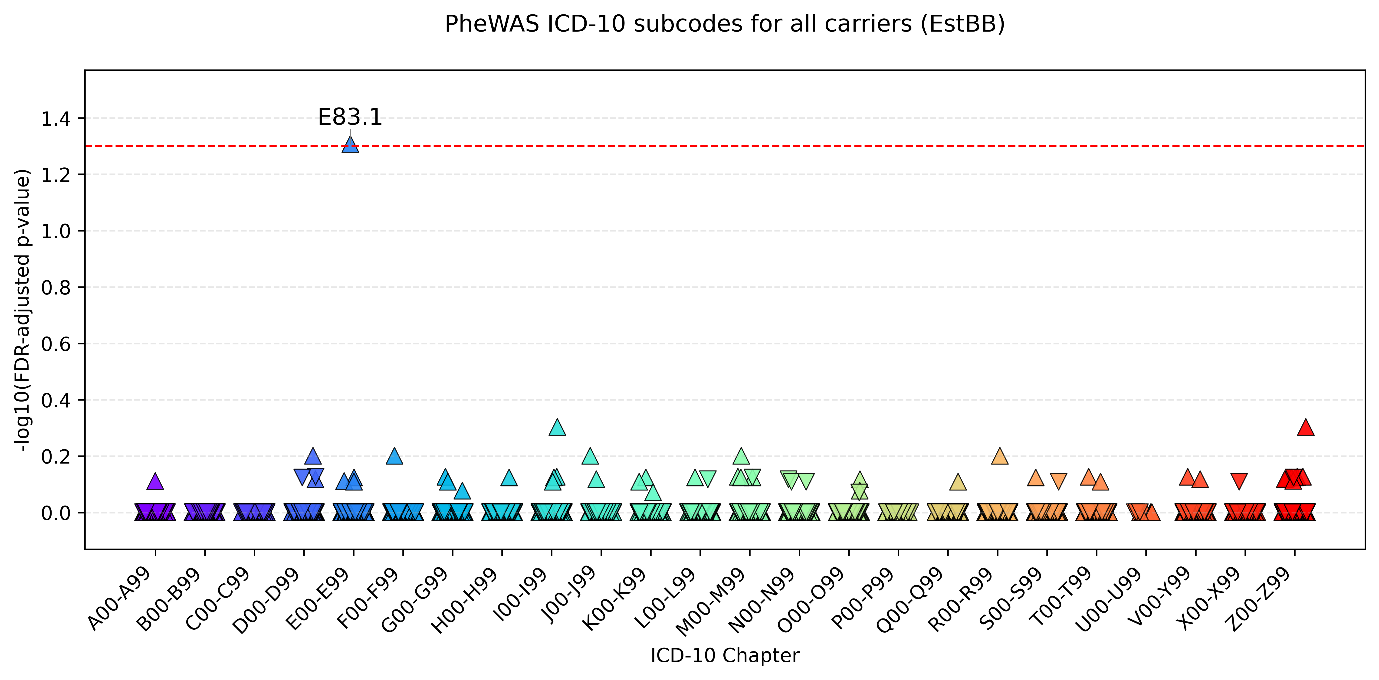


# Supplementary Figs. S29–S56. Manhattan plots of UKB PheWAS results.

**Supplementary Figs. S29–S56.** Manhattan plots of UKB PheWAS results. Five-character codes beginning with “M” have been collapsed into their four-character parent codes where applicable. UKB, UK Biobank; PheWAS, phenome-wide association study; ICD-10, International Classification of Diseases, 10^th^ Revision; FDR, false discovery rate; CH, compound heterozygote; AH, alternative homozygote.


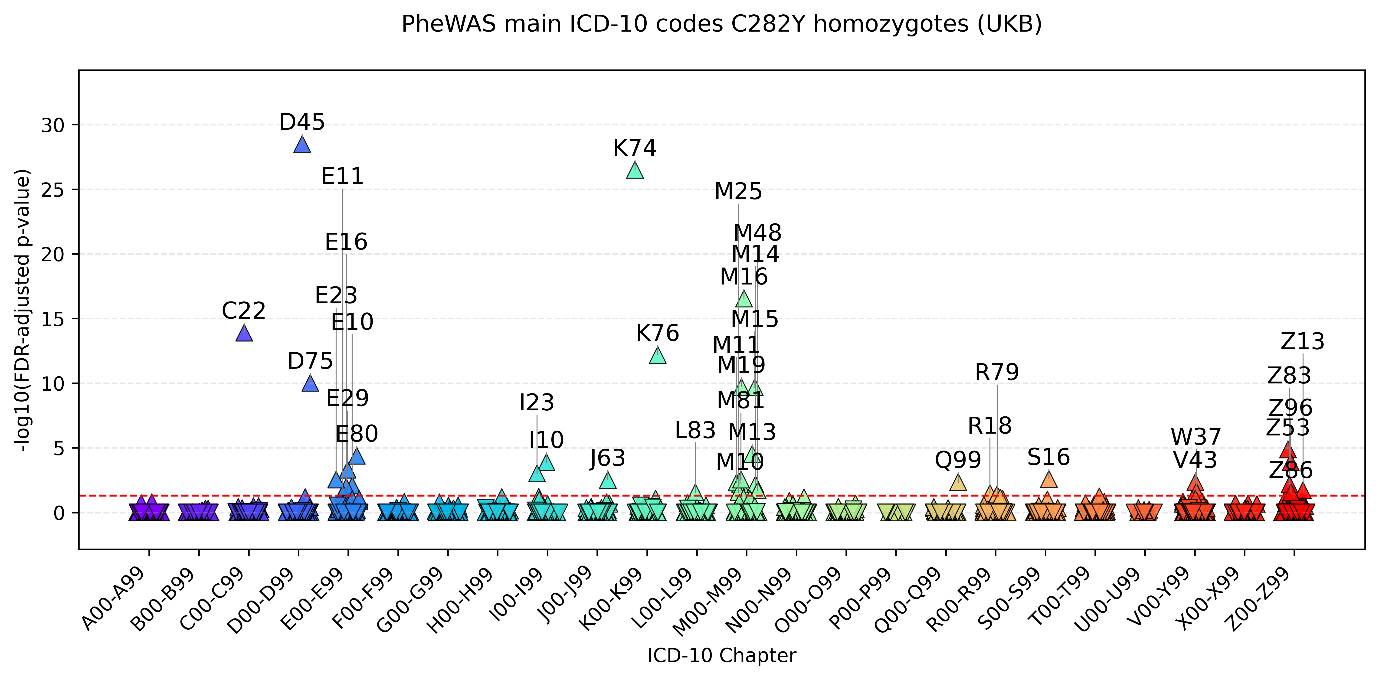


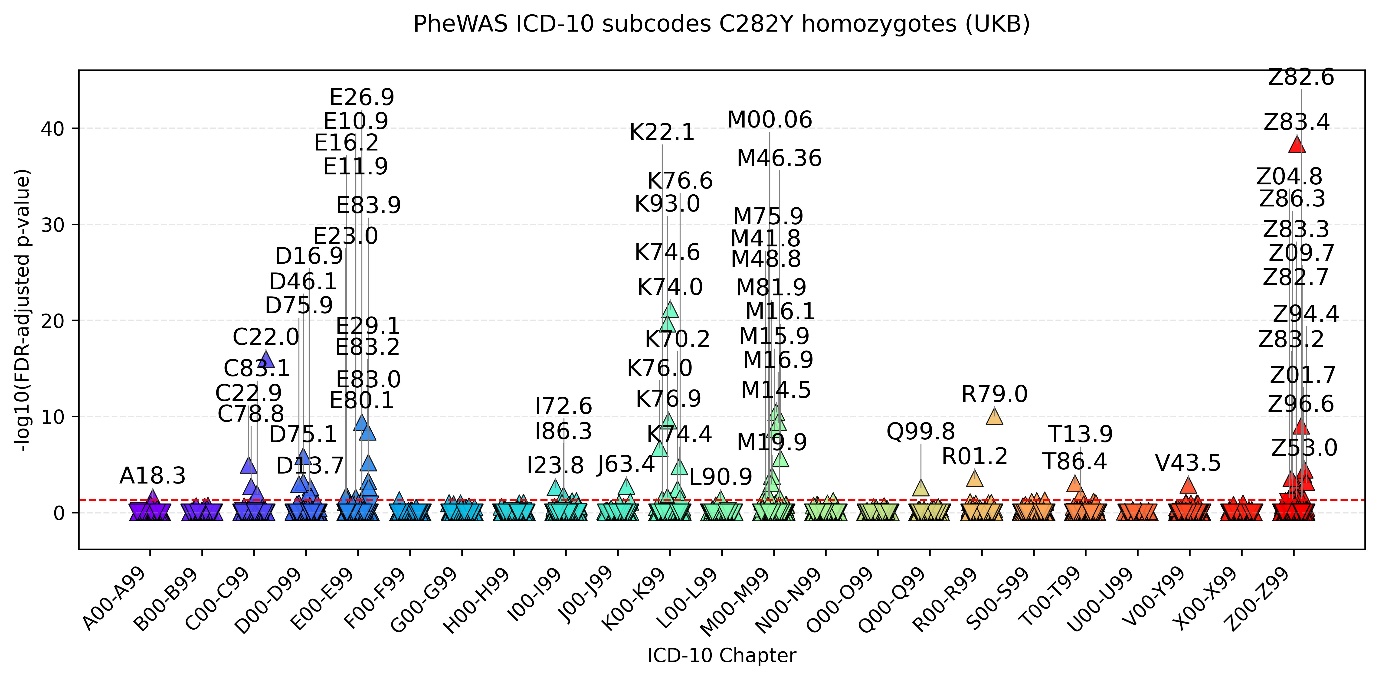


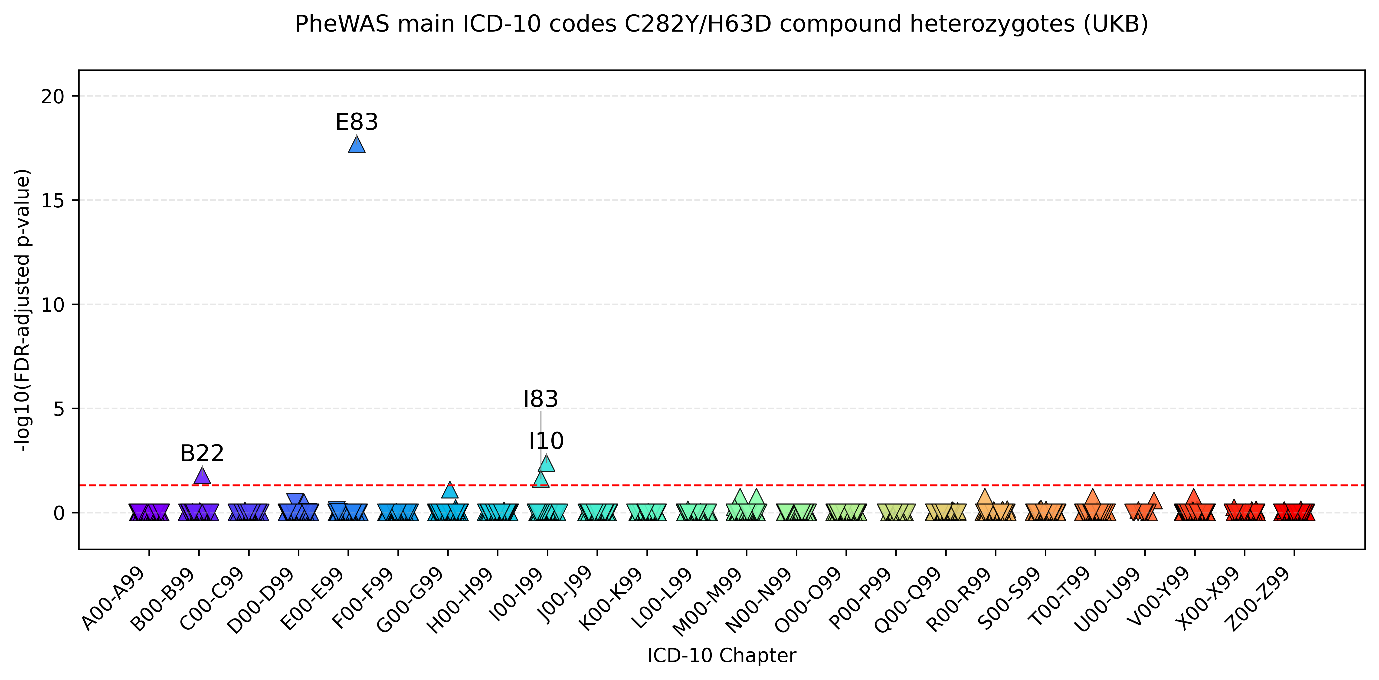


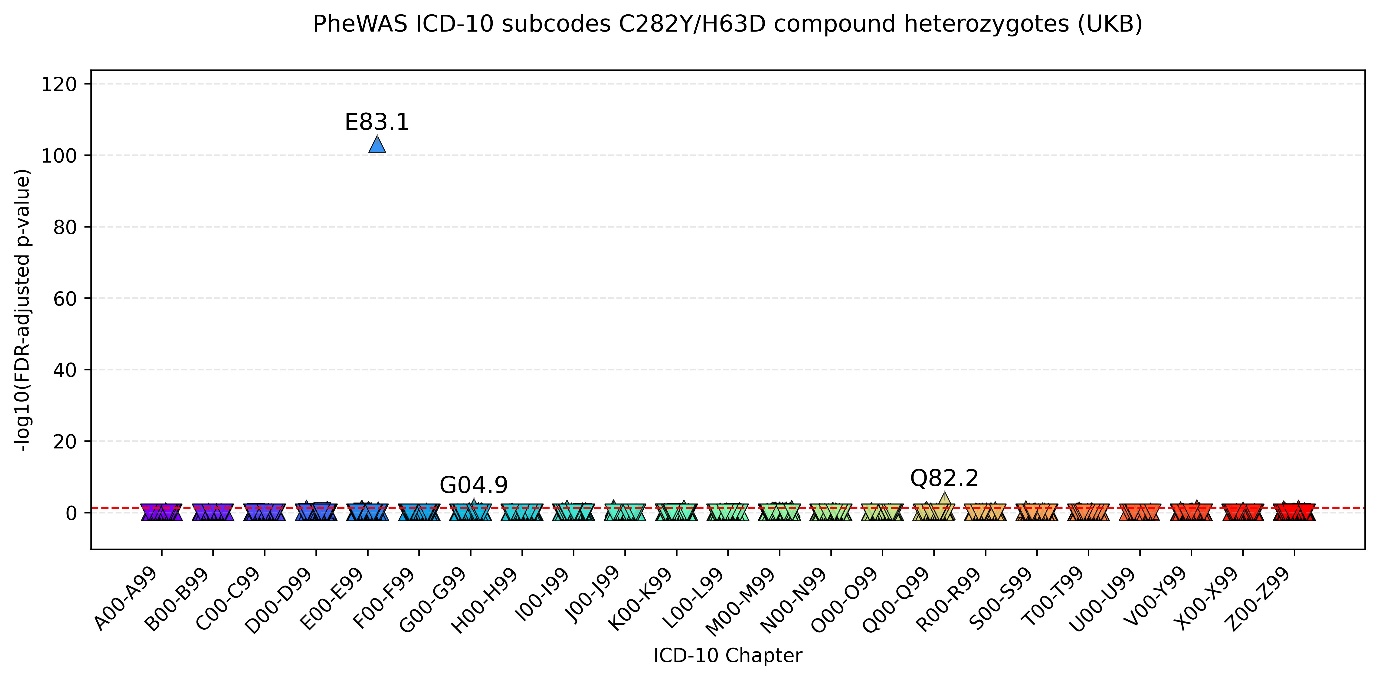


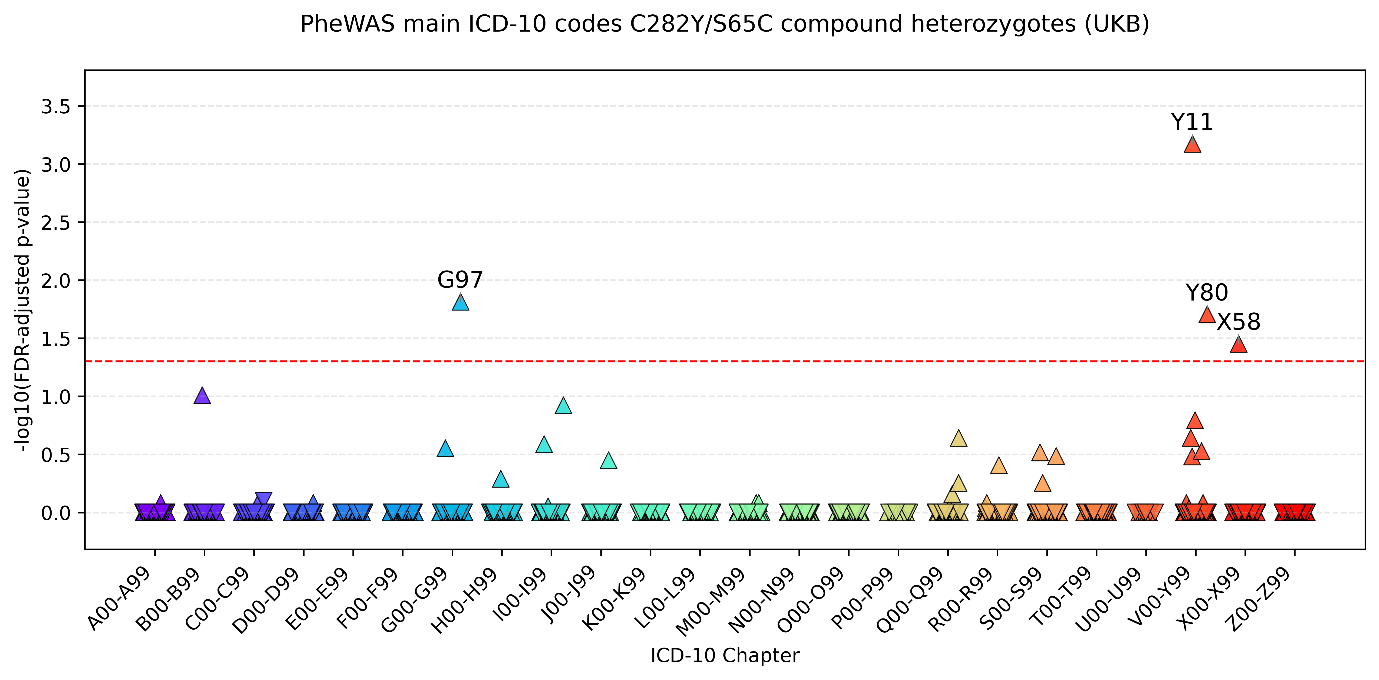

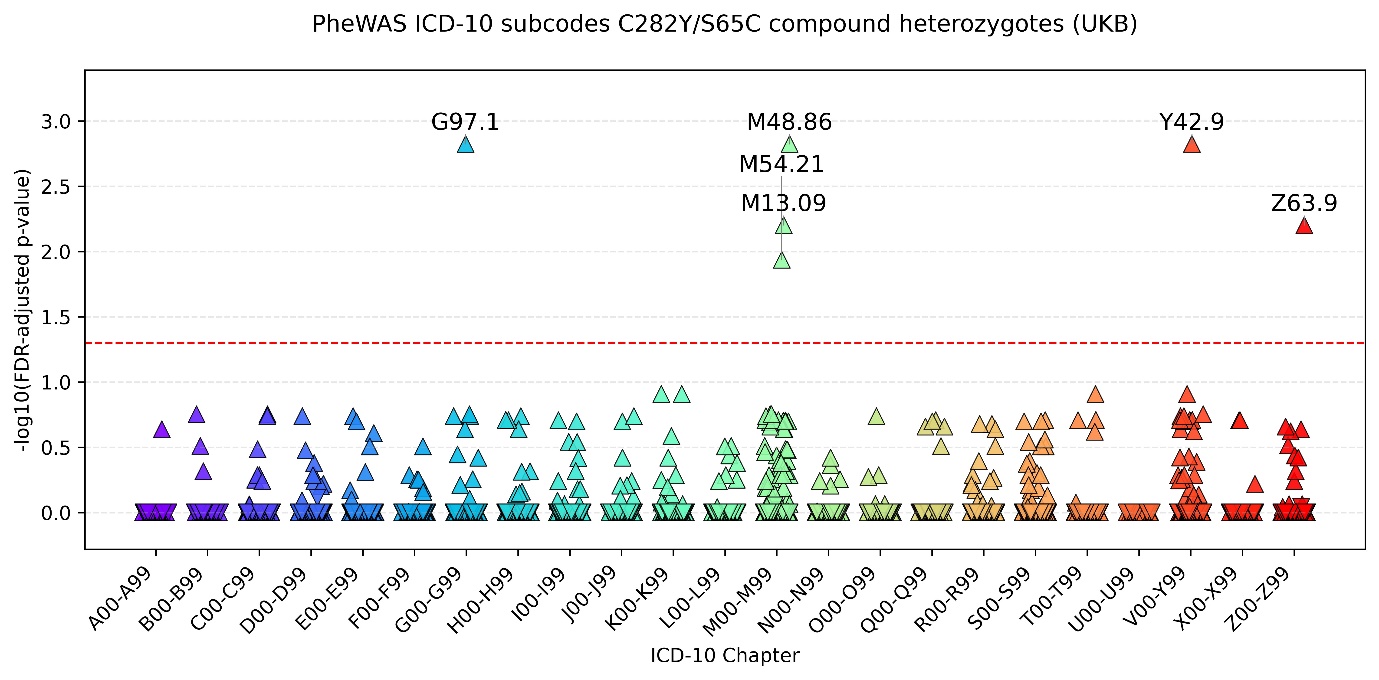


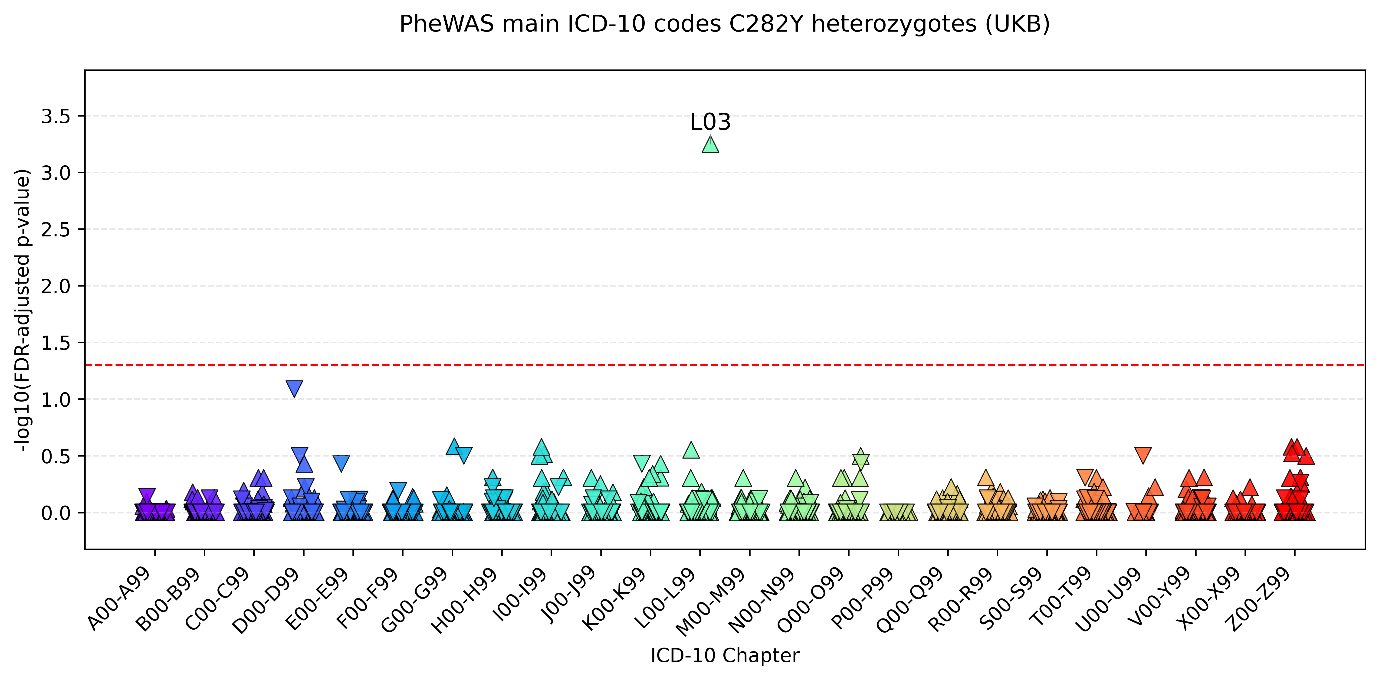

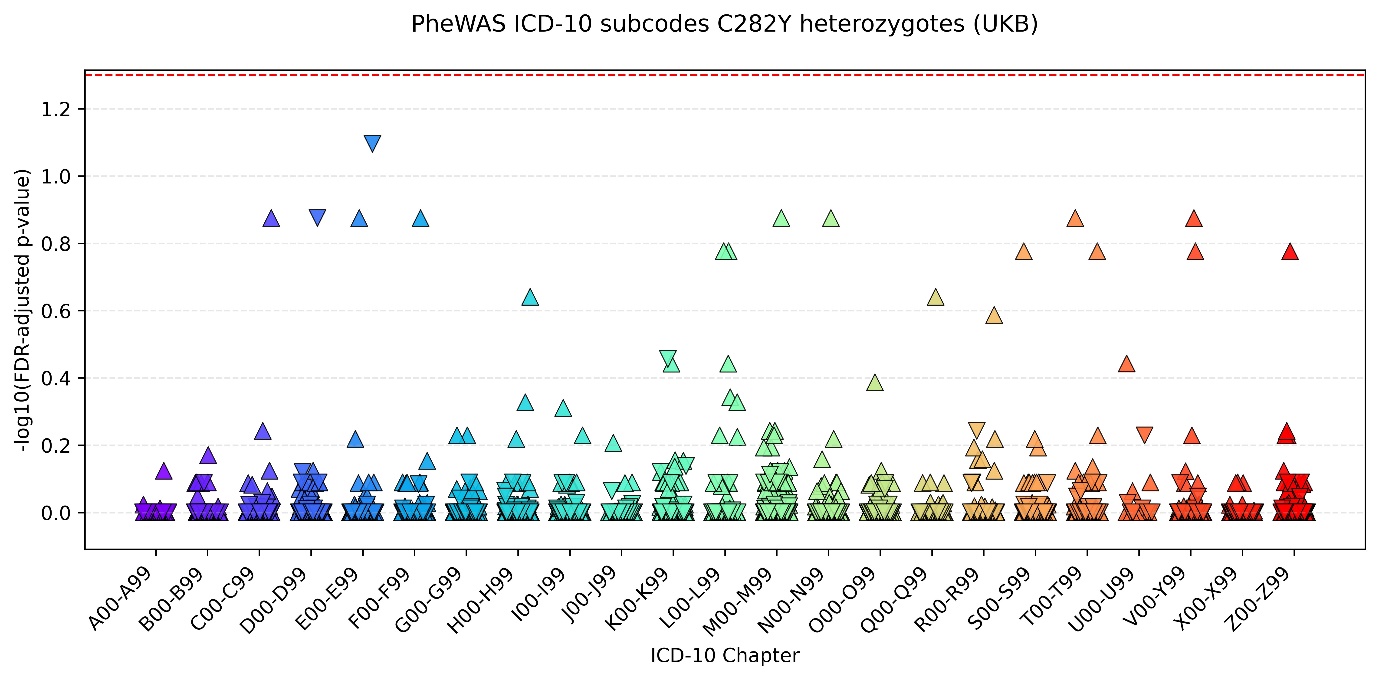


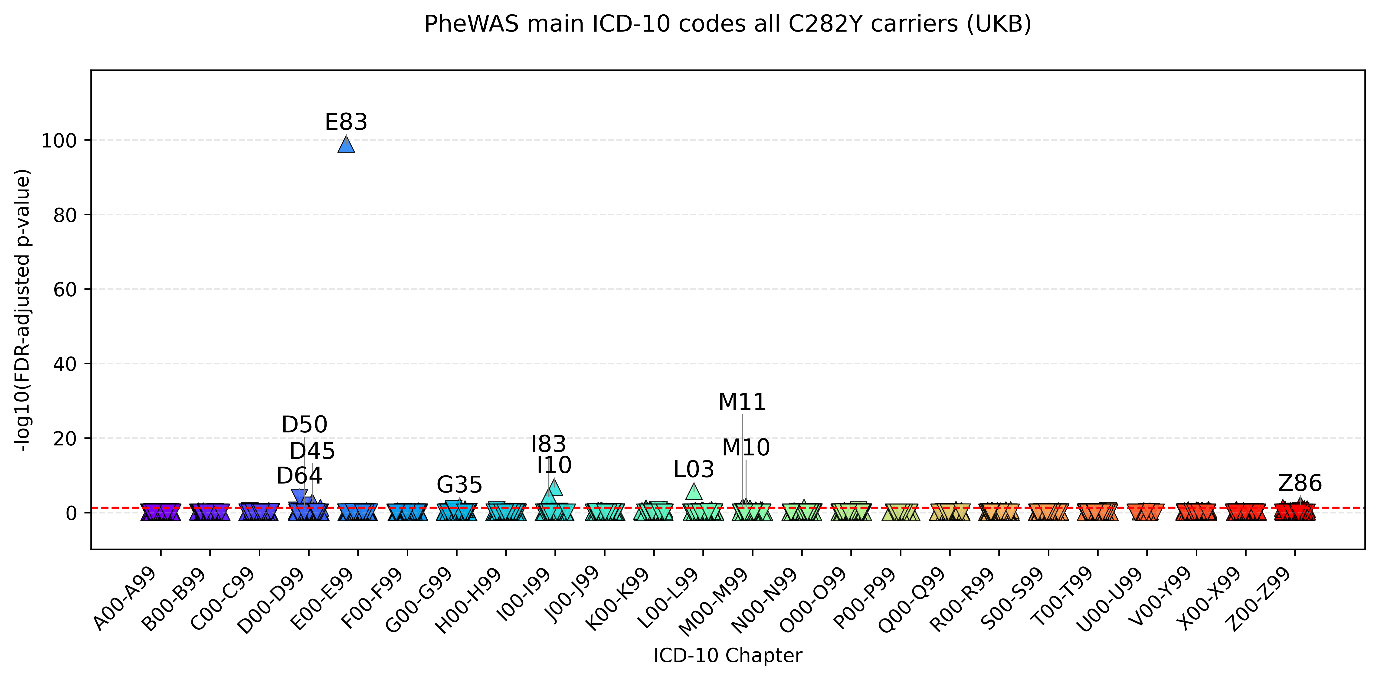

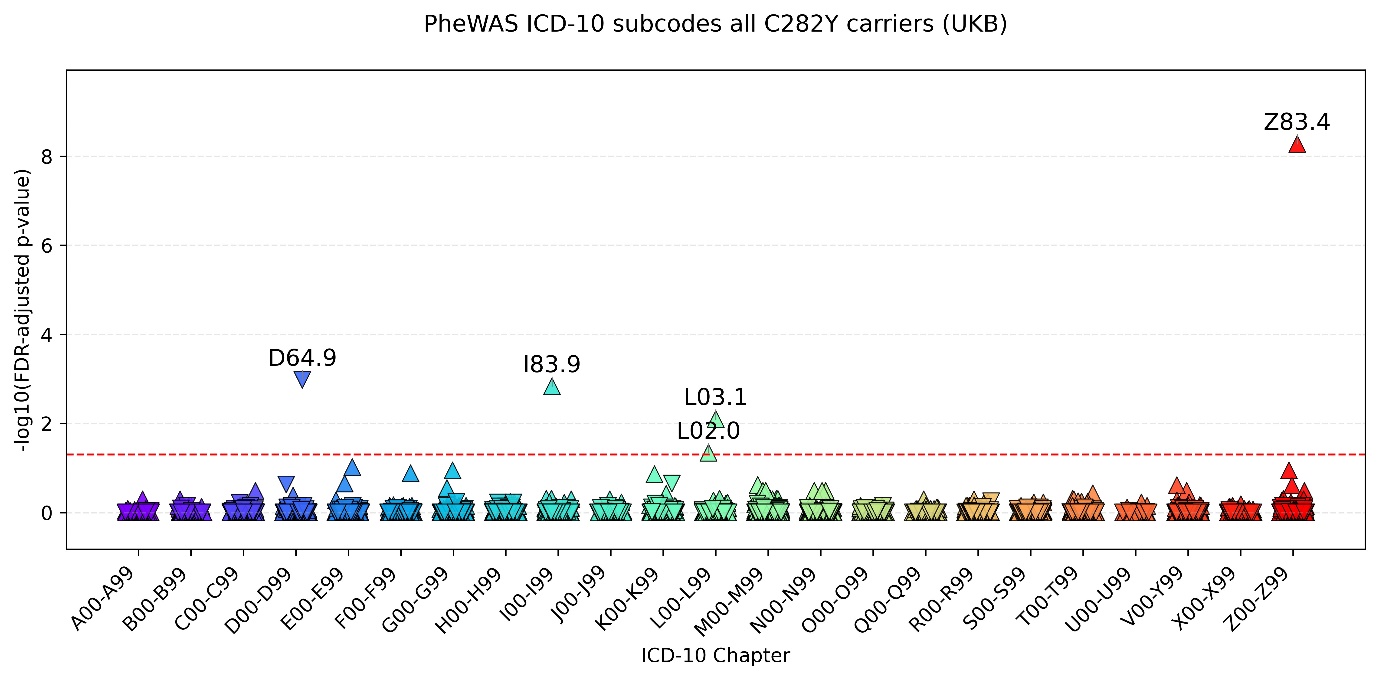


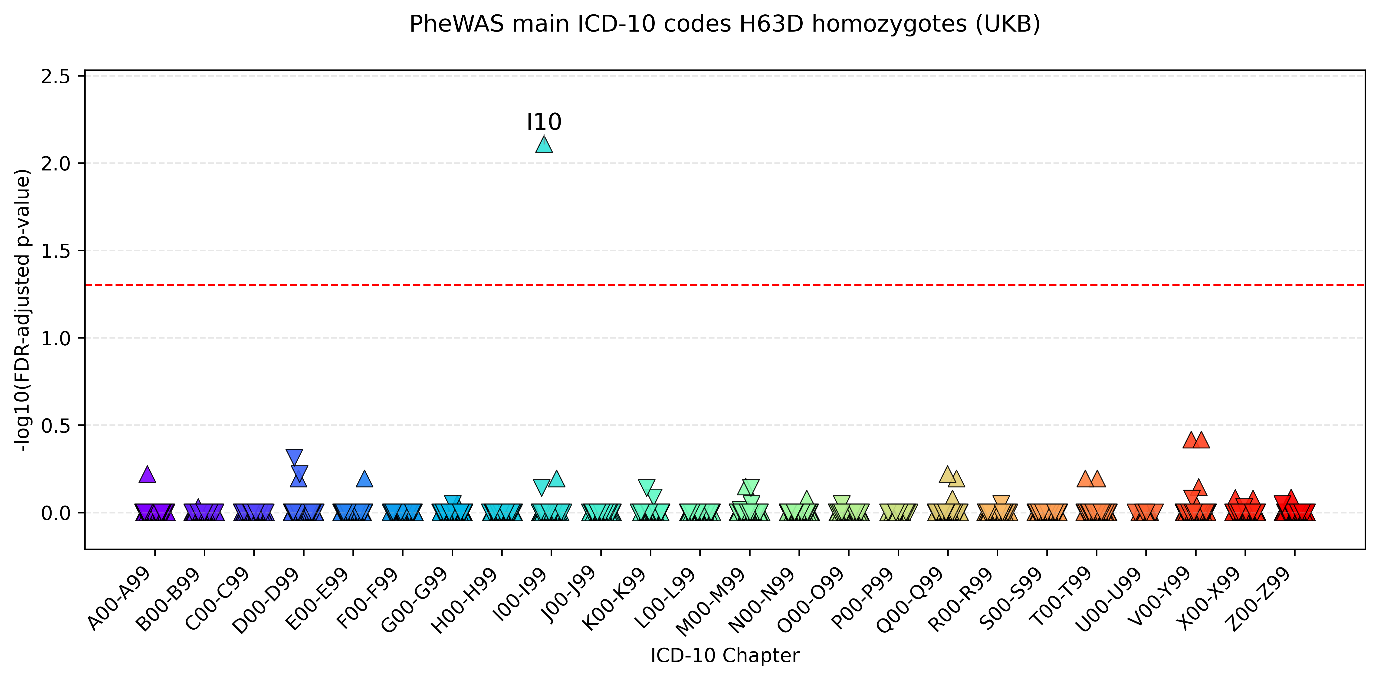

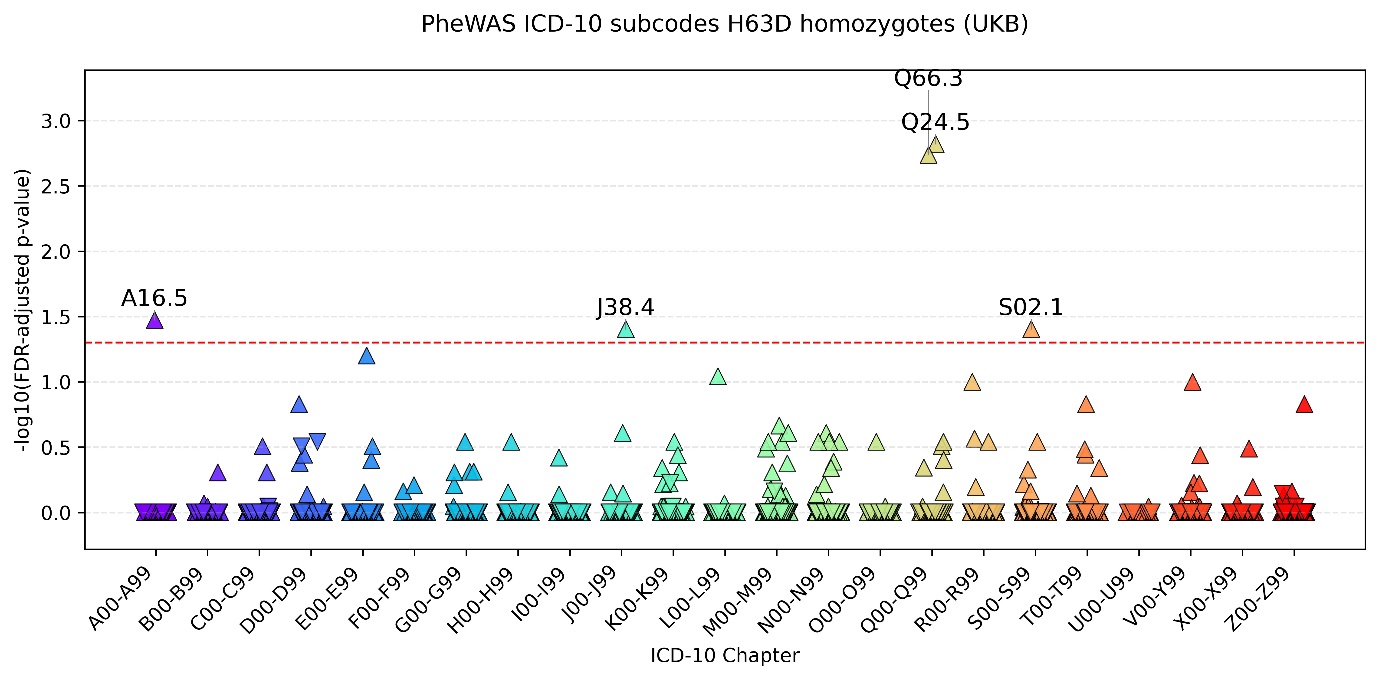


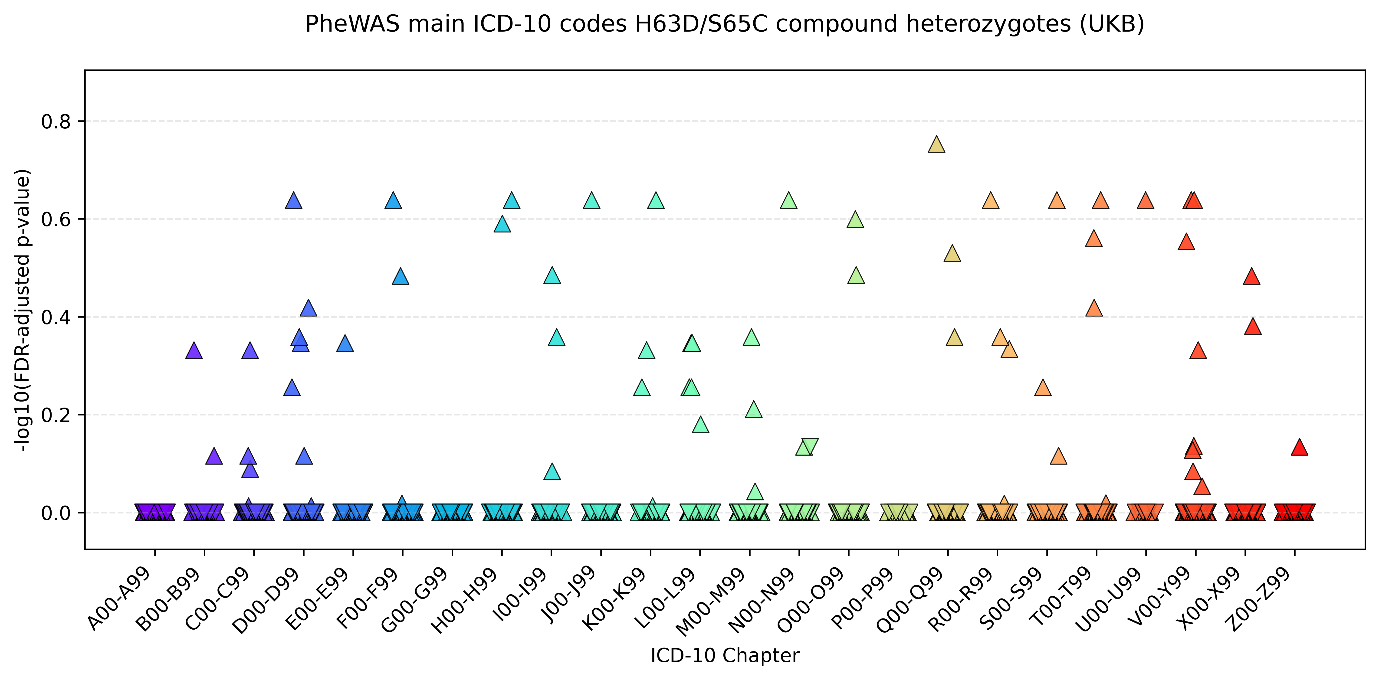

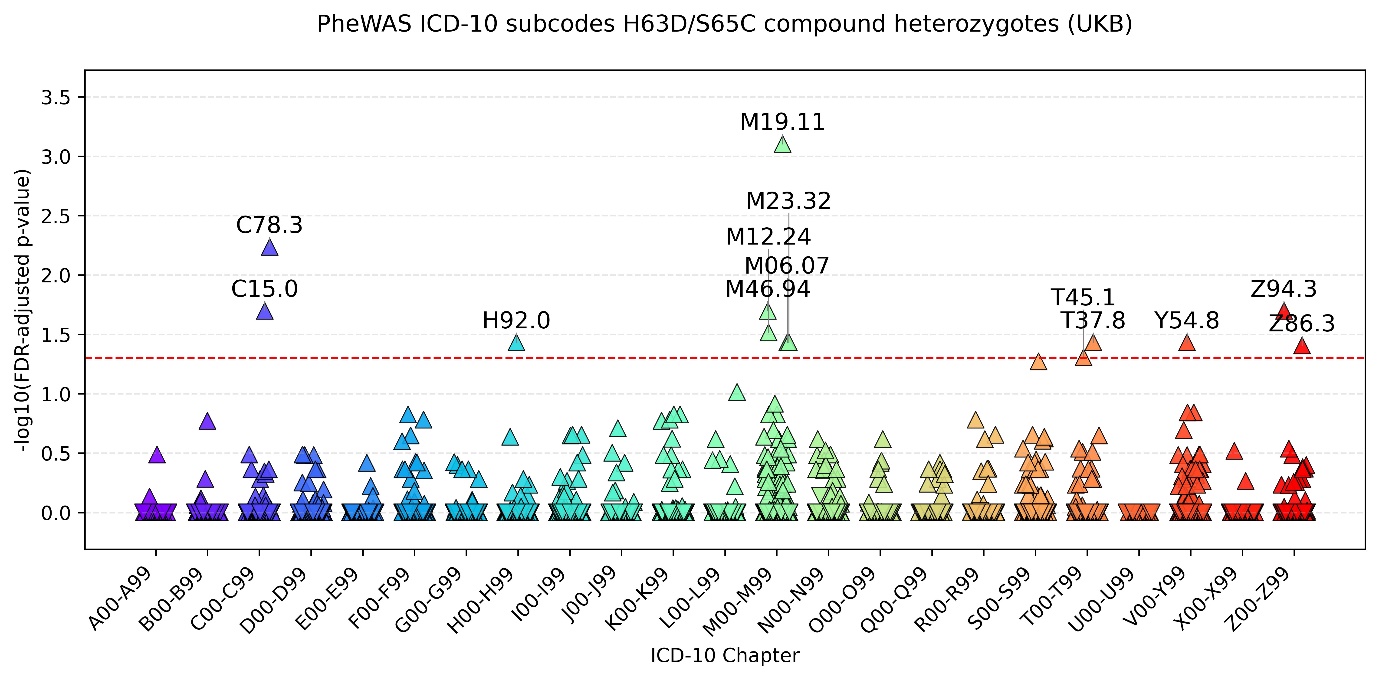


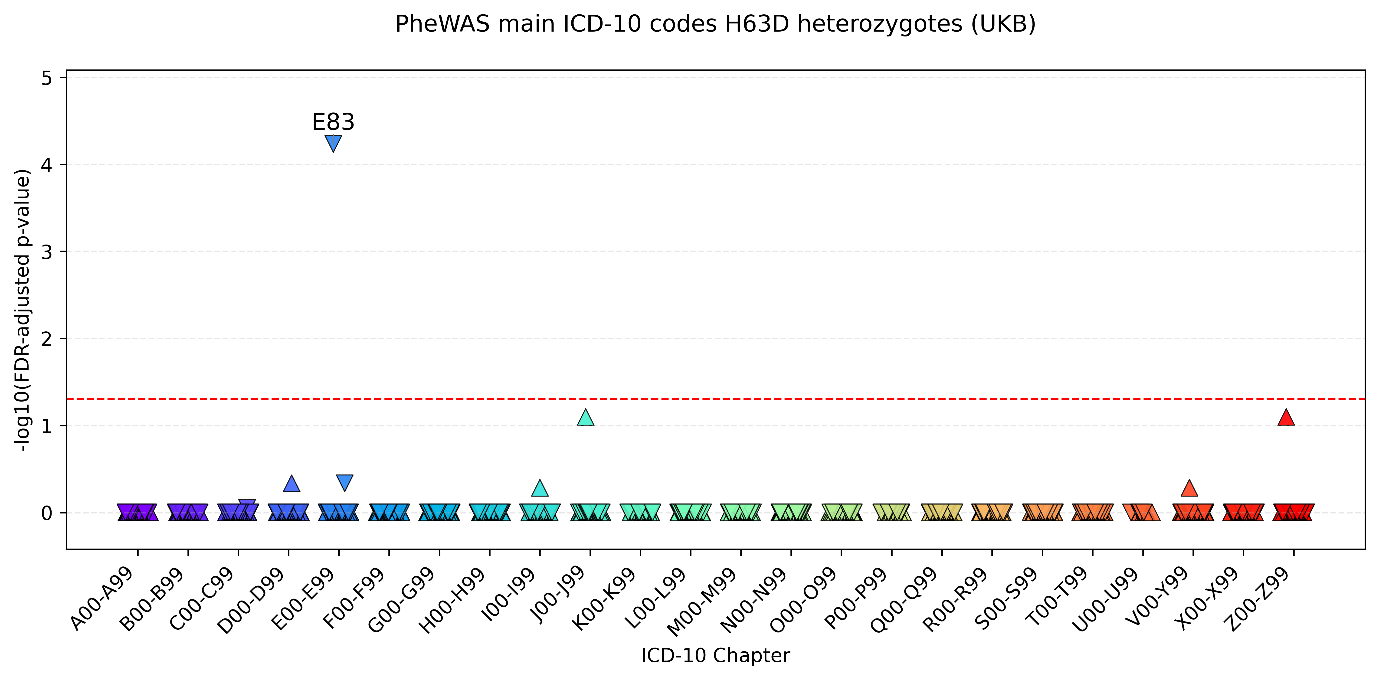

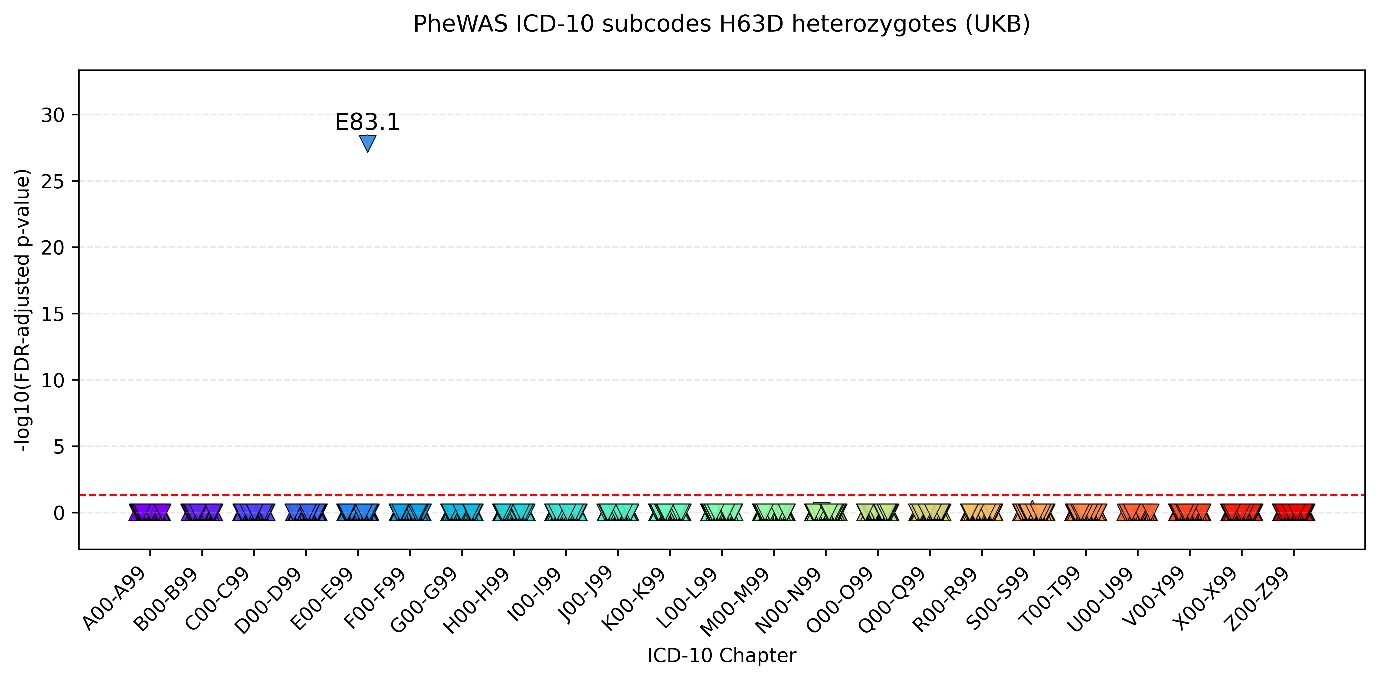


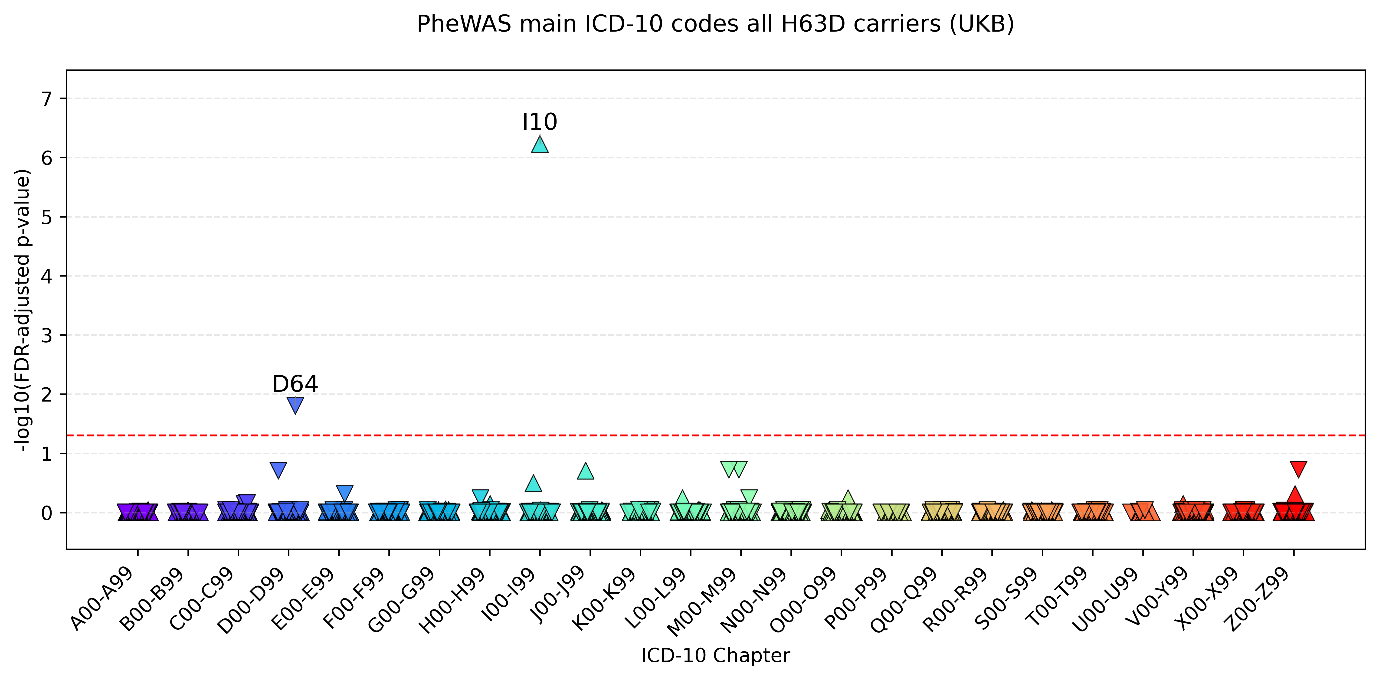

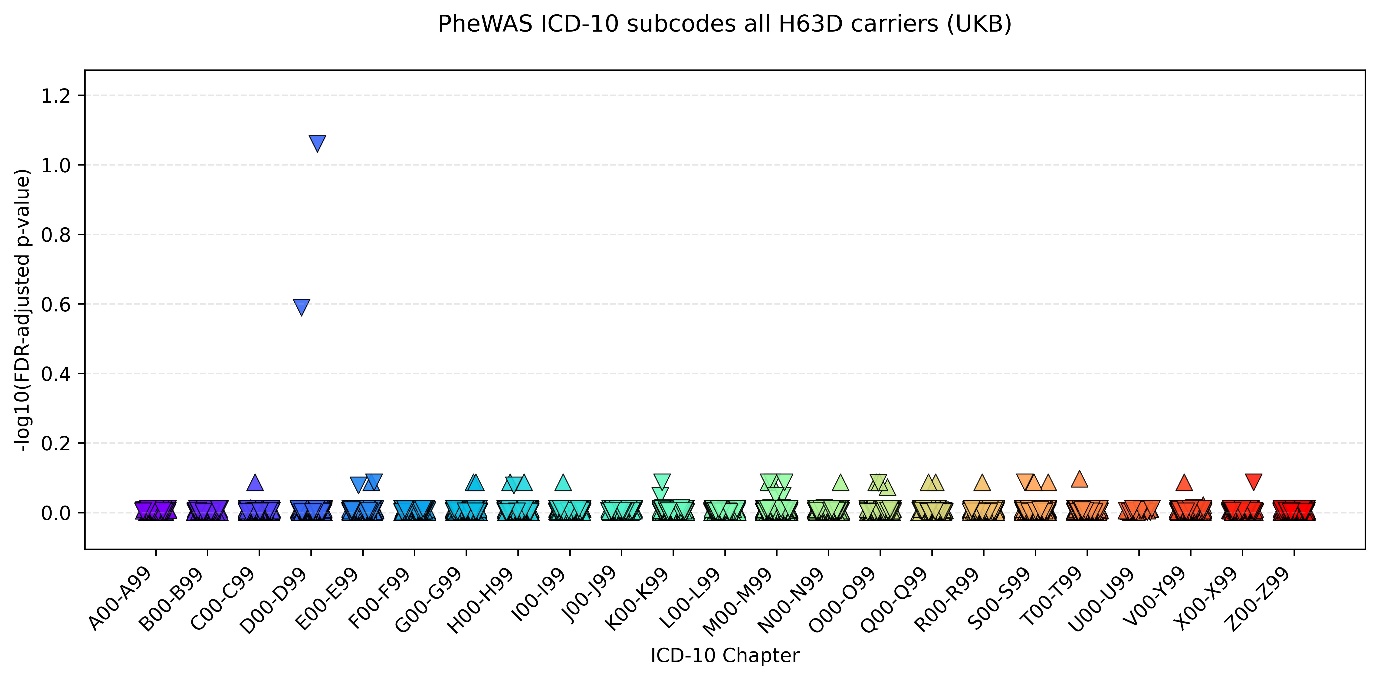


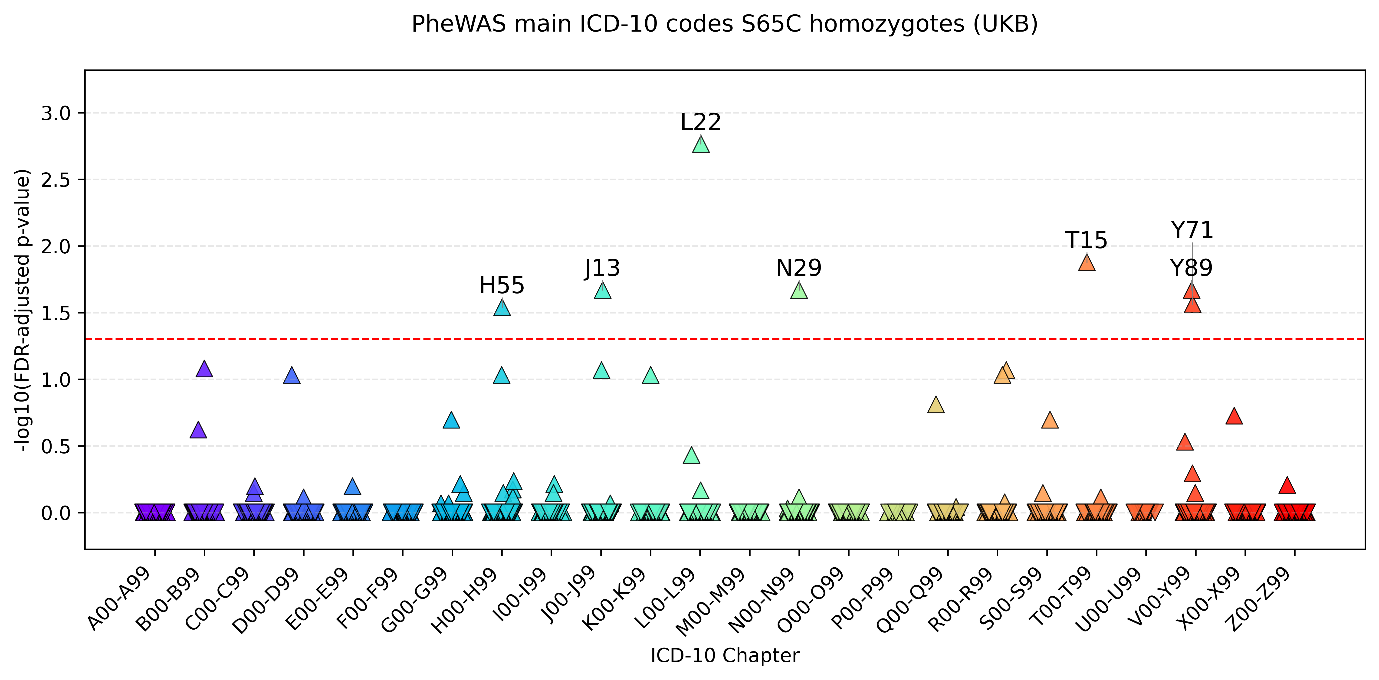

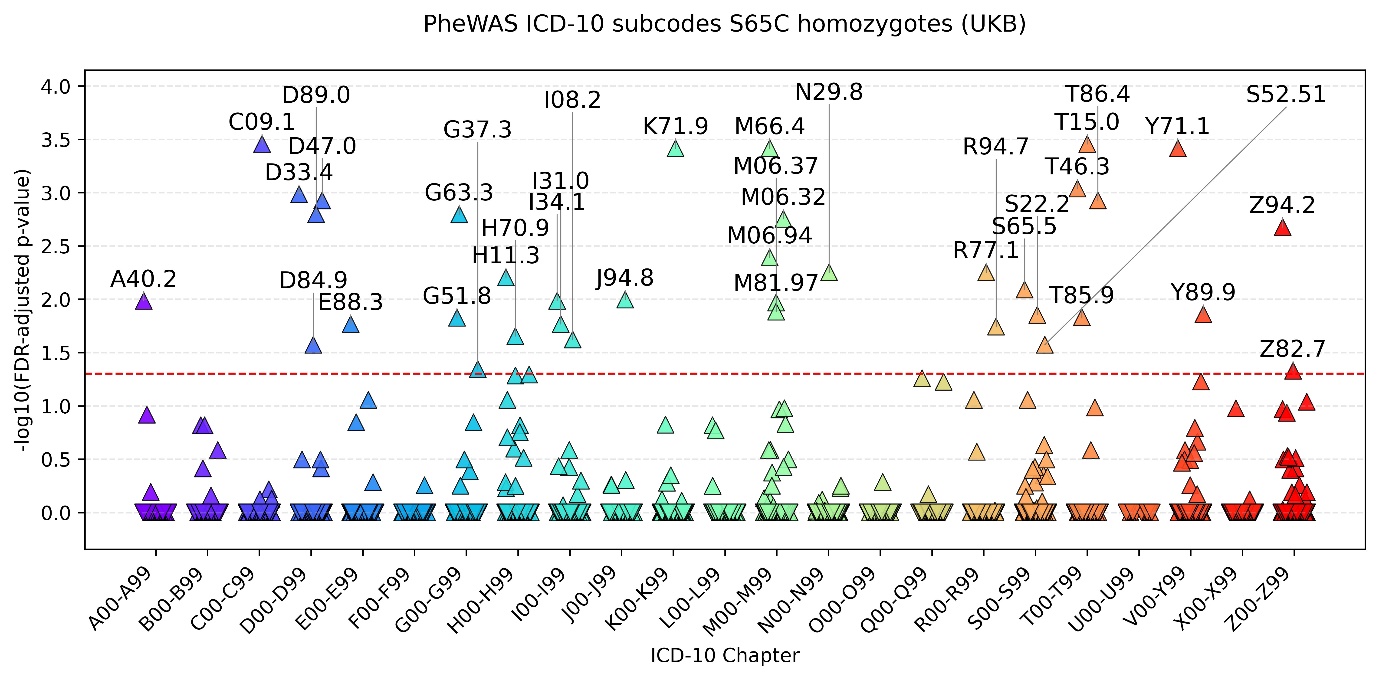


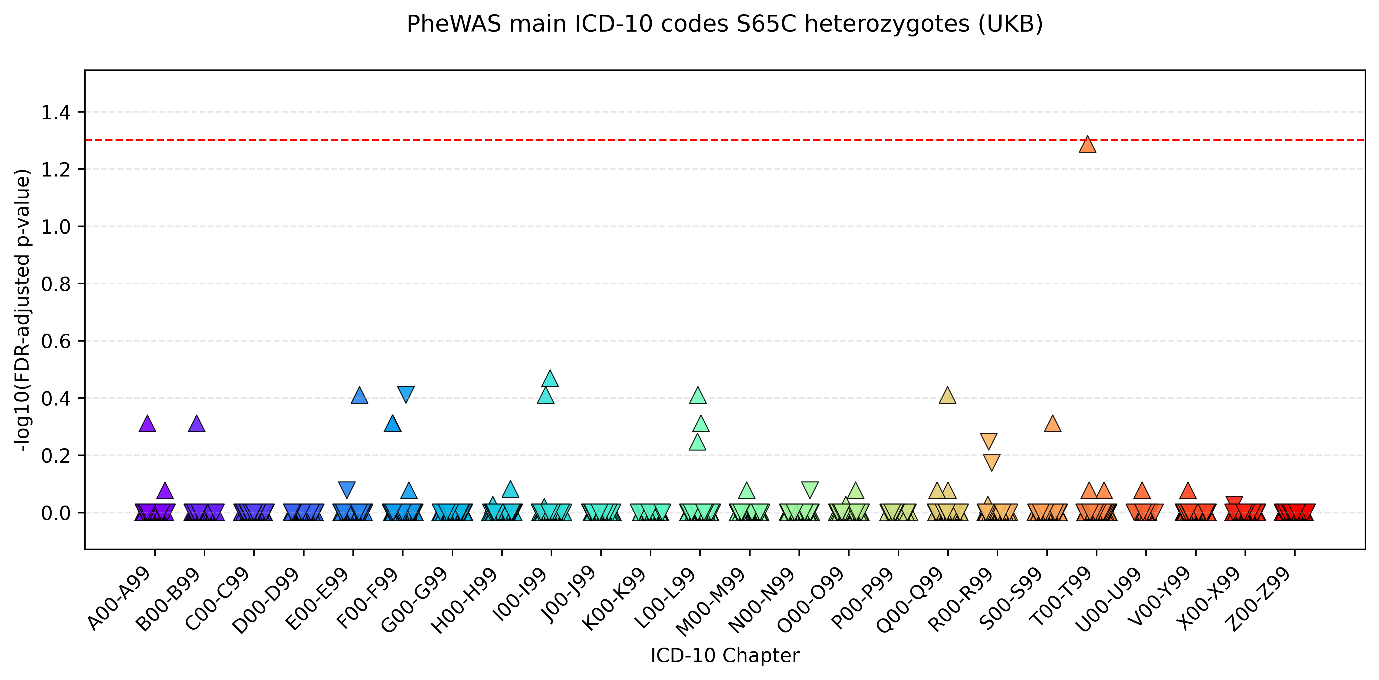

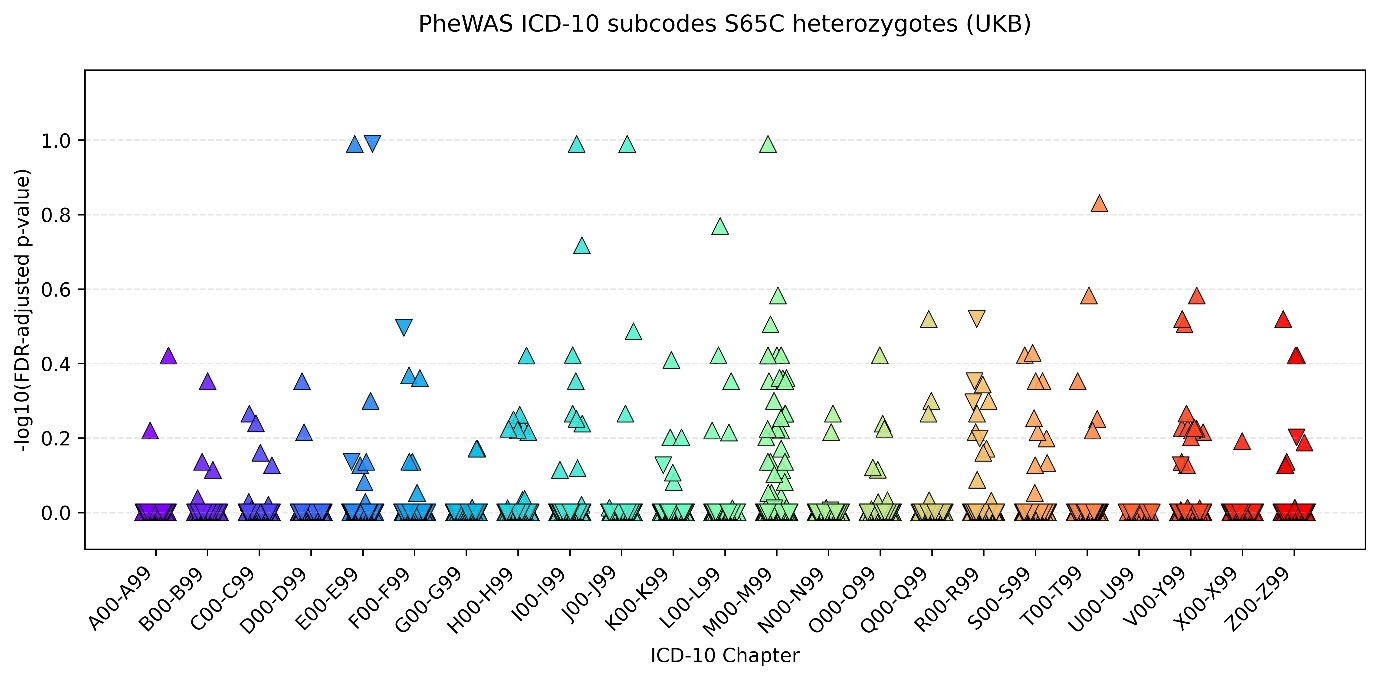


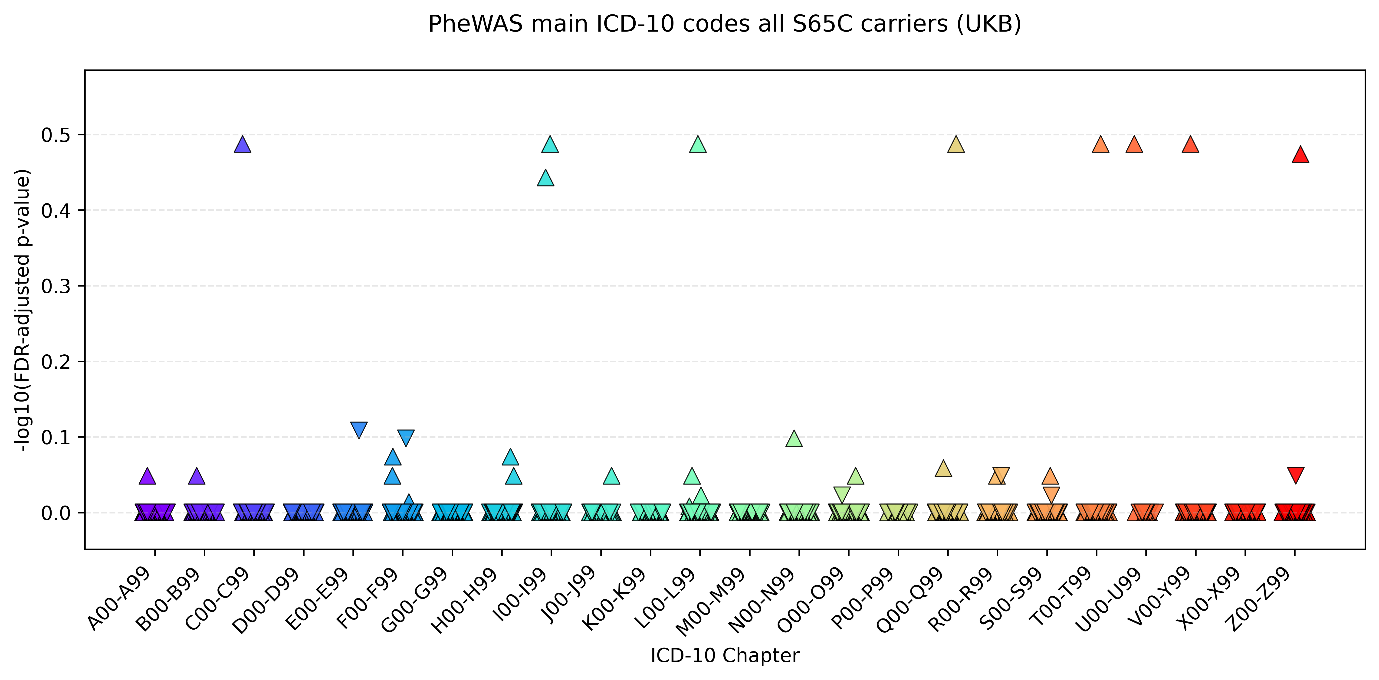

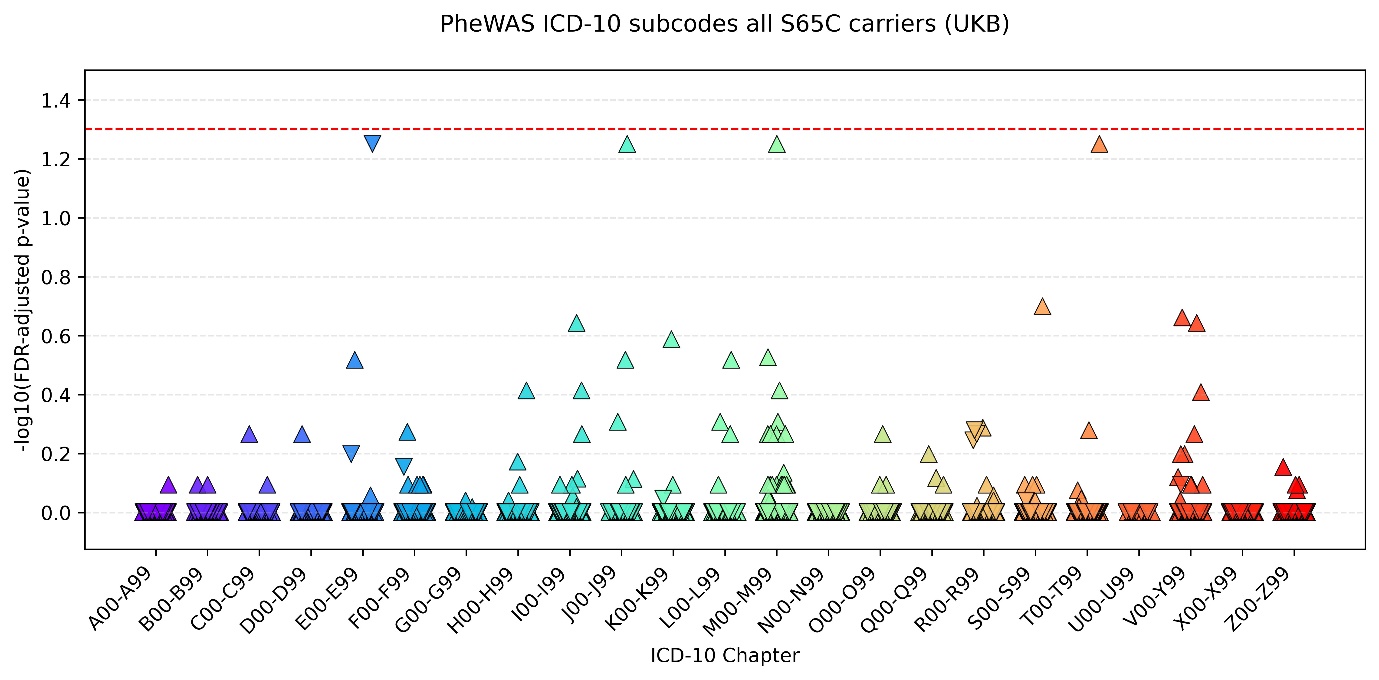


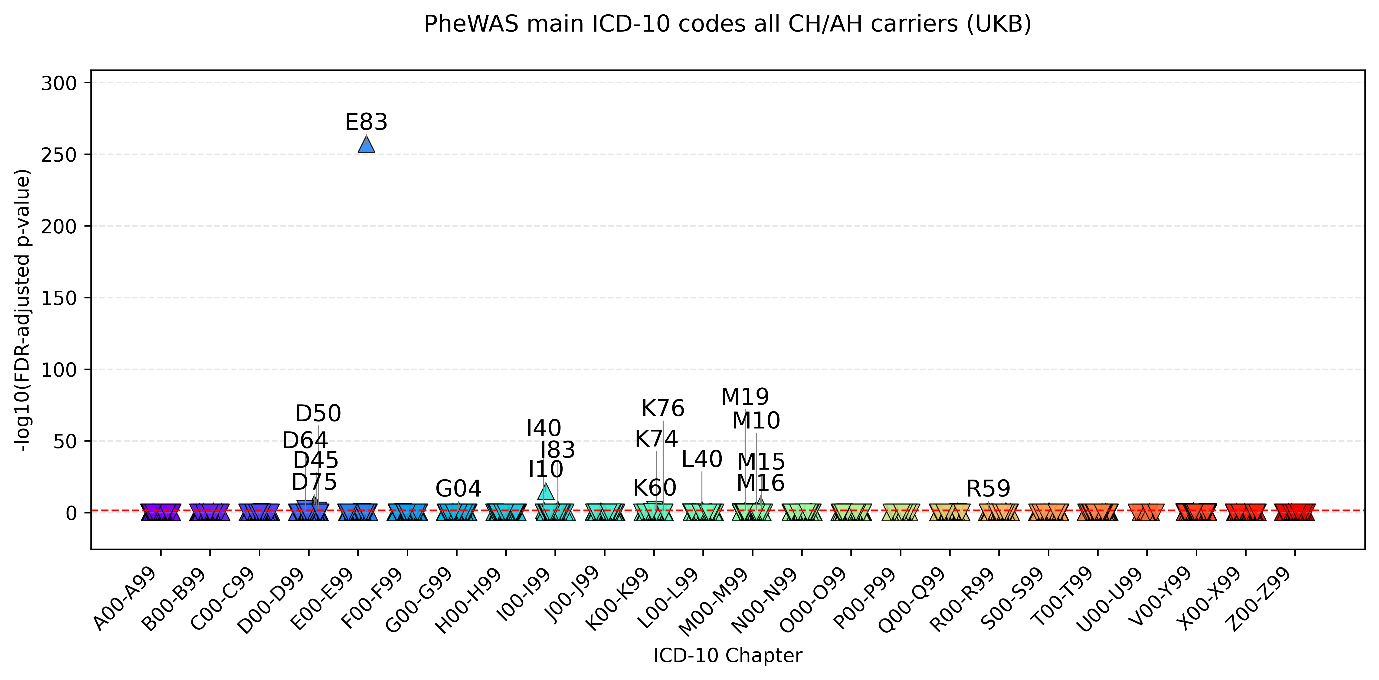

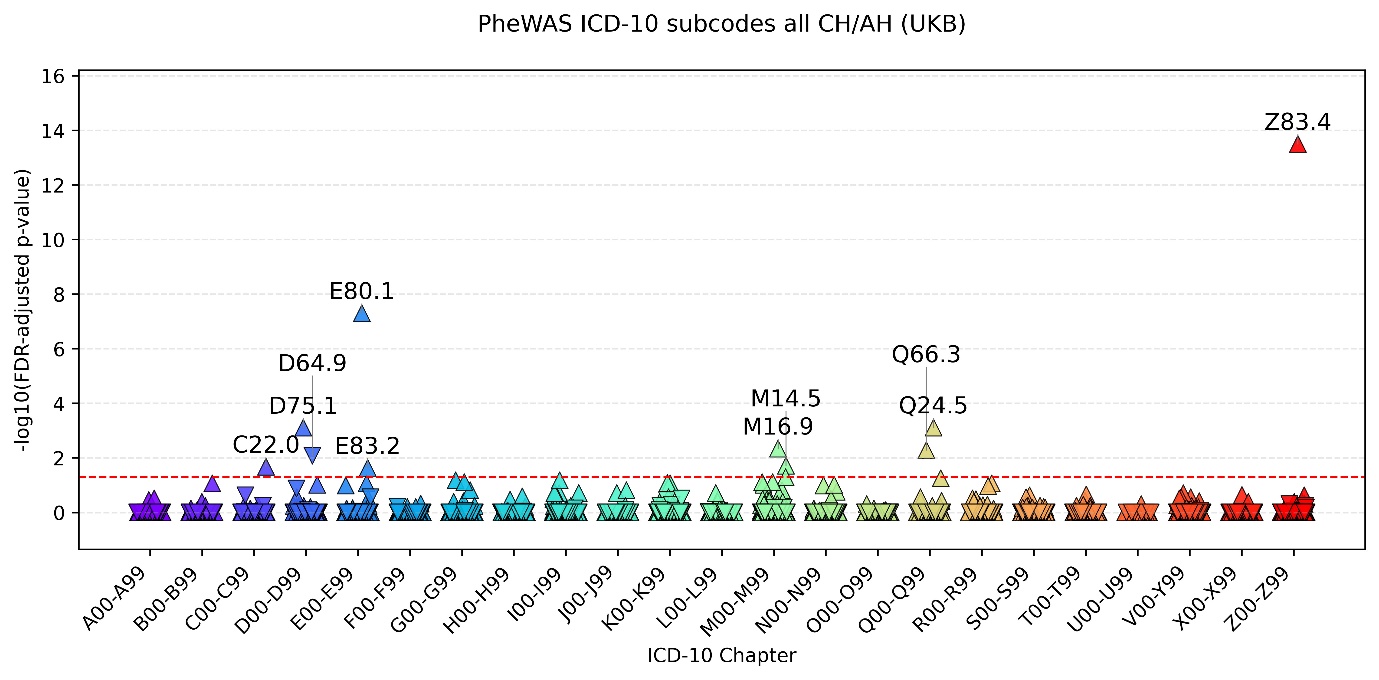


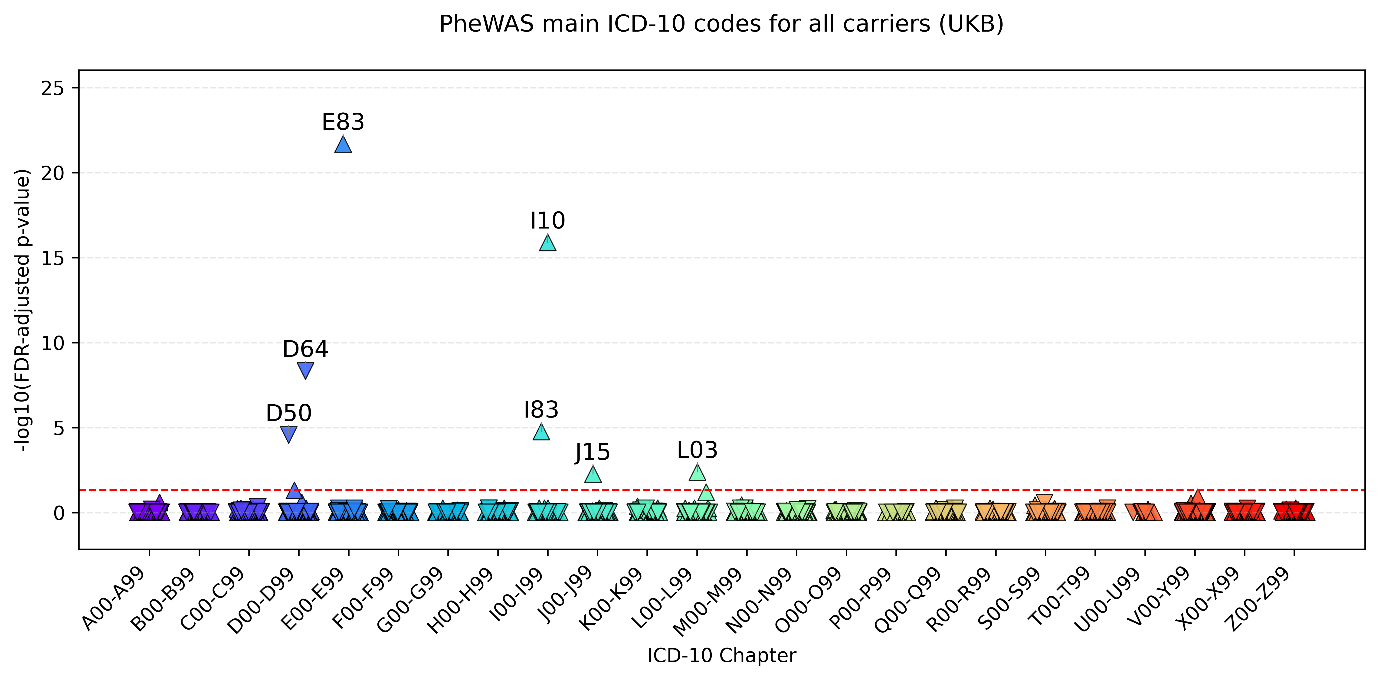

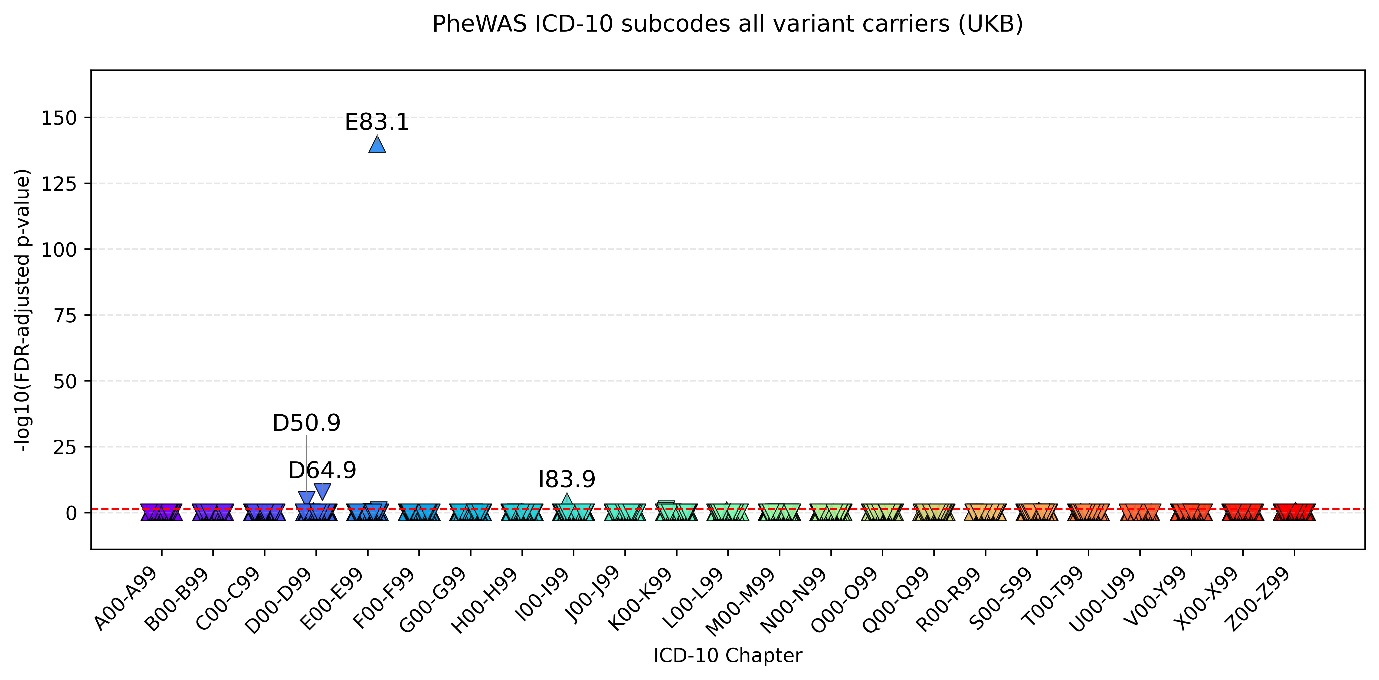


# Supplementary Figs. S57–S59. Manhattan plots of UKB GWAS meta-analysis results.

**Supplementary Fig. S57.** Results of the ceruloplasmin level–based UKB GWAS (*n* = 861). Only SNPs with >1% MAF and INFO scores > 0.8 are shown. MAF, minor allele frequency.


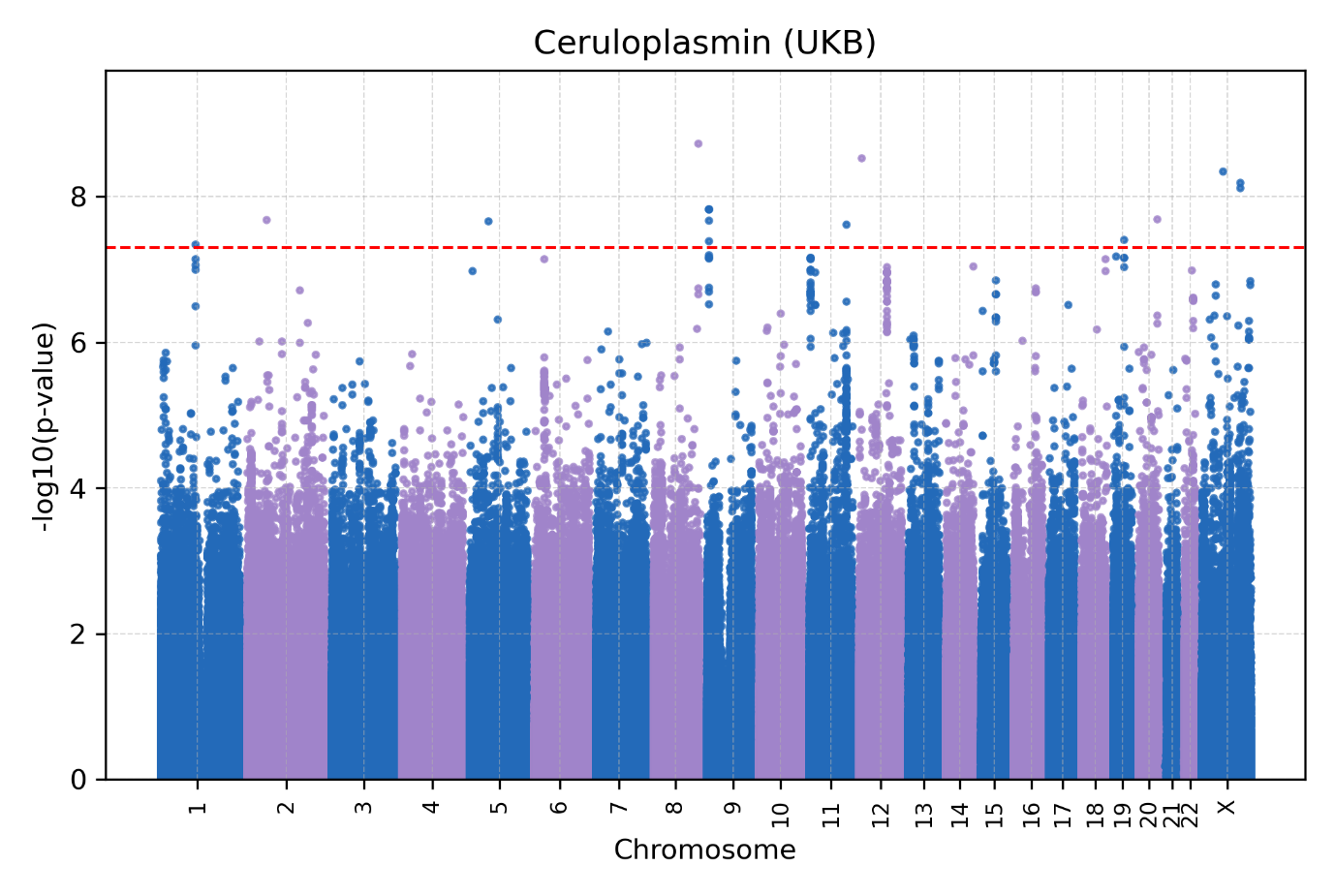
**Supplementary Fig. S58.** Results of the ferritin level–based UKB GWAS (*n* = 81,278). Only SNPs with >1% MAF and INFO scores > 0.8 are shown. MAF, minor allele frequency.
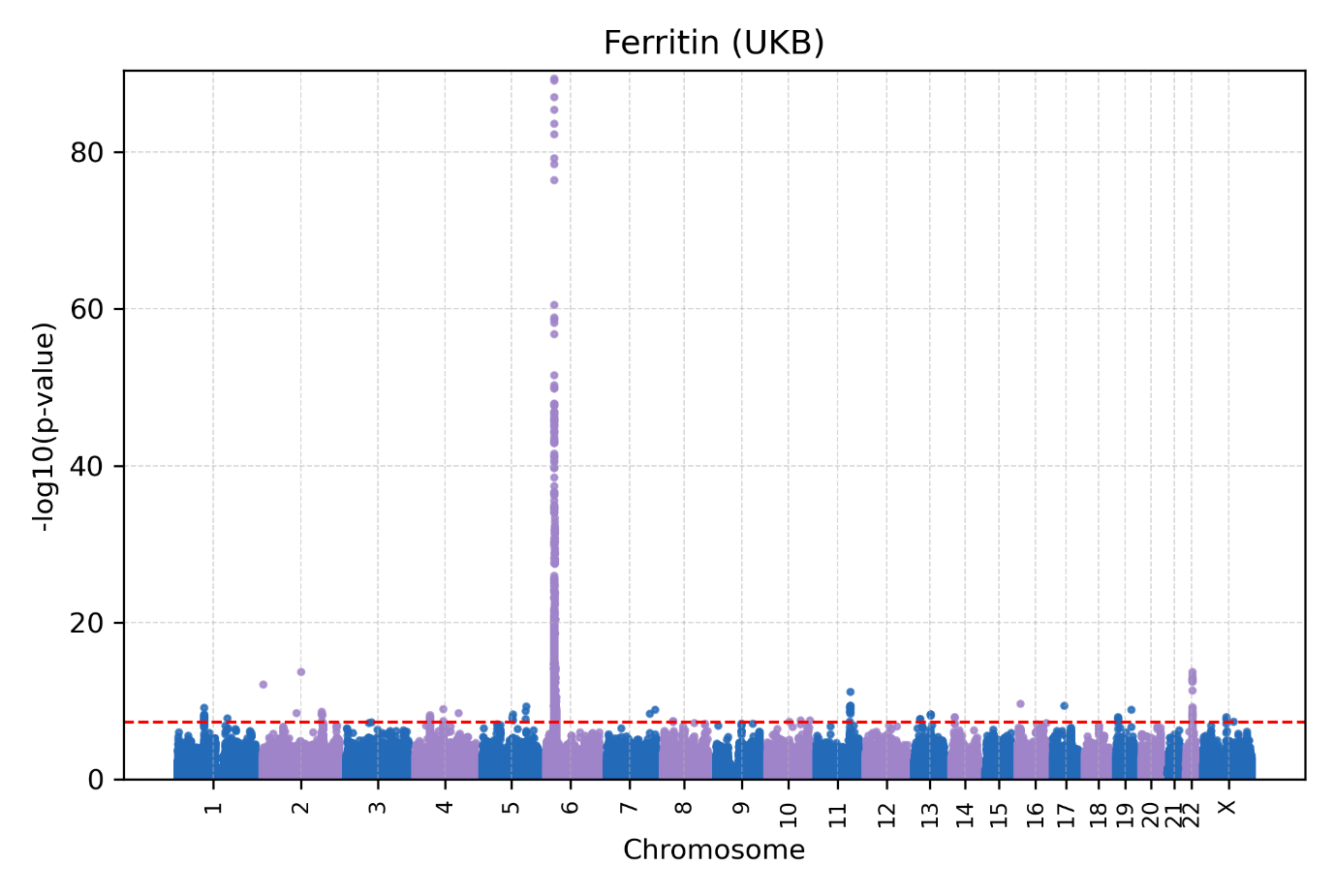


**Supplementary Fig. S59.** Results of the meta-analysis of ceruloplasmin level–based EstBB and UKB GWAS results (only concordant SNPs are shown).


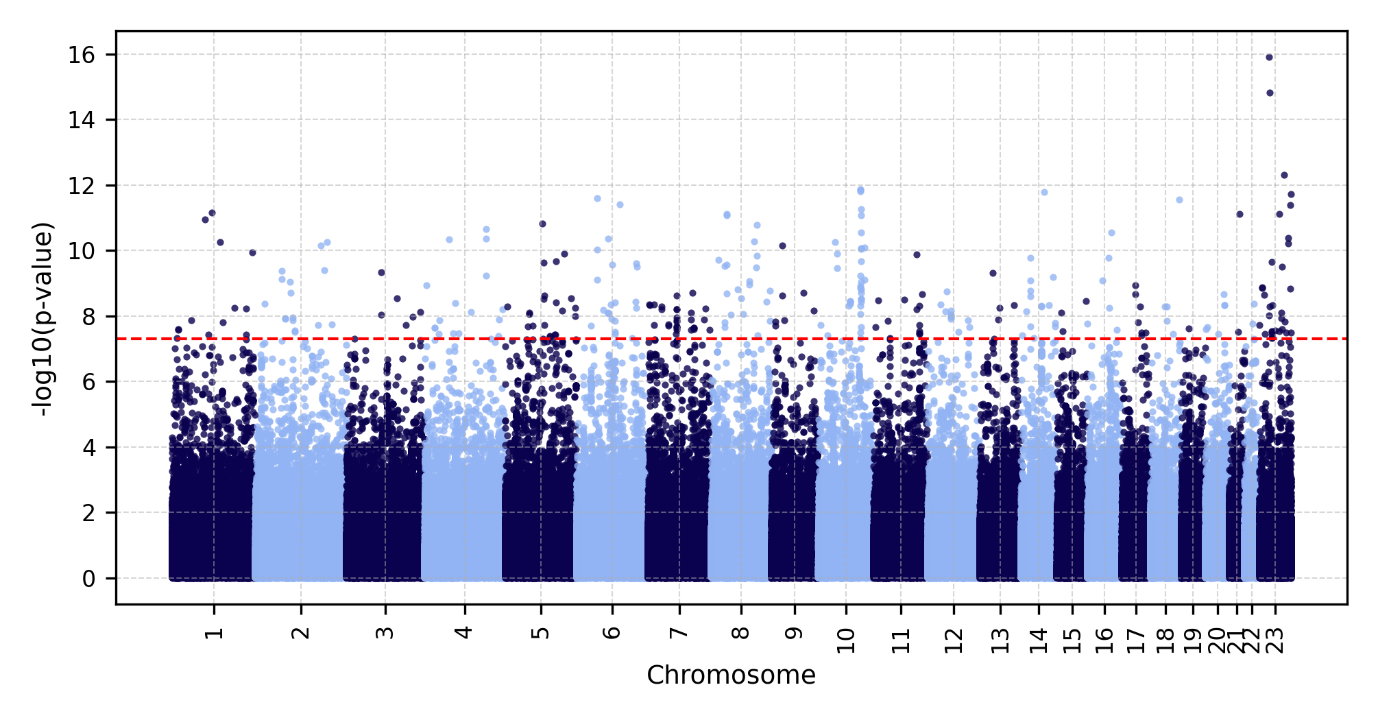


Supplementary Figs. S60–S65. Quantile-quantile plots of GWAS results.

**Supplementary Fig. S60.** Ceruloplasmin level–based EstBB GWAS.


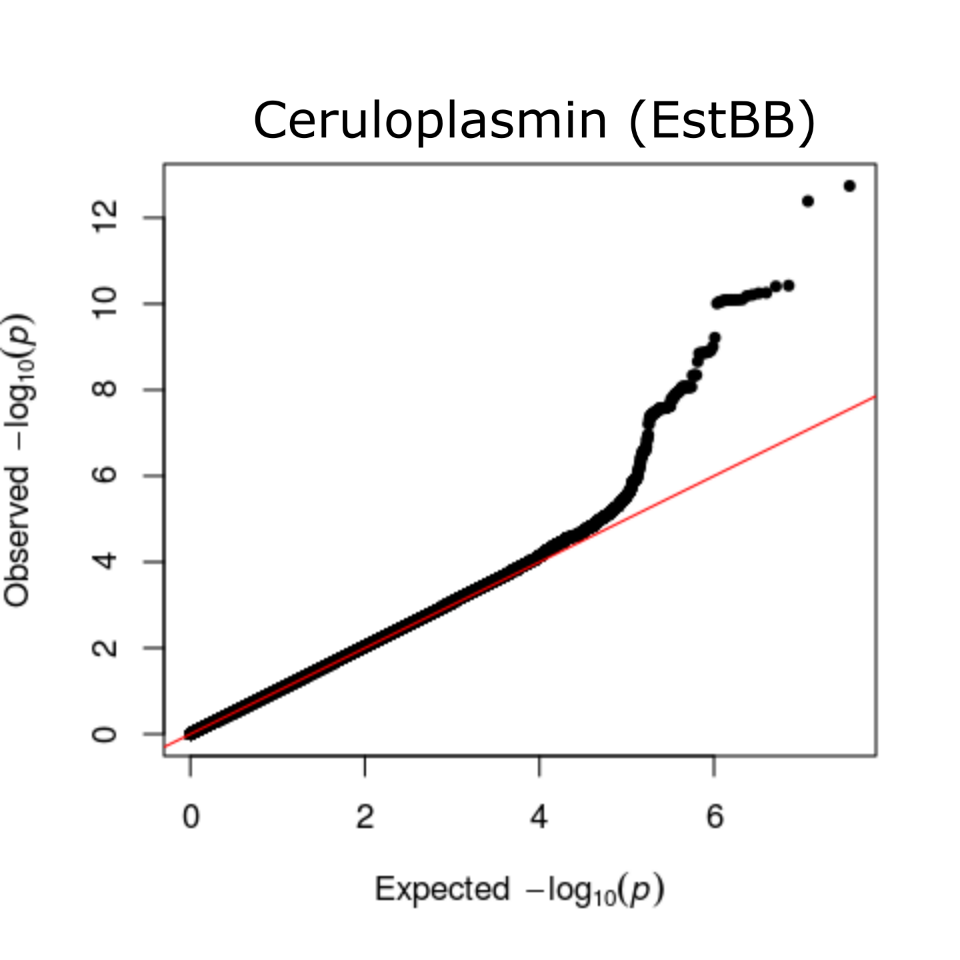


**Supplementary Fig. S61.** Ferritin level–based EstBB GWAS.
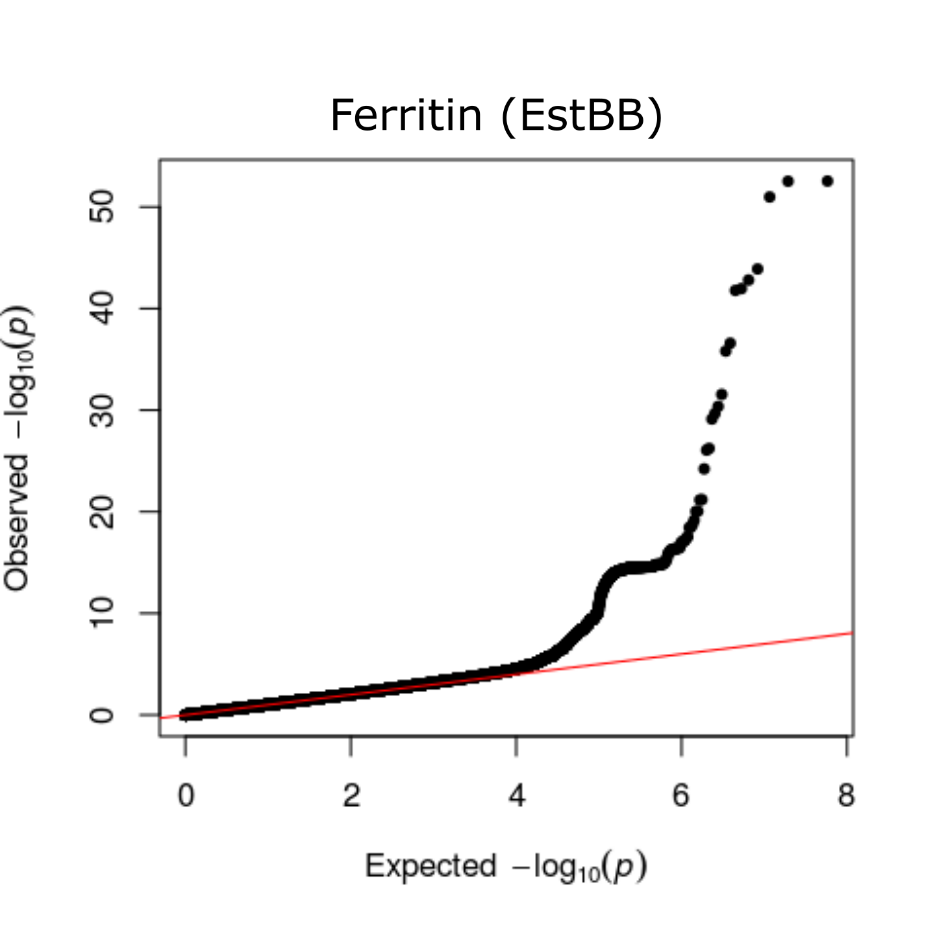


**Supplementary Fig. S62.** Ceruloplasmin level–based UKB GWAS.


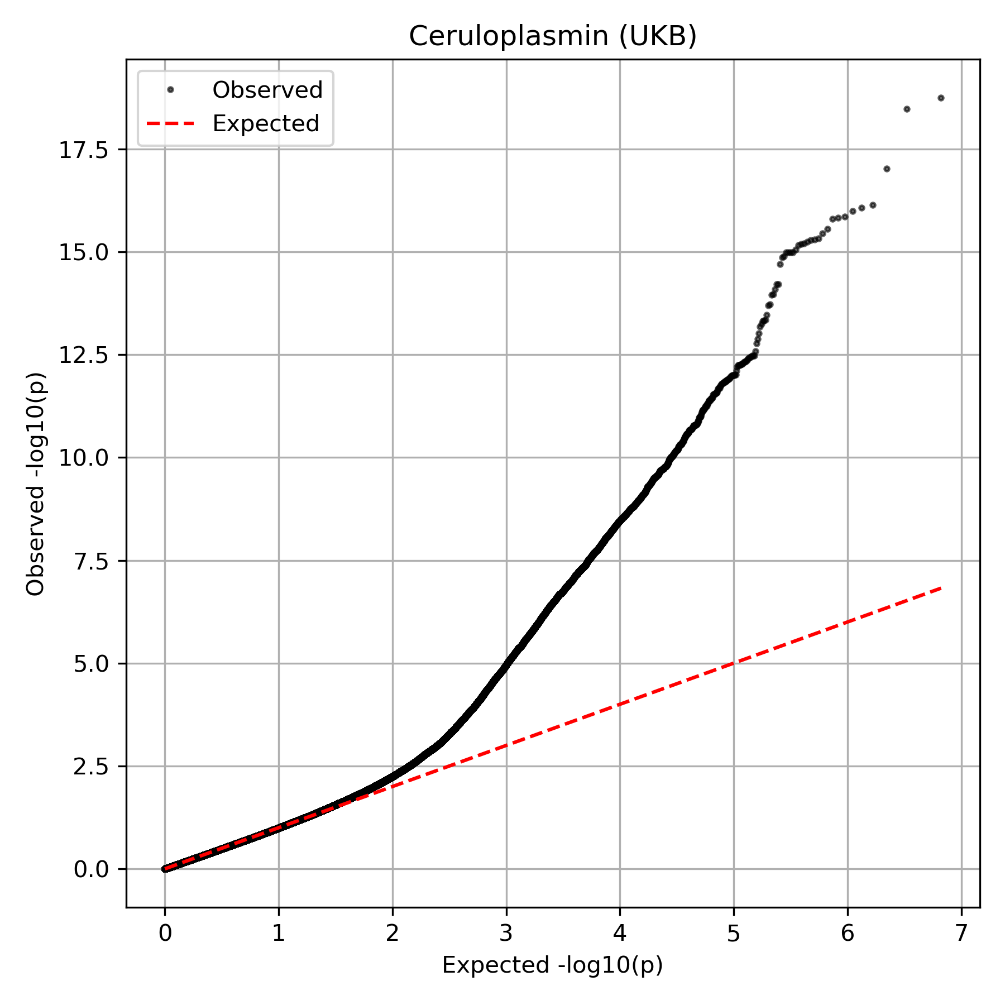


**Supplementary Fig. S63.** Ferritin level–based UKB GWAS.


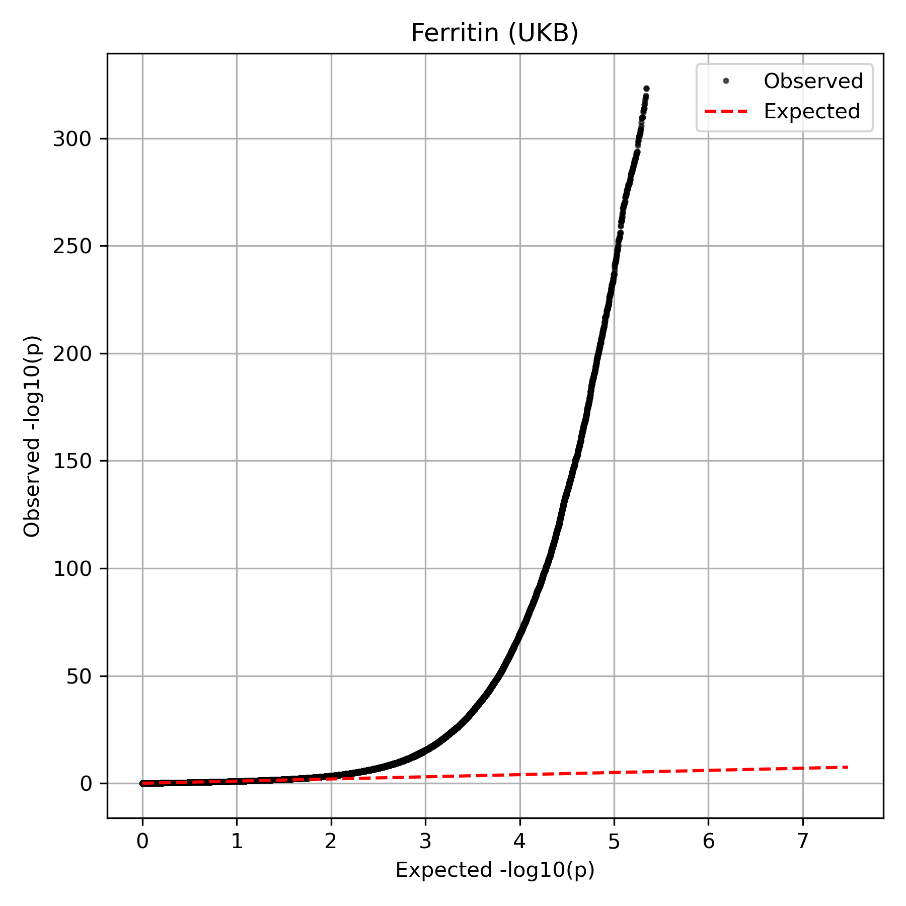


**Supplementary Fig. S64.** Meta-analysis of ceruloplasmin level–based EstBB and UKB GWAS results (only concordant SNPs are shown).


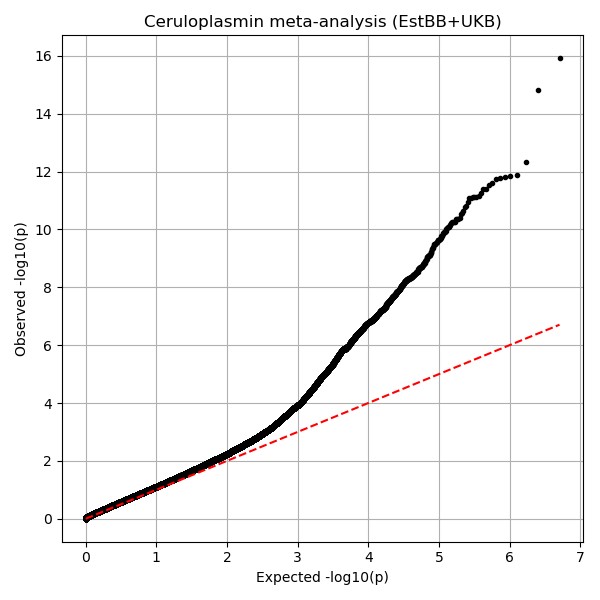


**Supplementary Fig. S65.** Meta-analysis of ferritin level–based EstBB and UKB GWAS results (only concordant SNPs are shown).


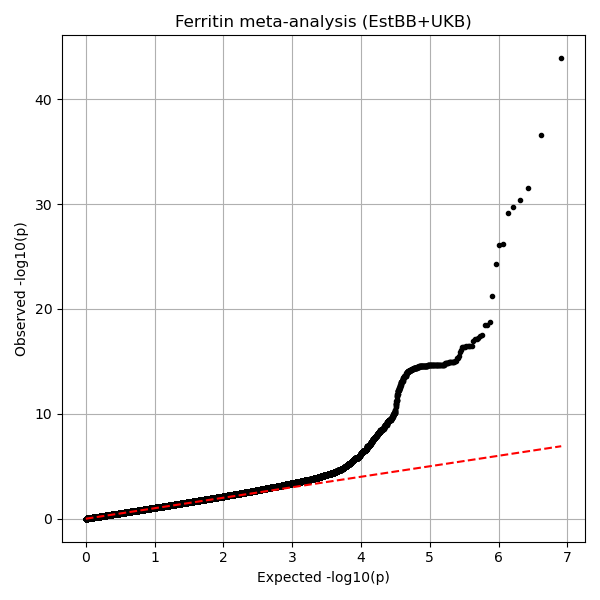

Supplement: Supplementary file 1 — Supplementary Material 1. [file 12864_2026_12746_MOESM1_ESM.docx]
